# Multiple Origins and Specific Evolution of CRISPR/Cas9 Systems in Minimal Bacteria (*Mollicutes*)

**DOI:** 10.3389/fmicb.2019.02701

**Published:** 2019-11-21

**Authors:** Thomas Ipoutcha, Iason Tsarmpopoulos, Vincent Talenton, Christine Gaspin, Annick Moisan, Caray A. Walker, Joe Brownlie, Alain Blanchard, Patricia Thebault, Pascal Sirand-Pugnet

**Affiliations:** ^1^INRA, UMR 1332 de Biologie du Fruit et Pathologie, Villenave d’Ornon, France; ^2^Université de Bordeaux, UMR 1332 de Biologie du Fruit et Pathologie, Villenave d’Ornon, France; ^3^INRA, Mathématiques et Informatique Appliquées de Toulouse, Université de Toulouse, Toulouse, France; ^4^School of Life Sciences, Anglia Ruskin University, Cambridge, United Kingdom; ^5^Department of Pathobiology and Population Sciences, Royal Veterinary College, University of London, London, United Kingdom; ^6^UMR 5800, LaBRI, CNRS, University of Bordeaux, Bordeaux, France

**Keywords:** CRISPR/Cas9, mollicutes, mycoplasma, spiroplasma, phage, mobile genetic elements, horizontal gene transfer, evolution

## Abstract

CRISPR/Cas systems provide adaptive defense mechanisms against invading nucleic acids in prokaryotes. Because of its interest as a genetic tool, the Type II CRISPR/Cas9 system from *Streptococcus pyogenes* has been extensively studied. It includes the Cas9 endonuclease that is dependent on a dual-guide RNA made of a tracrRNA and a crRNA. Target recognition relies on crRNA annealing and the presence of a protospacer adjacent motif (PAM). Mollicutes are currently the bacteria with the smallest genome in which CRISPR/Cas systems have been reported. Many of them are pathogenic to humans and animals (mycoplasmas and ureaplasmas) or plants (phytoplasmas and some spiroplasmas). A global survey was conducted to identify and compare CRISPR/Cas systems found in the genome of these minimal bacteria. Complete or degraded systems classified as Type II-A and less frequently as Type II-C were found in the genome of 21 out of 52 representative mollicutes species. Phylogenetic reconstructions predicted a common origin of all CRISPR/Cas systems of mycoplasmas and at least two origins were suggested for spiroplasmas systems. Cas9 in mollicutes were structurally related to the *S. aureus* Cas9 except the PI domain involved in the interaction with the PAM, suggesting various PAM might be recognized by Cas9 of different mollicutes. Structure of the predicted crRNA/tracrRNA hybrids was conserved and showed typical stem-loop structures pairing the Direct Repeat part of crRNAs with the 5′ region of tracrRNAs. Most mollicutes crRNA/tracrRNAs showed G + C% significantly higher than the genome, suggesting a selective pressure for maintaining stability of these secondary structures. Examples of CRISPR spacers matching with mollicutes phages were found, including the textbook case of *Mycoplasma cynos* strain C142 having no prophage sequence but a CRISPR/Cas system with spacers targeting prophage sequences that were found in the genome of another *M. cynos* strain that is devoid of a CRISPR system. Despite their small genome size, mollicutes have maintained protective means against invading DNAs, including restriction/modification and CRISPR/Cas systems. The apparent lack of CRISPR/Cas systems in several groups of species including main pathogens of humans, ruminants, and plants suggests different evolutionary routes or a lower risk of phage infection in specific ecological niches.

## Introduction

CRISPR systems are natural prokaryotic adaptive immune systems involved in protection against invading DNAs and especially viruses (Hille et al., 2018). Memory of the systems is stored in genomes as typical arrays of spacers interspaced with direct repeats. Effector proteins are encoded by *cas* genes involved in adaptation (acquisition of new spacers), pre-crRNA maturation, and target recognition and cleavage. Since their discovery, and even more, their successful adaptation as molecular scissors for genome engineering in nearly all organisms (Doudna and Charpentier, 2014; Knott and Doudna, 2018), CRISPR systems have been described in about 30–40% bacteria and in most archaea (Koonin et al., 2017). Based on specific signature *cas* genes, CRISPR systems are currently distributed into two classes and 6 types, each of which is sub-divided into up to 7 subtypes. CRISPR systems belonging to Class 2 Type II were the first to be widely used in a large variety of organisms. The iconic spCas9 endonuclease from *S. pyogenes* was used to target DNA by a single guide hybrid RNA designed from the natural structure, which was formed by annealing of matured crRNA and highly structured tracrRNA (Jinek et al., 2012). All Type II systems rely on a Cas9 effector module and a set of other *cas* genes involved in acquisition namely, *cas1*, *cas2* and, in some systems, *cas4* or *csn2*. Based on *cas* genes organization and Cas proteins phylogenies, three Type II systems were identified. Type II-A, which includes the system of *S. pyogenes*, has a typical operon formed by *cas9, cas1, cas2*, and *csn2* genes. In Type II-B, the *csn2* gene is replaced by *cas4*. In Type II-C, neither *csn2* nor *cas4* are present. Expression of Type II systems involves transcription of a precursor crRNA (pre-crRNA) that is subsequently matured by endogenous non-specific RNase III after pairing of pre-crRNA direct repeats with the structured tracrRNA in presence of Cas9. Matured individual crRNA/tracrRNA duplexes, still bound to Cas9, constitute the active complexes involved in targeted DNA cleavage. Target specificity of Type II systems involves recognition of short protospacer adjacent motif (PAM) located downstream of the protospacer on the non-target DNA strand. The PAM sequence is required both in the new spacer acquisition process and for target recognition and cleavage. Because of their relative simplicity and high potential as programmable DNA cleavage tools, a huge amount of data has been obtained on Type II CRISPR systems, including high resolution structures of *S. pyogenes*, *Staphylococcus aureus* and *Campylobacter jejuni* Cas9 in an interaction with gRNA and target DNA (Nishimasu et al., 2014, 2015; Yamada et al., 2017). Despite their popularity, Type II systems are among the rarest found in bacteria with a detection in only 5–7% of bacterial genomes (Chylinski et al., 2014; Bernheim et al., 2019). This type has been detected neither in archaea nor in several bacterial phyla including some photosynthetic bacteria as Cyanobacteria, *Chlorobi*, and *Chloroflexi*, thermophilic bacteria as *Thermotogae*, *Aquificae*, *Deinococcus-Thermus* and intracellular pathogens *Chlamydia*.

Mollicutes are small bacteria, most of which have a parasitic lifestyle, infecting a wide diversity of eukaryotic hosts including humans, many other mammals, birds, reptiles, fishes, arthropods and plants (Gasparich, 2014; Martini et al., 2014; May et al., 2014; Regassa, 2014). In accordance with their parasitic life, these bacteria have lost many metabolic pathways including the ability to synthesize cell-wall components, nucleic acids and protein precursors. Consequently, these minimal bacteria require complex culture media and some of them (phytoplasmas and hemotrophic mycoplasmas) remain uncultured. Evolution of mollicutes has been described as mostly driven by genome reduction, resulting in minute-sized genomes ranging from 0.58 to 2.2 Mbp with an average size around 1 Mbp. Most genera have a specific usage of the UGA codon to encode tryptophan instead of being a stop codon, which might have accentuated a genetic isolation. However, the growing number of genomes available has revealed that mobile elements including plasmids, phages, transposons, and ICE have circulated among mollicutes (Citti et al., 2018). Moreover, comparative genomics and wet-lab experiments have shown large genomic exchanges could occur among mollicutes by horizontal gene transfer (HGT), bringing a new light on the evolution of these minimal bacteria (Dordet-Frisoni et al., 2014; Lo et al., 2015; Baranowski et al., 2018; Tsai et al., 2018; Music et al., 2019). In many bacterial groups, invasion by mobile elements is thwarted by strain-specific systems including restriction-modification (RM), bacteriophage abortive infection mechanisms (Abi), and CRISPR systems. In mollicutes, RM and Abi systems have been described in many species, with various repertoires depending on the strains. More recently, some CRISPR systems were identified in several species. To the best of our knowledge, mollicutes are the bacteria with the smallest genomes where CRISPR systems have been observed (Touchon et al., 2016). In order to get an overview on CRISPR/Cas systems in mollicutes, a global survey was performed using a representative set of 52 mollicutes species.

## Materials and Methods

### Comparative Genomics and Analysis of CRISPR Loci

CRISPR systems were searched in a selection of 52 mollicutes genomes (Supplementary Table S1) available in the MolliGen database (Barré et al., 2004) or in GenBank (Benson et al., 2017) using (i) Blastp (Johnson et al., 2008) search of Cas proteins, (ii) analysis of direct repeats at the CRISPR database (Grissa et al., 2007) with the included CRISPR finder tool (Couvin et al., 2018). A manual analysis of all candidates was then achieved to get a precise annotation of the loci. In order to predict DR/tracrRNA hybrid secondary structure, sequences of DR and tracrRNA were concatenated and the secondary structures of the hybrids were simulated using the mfold software (Zuker, 2003) available at http://unafold.rna.albany.edu/. Cas9 proteins from mollicutes were compared to structurally characterized homologs using HHpred (Soding et al., 2005) and manual inspection of conserved positions. Logos were constructed from direct repeats sequences using weblogo^1^. Consensus direct repeats sequences were submitted to an automated classification process using CRISPRMap tool (Lange et al., 2013).

### Phylogenetic Tree Reconstructions

The phylogenetic tree of the 52 selected mollicutes was generated from the concatenated multiple sequence alignments of 62 selected orthologous proteins involved in translation (Supplementary Table S2), as described in Grosjean et al. (2014). Multiple alignments were generated with MAFFT (Katoh et al., 2017), curated from unreliable positions with GBLOCKS (Talavera and Castresana, 2007), and concatenated with Seaview (Gouy et al., 2010). The final concatenated alignment contained 9,990 sites. The phylogenetic tree was constructed by the Maximum Likelihood method using PhyML (Guindon et al., 2010) (substitution model: WAG, number of categories: 4) available on the web server Phylogeny.fr (Dereeper et al., 2008). For phylogenetic analyses of Cas9 (Supplementary Table S1), multiple alignments were generated using MSAProbs (González-Domínguez et al., 2016) at https://toolkit.tuebingen.mpg.de (FASTA format available in Supplementary Figure S1). Phylogenetic trees were inferred using a Maximum Likelihood method with PhyML (substitution model: WAG, number of categories: 4) available on the web server Phylogeny.fr.

### Spacer Analysis

Several custom databases were built to perform spacer sequences analyses. First, a custom database was assembled from the complete genomes of the NCBI referenced with the Taxonomy identifier as tenericutes (taxid = 544448). 445 complete genomes were retrieved and used to build two custom databases composed of a complete set of CDSs and a collection of intergenic regions, respectively. A prophage database was also built by retrieving sequences from the European Nucleotide Archive (contains 2480 phage sequences) and the prophage and virus database (Last update: 2017) provided by Zhou et al. (2011). Using these three databases, blastn (ncbi-blast-2.7.1 + version) analyses were carried out to search sequences similar to the spacers sequences (562 unique sequences) retrieved from mollicutes CRISPR arrays. An identity of 95% and a coverage of 99% were used as parameters. A total of 12, 569 and 2 hits were retrieved with the CDS, Intergenic and prophage databases, respectively.

### Search for Mobile Genetic Elements in *Mollicutes* Genomes

GenBank genome files of selected mollicutes were downloaded from NCBI Website. (1) Integrative and conjugative elements (ICEs) were identified following the method described in Tardy et al. (2015). Briefly, tblastn searches were conducted using four genes as queries (CDS 5, 17, 19, 22 from *Mycoplasma agalactiae PG2 ICEs* and *Mycoplasma capricolum* subsp. *capricolum ATCC 27343 ICEs*). Hits were manually analyzed and located on the related chromosome. Co-location of the 4 CDS and consistent blast results were interpreted as signs for the presence of complete ICE. Co-location of 2 or 3 CDS and consistent blast results were considered as revealing degraded or atypical ICEs. (2) Insertion sequence were identified using IS finder (Siguier, 2006) at https://isfinder.biotoul.fr/. (3) Restriction systems were predicted using the Rebase database (Roberts et al., 2015) at http://rebase.neb.com/rebase/rebase.html. When several genes were missing, manual check of the region was perform with a Blastp to identify if the system was complete or not. (4) Phage detection was performed using PHAST (Zhou et al., 2011) at http://phast.wishartlab.com/. Additional manual comparisons between mollicutes bacteriophages described in the literature and mollicutes genome sequences were performed using tblastn.

## Results

### CRISPR/Cas Systems Are Present in Most but Not All Mollicutes Groups

**FIGURE 1:**
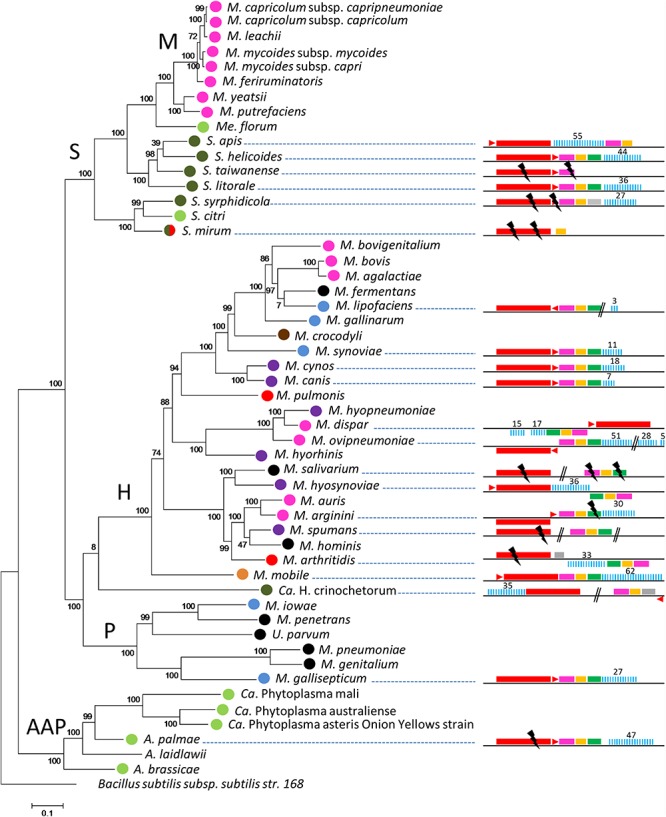
Distribution of CRISPR systems in mollicutes. Organization of the CRISPR systems predicted in mollicutes genomes are represented on the right part. Red rectangle, *cas9*; red triangle, tracrRNA; pink rectangle, *cas1*; orange rectangle, *cas2*; green rectangle, *csn2*; gray rectangle, CDS not related to CRISPR; blue bars, CRISPR array. Above numbers indicate the number of spacers. Double bars, genome interruption; black flash, disrupted gene. The phylogenetic tree was generated using the maximum likelihood method from the concatenated multiple sequence alignments of selected 62 orthologous protein involved in translation. Main phylogenetic groups are indicated; S, spiroplasma; H, hominis; P, pneumoniae; AAP, acholeplasma/phytoplasma; M, mycoides cluster. *B. subtilis* was used as an outgroup. Statistical values from an Approximate Likelihood-Ratio Test are indicated on branches. Hosts are indicated by colored circles; arthropod, dark green, ruminant, pink, rodent, red, bird, blue, others mammals (cats-dogs-pigs), purple, human, black, plant/arthropod, light green, reptile, brown and fish, orange.

CRISPR/Cas systems were first searched in a representative set of 52 mollicutes genomes composed of species from the phylogenetic AAP branch (Acholeplasma-Asteroleplasma-Phytoplasma) and from the three main groups of the SEM branch (Spiroplasma-Entomoplasma-Mycoplasma), namely the Hominis, Pneumoniae and Spiroplasma phyla (Supplementary Table S1). Complete or degraded Type II CRISPR/Cas systems were detected in 21 out of 52 complete or draft genomes of mollicutes (Figure 1). CRISPR/Cas systems including, in the following order, *cas9*, tracrRNA, *cas1, cas2*, and a CRISPR array, were found in most species. Some inversions were observed for several strains such as *Mycoplasma dispar* ATCC27140, *Mycoplasma ovipneumoniae* NM2010, *Mycoplasma hyosynoviae* 232, *Mycoplasma arginini* HAZ145_1, and *Mycoplasma arthritidis* 158L3_1. The overall picture is that CRISPR/Cas systems are detected in most of the main phylogenetic phyla with a more frequent occurrence in the Hominis group. No other type of CRISPR/Cas system other than Type II was predicted.

CRISPR/Cas systems were found to be widespread throughout the Hominis phylum, in the genomes of species infecting a variety of animal hosts, with complete structures predicted in *Mycoplasma synoviae* 53, *Mycoplasma cynos* C142, *Mycoplasma canis* PG14, *M. dispar* ATCC27140, *M. ovipneumoniae* NM2010, *Mycoplasma hyosynoviae* NPL1, *Mycoplasma mobile* 163K and possibly *Mycoplasma lipofaciens* ATCC35015, and incomplete structures in *Mycoplasma salivarium* ATCC23064, *Mycoplasma spumans* ATCC19526, *Mycoplasma arthritidis* 158L3_1 as well as the outgroup *Ca*. Hepatoplasma crinochetorum Av.

In the Pneumoniae phylum, a CRISPR/Cas system was found only in the bird pathogen *Mycoplasma gallisepticum* S6. For this species, complete or incomplete forms of the CRISPR locus have been characterized in the 12 genomes available (Supplementary Figure S2). Depending on the strain, CRISPR arrays included 23 to 105 spacers and their evolution has been previously associated with a recent host shift from poultry to American house finches (*Haemorhous mexicanus*) (Delaney et al., 2012; Tulman et al., 2012).

In the Spiroplasma phylum, putatively complete CRISPR/Cas systems were found in *Spiroplasma helicoides* TABS-2 and *Spiroplasma litorale* TN-1 whereas more or less degraded forms were found in *Spiroplasma taiwanense* CT1 and *Spiroplasma mirum* ATCC29335. In *Spiroplasma syrphidicola* EA1 and *Spiroplasma apis* B31, all the above mentioned genetic elements were predicted (complete or disrupted), except *csn2* that remained undetected. Remarkably, a second CRISPR locus including an array of 9 direct repeats and 8 spacers was detected in S. *syrphidicola* EA1 genome but *cas* genes could not be identified, whereas a putative tracrRNA was predicted (not shown). No CRISPR/Cas system could be detected in *Spiroplasma citri* GII-3 and other closely related species (i.e., *S. kunkelii* and *S. melliferum*, not shown). In the AAP branch, a degraded CRISPR/Cas system was found in *Acholeplasma palmae* J233 but not in the 11 other acholeplasma genomes available (extended search on all genomes available in June 2019).

Remarkably, some groups of species belonging to the same phylogenetic branch or associated with the same host appear to lack CRISPR/Cas systems, at least from the currently available genomes. Most noteworthy examples are human pathogenic mollicutes infecting pulmonary and urogenital tracts. Despite a growing number of genome sequences (extended search on all genomes available in June 2019), no CRISPR/Cas were found in *M. pneumoniae* (90 genomes available), *M. genitalium* (6), *M. hominis* (23), *M. penetrans* (2), *U. urealyticum* (18), and *U. parvum* (14). CRISPR/Cas systems also remained undetected in the ruminant pathogens belonging to the Mycoides cluster (Spiroplasma phylum and 35 genomes available) as well as the *M. agalactiae/M. bovis* group (Hominis phylum and 39 genomes available), they were, however, detected in other ruminant mycoplasmas, namely *M. ovipneumoniae*, *M. dispar* and *M. arginini*. Finally, no CRISPR/Cas system was predicted in the uncultivated and plant pathogenic phytoplasma (AAP phylum, 26 genomes available).

Therefore, Type II CRISPR/Cas systems appear to be widespread among mollicutes with some particular groups currently lacking any trace of these microbial defense mechanisms.

### CRISPR/Cas Systems From Mycoplasmas Have a Common Origin but Not Those From Spiroplasmas

**FIGURE 2:**
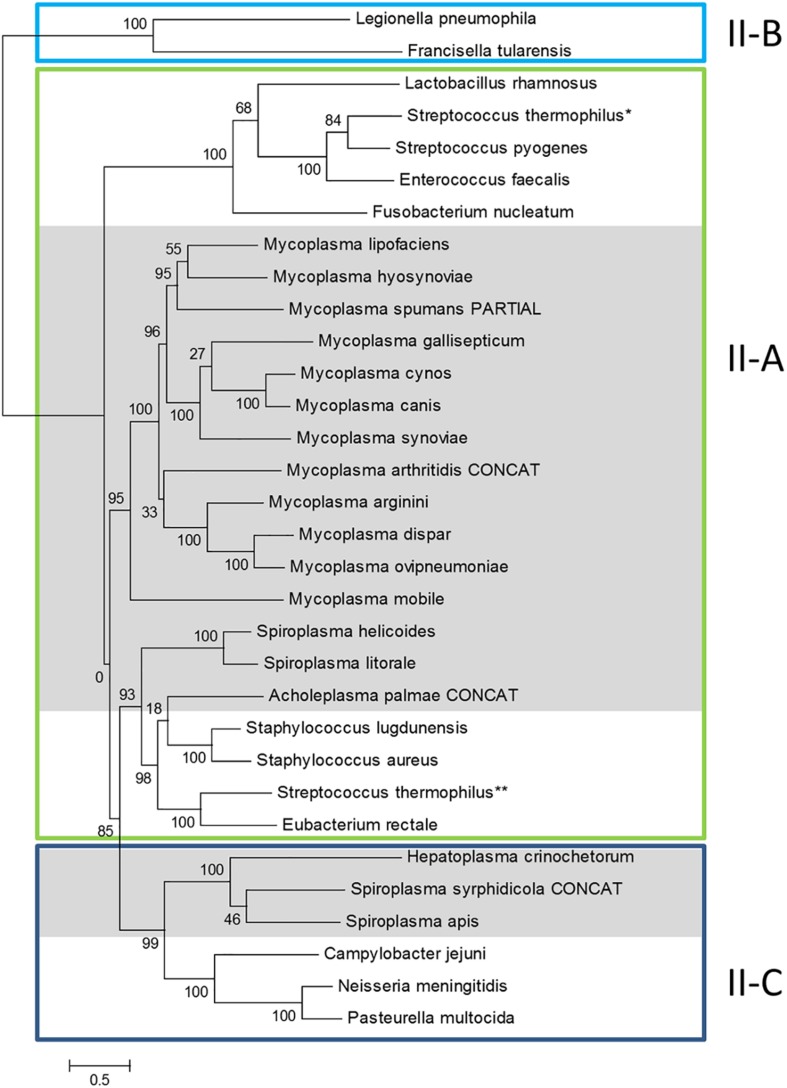
Phylogeny of Cas9 proteins in mollicutes and reference bacteria. Amino-acid sequences of Cas9 proteins were aligned with MSAProbs and phylogenetic tree was reconstructed with PhyML with tools available on phylogeny.fr. For *M. arthritidis*, *A. palmae* and *S. syrphidicola*, Cas9 protein sequences were artificially simulated from fusions of ORFs covering the disrupted gene (indicated with CONCAT). Cas9 from reference bacteria were chosen from Fonfara et al. (2014); proposed subtypes were also defined according to this work; ^∗^ and ^∗∗^ refer to the two Cas9 from *S. thermophilus* also described in this work. Cas9 from mollicutes are highlighted in gray. Cas9 accession numbers are available in Supplementary Table S1.

Previous phylogenomic studies on the diversity of bacterial CRISPR/Cas systems including some mycoplasma systems, have classified them as Type II CRISPRs, with all representants studied gathering in a specific branch of subtype II-A (Fonfara et al., 2014). In order to get a more complete picture, we performed similar phylogenomics focusing on mollicutes CRISPR/Cas systems. Amino-acid sequence of Cas9 from mollicutes were aligned together with a set of reference Cas9 proteins from subtypes II-A, II-B and II-C, as defined in Fonfara et al. (2014) (Supplementary Table S1). A phylogenetic tree was then infered from the multiple alignment (Figure 2).

General topology of the Cas9 tree only partially reflects species phylogeny with proteins of several mollicutes species clustering with non-mollicutes homologs. However, proteins Cas9 from all mycoplasmas clustered in a statistically highly supported branch (aLRT value, 95%). This suggested a common origin of all CRISPR/Cas systems currently identified in mycoplasma species. Inner branches only partially reflected species phylogeny. For exemple, Cas9 from *M. gallisepticum* S6 was found in a 100% supported subgroup including *M. synoviae* 53, *M. cynos* C142 and *M. canis* PG14. While those last three species are phylogenetically closely related in the Hominis group (see Figure 1), *M. gallisepticum* is a remote species from the Pneumoniae group. Similarly, Cas9 from *M. lipofaciens* ATCC35015 clustered with homologs found in relatively remote species *M. hyosynoviae* NPL1 and *M. spumans* ATCC 19526. Cas9 from other mollicutes were found more widely distributed in the phylogenetic tree. Cas9 from *A. palmae* J23 was found in a branch with homologs from several reference Gram positive bacteria (staphylococci, *Streptococcus thermophilus, Eubacterium rectale*) which is consistent with their common ancestral origin. Cas9 orthologs from Spiroplasmas and *Ca*. H. crinochetorum Av were distributed in two remote subgroups with no correlation to their relative phylogenetic position. Indeed, Cas9 from *S. apis* B31 was found closely related to that of *S. syrphidicola* EA1 whereas those two spiroplasmas belong to two clearly disctinct phylogenetic subgroups. By contrast, Cas9 from *S. helicoides* TABS-2 appeared remote from that of *S. apis* B31 while those two species are very closely related. These observations suggested different origins for the CRISPR/Cas systems found among spiroplasmas. In addition, we noticed that no trace of *csn2* gene could be predicted in the genomes of *S. apis* B31, *S. syrphidicola* EA1 and *Ca*. H. crinochetorum Av. Interestingly, Fonfara et al. have proposed a fine classification of Type II CRISPR/Cas systems with a subtype II-C characterized by the lack of *csn2* or *cas4* that are found in subtypes II-A and II-B, respectively (Fonfara et al., 2014). This suggested that the CRISPR/Cas systems of these three mollicutes might be evolutionarily related to subtype II-C systems by contrast to all other CRISPR/Cas systems from mollicutes for which a *csn2* gene has been predicted.

### Cas9 Proteins of Mollicutes and *S. aureus* Have Similar Domains Except the PAM-Interacting Domains

We compared predicted Cas9 proteins from mollicutes with structurally characterized Cas9 using HHpred. In all cases, the best hit was SaCas9 from *S. aureus* (PDB ID 5CZZ), with 100% probabilities and *e*-values ranging from 4.9E-146 to 2.6E-103. HHpred alignments were extended over all the proteins except for the Type II-C Cas9 of *S. apis* B31, *S. syrphidicola* EA1 and *Ca*. H. crinochetorum Av for which the highly significant alignments stopped after the RuvC-III domains. Still with SaCas9, similarity ranged from 31% to 58%, with the best similarities observed for the Cas9 of *A. palmae* (concatenation of the two protein parts predicted from the ORF interupted by a mismatch), *S. helicoides* and *S. litorale*. This was in accordance with their phylogenetic related positions in the Cas9-infered phylogenetic tree (Figure 2). Structural domains of each of the Cas9 of mollicutes were infered from the pairwise HHpred alignments with SaCas9 (Supplementary Figures S1, S3). Cas9 proteins from mollicutes are larger than SaCas9 (1059 aa) but smaller than SpCas9 (1368 aa), with sizes ranging from 1069 aa to 1272 aa. Compared to SaCas9, conserved domains with larger sizes are the REC (recognition) lobe and to a lesser extend the L1, RuvC-III and WED domains, all involved in the NUC (nuclease) lobe.

The most conserved regions among Cas9 of mollicutes are also similar to SaCas9, suggesting key positions involved in the protein structure, furthermore the interactions with gRNA and target DNA might be globaly conserved. A more detailed analysis was undertaken (Supplementary Material SM1), based on the key amino acids identified from the crystal structure of SaCas9 (Nishimasu et al., 2015). Overall, many key residues involved in the interaction between Cas9 and the gRNA/target DNA hybrid are conserved among mollicutes Cas9. By contrast, the amino acids from the WED and PI domains involved in PAM duplex recognition are not conserved within mollicutes Cas9 and nor when compared to SaCas9, with the exception of Tyr789 that is found strictly conserved among Type II-A Cas9 but not Type II-C (Supplementary Figure S4). Globally, the protein regions involved in PAM duplex recognition in SaCas9 were found very variable among mollicutes with many indels and non-similar amino acid changes.

### Direct Repeats/tracrRNA Hybrids of Mollicutes CRISPR/Cas Systems Form Typical Structures but With Highly Variable Sequences

**FIGURE 3:**
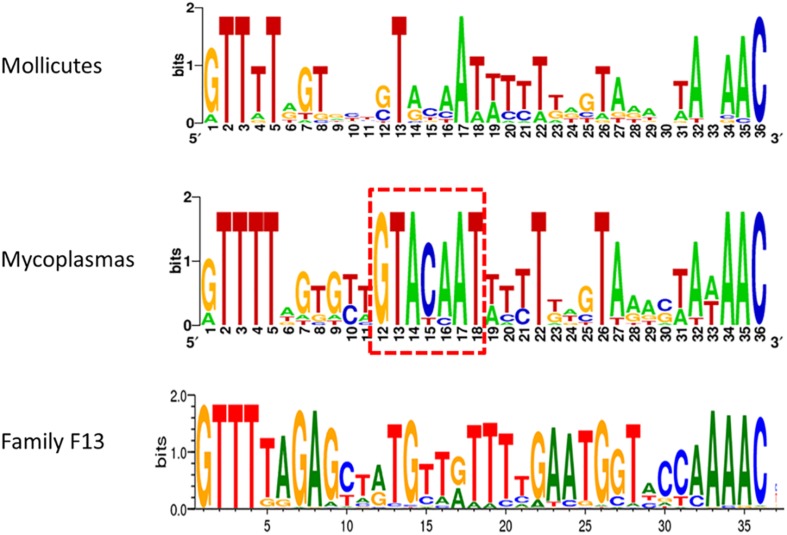
Consensus sequence of Direct Repeats of mollicutes and mycoplasmas CRISPR. DR sequences from 17 CRISPR systems of mollicutes were used to create a weblogo at http://weblogo.berkeley.edu/. These logos were somewhat similar to the one from Family F13 of DR defined in CRISPRMap. The internal motif conserved at positions 12–18 in most mycoplasmas is framed in red dots.

Typical CRISPR arrays with direct repeats (DR) interspaced with unique spacers were predicted in all mollicutes with a CRISPR/Cas system, with the exception of *S. mirum* ATCC29335, *M. salivarium* ATCC23064 (draft genome) and *M. spumans* ATCC19526 (draft genome). Depending on the species and the strain, the number of spacers was found highly variable, from 3 spacers in *M. lipofaciens* ATCC35015 (draft genome) up to 105 in *M. gallisepticum* (strain R_high_). Consensus sequences of DR were determined for 17 mollicutes CRISPRs, showing an identical length of 36 bp (Supplementary Table S3). A logo plot was designed (Figure 3) showing some conserved positions on both sides of the motif as well as a few positions inside the motif. The logo plot designed from the 11 DR from mycoplasmas evidenced an internal motif GTACAAT conserved at position 12–18 in all species except *M. mobile* 163K. All 17 consensus DR sequences from mollicutes were submitted to an automated classification process using CRISPRMap tool. By comparing mollicutes DR with a database of 4719 consensus repeats covering 24 families and 18 structural motifs, CRISPRMap assigned all DR to superclass F except DR from *Ca*. H. crinochetorum which was not assigned. Superclass F gather DR from various bacteria with a high level of sequence diversity. Notably, this superclass includes Family F13 to which DR from the typical *S. pyogenes* CRISPR/Cas Type II system has been assigned.

**FIGURE 4:**
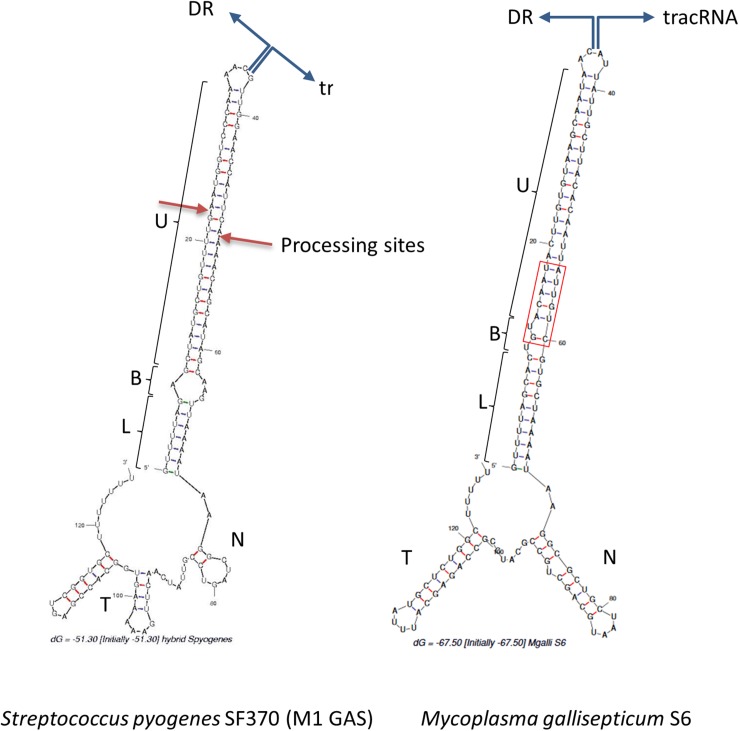
Predicted DR/tracrRNA hybrid secondary structure. Sequences of DR and tracrRNA were concatenated and the secondary structures of the hybrids were simulated using the mfold software at http://unafold.rna.albany. edu/. Position of the DR/tracrRNA concatenation is indicated by divergent blue arrows. Predicted stems involving DR and tracrRNA anti-repeats include a lower stem (L), a bulge (B), and an upper stem (U). For *S. pyogenes*, processing sites of the natural hybrid by RNAse III are indicated by red arrows. For *M. gallisepticum* S6, DR and tracrRNA sequences were defined based on (Chylinski et al., 2014) and our own work. N, nexus stem-loop; T, terminator. The base of the upper stem loop that is highly conserved in mycoplasmas is framed in red.

In Type II CRISPR, pairing between the typical antirepeat region of the tracrRNA and the DR from the pre-crRNA is essential for the RNAse III processing of the hybrid structure into a mature dual-guide RNA (Deltcheva et al., 2011). In order to predict tracrRNA/DR interactions in mollicutes CRISPRs, secondary structures of virtual RNA molecules consisting in the 36bp-long DR sequence concatenated with predicted tracrRNA were simulated using the mfold program. As a control, the same process was applied to concatenated DR and tracrRNA from the typical *S. pyogenes* CRISPR01 system. Secondary structure predicted for this was in accordance with previous work (Deltcheva et al., 2011), showing a nearly perfect pairing of DR with the 5′ region of tracrRNA (Figure 4). Similarly, long stem-loops involving both RNA molecules were predicted for the 15 mollicutes studied, as exemplified for *M. gallisepticum* S6, *S. apis* B31, *A. palmae* J233, *M. lipofaciens* ATCC35015 *and M. synoviae* 53 (Figure 4 and Supplementary Figure S5). As shown for other Type II-A systems, direct repeat/antirepeat stem can be divided in a lower stem, a bulge and a upper stem. The predicted hybrid structure for *S. apis* B31 is highly similar to that of other mollicutes despite the relatively remote position of this Type II-C CRISPR/Cas system. Remarkably, no bulge or mispairing was observed in the structure predicted in *M. synoviae* 53 and *Ca*. H. crinochetorum Av (not shown).

Regions of the Type II tracrRNA dowstream of the hybrid include the nexus stem-loop and a Rho-independent terminator ending with a poly-U track. Those typical features were predicted for all mollicutes but with a remarkable diversity of sequences and stem lengths (Supplementary Table S4).

Multiple alignment of mollicutes tracrRNAs showed very low conservation except a few positions in the AT-rich base of the lower stem and the GC-rich base of the nexus. The relative conservation of these particular regions of mollicutes tracrRNAs is consistent with general pattern observed in Type II tracrRNAs (Faure et al., 2019). In particular, when focusing on the mycoplasma genus, the upper stem part adjacent to the bulge (when existing) is highly conserved with the GUACAAU motif from the DR paired with a AUUGUAC motif in the antirepeat part of tracrRNAs in most species. For other mollicutes as spiroplasmas and acholeplasma, pairing of this region has been maintained through evolution, despite significant sequence divergence. The conservation of this part of the hybrid suggests a selective pressure for the co-evolution of DR and tracrRNA, as it has been noticed in other groups of bacteria.

The lower stem was found to start with a G:U wooble base pair in only 7 mollicutes dual-gRNAs out of 15, whereas this was shown to be the case in the vast majority of Type II-A dual-gRNAs (Faure et al., 2019). By contrast, a G:C and a A:U pair were predicted for *A. palmae* J233 (Supplementary Figure S5) and *M. hyosynoviae* NPL1, respectively. Moreover, the one or two first bases starting the DR immediately dowstream to the spacer were predicted unpaired for *S. helicoides* B31, *S. litorale* TN-1, *M. cynos* C142, *M. canis* PG14, *M. arginini* HAZ145_1, and *M. mobile* 163K. These uncommon features were conserved in closely related species (*S. helicoides* TABS-2 and *S. litorale* TN-1, *M. cynos* C142 and *M. canis* PG14), suggesting no sequencing artifact or specific mutation had occurred. Therefore, unpaired bases might reflect secondary structure inaccurate predictions or a particular flexibility in the dual-gRNA structures of those mollicutes.

The junction between the hybrid region and the nexus is AA in 8 (possibly 11) species, as reported for most Type II-A. This observation is consistent with the previously mentioned conservation of Asn780 and Leu783 that are known to interact the first A (A55) of this junction. However, other sequences (i.e., AAA, CAA, and AAU) were predicted for 4 species. The nexus stems include up to 8 GC pairs, providing highly stable structures. In 9 species, the nexus sequence starts with a GGC motif, as observed for many Type II-A. However, the nexus stem exhibits a remarkable variability in length (3 to 18 bp) and sequence (Supplementary Table S4 and Supplementary Figure S6).

From a general perspective, the predicted DR/tracrRNA secondary structures from mollicutes show ΔG free energies ranging from −47.80 to −73.20 kcal/mol, which is comparable or even lower than the values predicted from bacteria with higher G + C genome contents (Supplementary Table S4). Genes encoding Cas9 have a slightly lower G + C% than genomes with average values of 24.2% and 27.1%, respectively. By contrast, most tracrRNAs from mollicutes show G + C% significantly higher (average value 33.5%) than corresponding genome (Wilcoxon rank sum test, *p*-value = 0.0004, Supplementary Table S4). This tendency is not observed for the non-mollicutes bacteria used in this study. This suggests that CRISPR/Cas systems from mollicutes evolved under a selective pressure to keep some G-C pairs in DR/tracrRNA for the stability of secondary structures.

### Some Spacers From Mollicutes CRISPR Arrays Target Phage and Bacterial Genes

In order to get some insights on the potential origin of the spacers present in CRISPR loci of mollicutes, all spacers were retrieved from the 17 identified CRISPR arrays and potential corresponding protospacers were searched in mollicutes (database of 224 genomes) and prokaryotic phages (database of 2480 phages) using a blastn approach. Out of the 595 spacers retrieved, 562 were unique (Supplementary Table S5). Repeated spacers were observed in CRISPR arrays of *M. apis* B31 (1 repeat), *M. palmae* J233 (8 repeats), *M. gallisepticum* S6 (1 repeat), *M. dispar* ATCC27140 (1 repeat), *M. ovipneumoniae* NM2010 (5 repeats), *M. hyosynoviae* NPL1 (1 repeat), *M. arthritidis* 158L3_1 (2 repeats), *M. mobile* 163K (2 repeats), and *Ca*. H. crinochetorum Av (2 repeats). Most spacers did not have any hits in the databases used, however, for 15 of them, highly similar sequences were retrieved. Each potential protospacer was manually checked. One spacer from the CRISPR array of *S. apis* B31 (spacer 24) had a perfect hit in a CDS of the *Spiroplasma floricola* 23-6 genome (SFLOR_RS04885). Interestingly, this CDS is part of a predicted ICE and is homologous to CDS19 described in other ICE from various mollicutes species. A total of 5, 1 and 2 spacers targeting phage and prophage sequences were identified in the CRISPR arrays of *M. cynos* C142, *M. canis* PG14 and *M. arthritidis* 158L3_1, respectively. A detailed analysis was conducted on these species.

**FIGURE 5:**
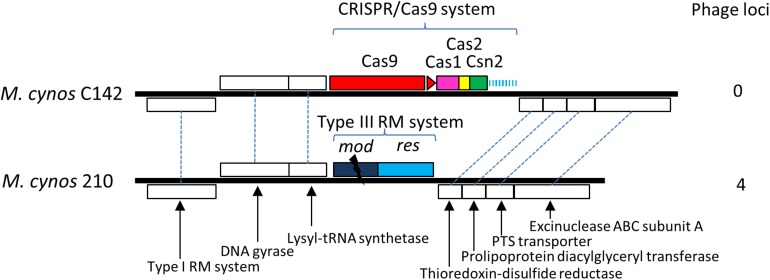
Comparative genomics of CRISPR locus in two strains of *M. cynos*. Syntenic CDS shared by strains C142 (MCYN0853 – MCYN0843) and 210 (MCYNC210_0918 – MCYNC210_0909) are represented by white boxes connected by dashed lines. The CRISPR/Cas9 locus found in C142 is replaced by genes encoding a Type III restriction/modification system in strain 210. The disrupted mod gene of this system is indicated by a black flash. Number of genome regions encoding prophage parts are indicated.

Comparison of the genomes of *M. cynos* C142 and 210 strains showed that the complete CRISPR/Cas locus identified in C142 was replaced by genes encoding a Type III restriction/modification system in strain 210 (Figure 5). Moreover, 4 genome regions encompassing a total of nearly 50 kbp and showing high similarity with a MAV1 prophage locus of *M. arthritidis* 158L3_1 genome (Supplementary Figure S7), were identified in *M. cynos* 210 but were missing in strain C142. Out of the 18 spacers predicted in the CRISPR array of C142, five of them (spacers 1, 2, 3, 9 and 18) presented 100% identical sequences with some genes encoding phage proteins in strain 210. A total of 10 CDS spread in the four phage regions of the strain 210 genome were targeted by the CRISPR/Cas system of strain C142 and seven of them included two different protospacers. In *M. canis*, comparison between strains PG14 and LV showed an inactive CRISPR/Cas system (mutation in *cas9* and IS integrated in CRISPR array) in LV strain. As observed in *M. cynos* example, two regions of *M. canis* strain LV genome showed high similarity with phage MAV1 which were not found in strain G14. Blast analysis of spacers from *M. canis* PG14 showed that spacer 6 was matching (96.7%) in the two regions of *M. canis* LV corresponding to the putative prophage MAV1. All these results suggest that these CRISPR/cas systems identified in two mycoplasmas infecting dogs are highly dynamic and probably fully active to prevent those minimal bacteria against phage infections.

In the CRISPR array identified in *M. arthritidis* 158L3_1 genome, 2 spacers (spacer 14 and 26) out of 33 matched with potential protospacers identified in the genome of the bacteriophage MAV1 but also in the own genome of the mycoplasma. Indeed, a complete prophage MAV1 was found integrated in the bacterial genome (15 CDS from position 706929 to 721810) of strain 158L3-1. In addition, a protospacer with sequence identical to spacer 13 was identified in a *M. arthritidis* 158L3_1 gene encoding a phenylalanine-tRNA ligase subunit beta (MARTH_RS00670). Remarkably, the gene encoding Cas9 in this mycoplasma was predicted to be disrupted by frameshift mutation generating an UAG STOP codon at the amino acid 394, providing an explanation for tolerating self targeting spacers.

Other cases of self targeting spacers were identified in other mycoplasmas. Two *M. dispar* ATCC27140 spacers (spacer 13 and 19) matched its own genome and the genome of other mollicutes species in the 23S ribosomal RNA gene. Three others spacers (*M. dispar* ATCC27140 spacer 7, *A. palmae* J233 spacer 13, and *M. mobile* 163K spacer 56) were shown to match on two protein encoding genes (respectively MDIS_RS03005: ABC-transporter ATP binding protein, BN854_RS00695: FAD-binding protein and MMOB_RS02220: glycosyltransferase family 2 protein). Surprisingly, while *cas9* gene was predicted disrupted in *A. palmae* J233, CRISPR/cas systems in *M. dispar* ATCC27140 and *M. mobile* 163K were predicted to be complete and functional.

In order to identify PAM sequences recognized by Cas9 of mollicutes, upstream and downstream sequences of the identified protospacers were retrieved and compared. We did not identify any clear consensus from this set of sequences. A more focused analysis was undertaken using the CRISPR array of *M. cynos* C142 for which five spacers with hits had been found. However, even in this specific case the protospacer adjacent sequences remained highly variable and no clear consensus could be defined.

## Discussion

### Distribution and Origin of CRISPR/Cas Systems in Mollicutes

CRISPR/Cas systems were found in 21/52 mollicutes species (40%) included in this study, which is a significant proportion considering the fact that some systems might be detected in seemingly CRISPR-lacking species as long as more genomes become available. CRISPR/Cas systems were detected in the genomes of a variety of species belonging to most major phylogenetic branches and infecting various hosts including pigs, ruminants, dogs, birds, fish, and arthropods. However, CRISPR/Cas systems remain undetected in some groups of species, belonging to the same phylogenetic branch or associated with the same host. This is the case of human pathogenic mollicutes infecting pulmonary and urogenital tracts (153 genomes available). This suggests that despite being found in ecological niches shared with a wide diversity of bacteria having CRISPR/Cas systems (i.e., lactobacilli, streptococci, *Acinetobacter*, *Pseudomonas*, *E. coli* …), these mollicutes have not acquired CRISPR/Cas systems from non-mollicutes bacteria. CRISPR/Cas systems also remained undetected in the ruminant pathogens belonging to the Mycoides cluster and the *M. agalactiae/M. bovis* group (74 genomes available) they were, however, found in other ruminant mycoplasmas, namely *M. ovipneumoniae*, *M. dispar* and *M. arginini*. Interestingly, *M. arginini* is frequently co-isolated with other ruminant mycoplasmas and especially *M. bovis* (F. Tardy, personal communication). Therefore, it appears rather surprising that CRISPR/Cas systems do not seem to have spread among all ruminant mycoplasmas. The lack of CRISPR/Cas systems in some specific species might be explained by an ecological niche where viruses are poorly abundant. This might be the case for some intracellular haemotrophic mollicutes (*i.e., Mycoplasma haemocanis, Mycoplasma haemofelis, Mycoplasma suis*, not shown) as it has been observed in other obligate intracellular bacteria such as *Chlamydiae* (Burstein et al., 2016). However, this possible explanation cannot be invoked for all mollicutes groups lacking CRISPR/Cas systems, some of them showing many traces of phage attacks (see below).

All CRISPR/Cas systems identified in mollicutes were classified as Type II. This finding is in accordance with previous studies showing this type is mainly found in commensal and parasitic species (Bernheim et al., 2019). Cas9 phylogeny suggested most CRISPR/Cas systems of mollicutes can be classified as Type II-A, a subtype mostly found in Firmicutes which is in accordance with the common origin of both bacterial phyla (Segata et al., 2013). In mycoplasmas, Cas9 phylogeny suggests that all CRISPR/Cas systems derive from a common ancestor. However, as CRISPR/Cas systems are prone to circulate via HGT (Jiang et al., 2013), it remains highly speculative to predict such a system was present in mycoplasma last common ancestor. In accordance with the idea that CRISPR/Cas systems might have occasionally spread among mycoplasmas after speciation, Cas9 phylogeny within the mycoplasma branch only partially reflects species phylogeny. For instance, Cas9 from *M. lipofaciens* ATCC35015 clustered with Cas9 from remote species *M. hyosynoviae* NPL1 and *M. spumans* ATCC19256. Moreover, Cas9 from the bird pathogens *M. gallisepticum* S6 and *M. synoviae* 53 clustered in a highly supported branch despite these species being phylogenetically remote. This suggests possible exchange through HGT, as it has been shown for other parts of the genome (Vasconcelos et al., 2005; Sirand-Pugnet et al., 2007; Arfi et al., 2016). While our data suggested possible HGT of CRISPR/Cas systems among mycoplasmas, no CRISPR/Cas system was found in associated plasmids, ICE or phages. Therefore, molecular and cellular mechanisms involved in spreading CRISPR/Cas systems among mollicutes remain elusive. We found no evidence of HGT with non-mollicutes prokaryotes. This might result from the particular genetic code used by most mollicutes. For example, with the exception case of *A. palmae* J233 that uses universal genetic code, genes encoding Cas9 in other mollicutes contain up to 18 UGA codons (average 9 UGA) that encode tryptophan but are interpreted as stop codon in other bacteria. Moreover, we observed that Cas9 encoding genes have evolved toward a low-GC content, in accordance with the general bias of mollicutes genomes. These genetic features of mollicutes CRISPR/Cas systems suggest they have been present in this class of bacteria for enough time to adopt the typical genetic particularities of their genomes. Moreover, presence of many UGA codons in *cas* genes most probably limits the possibility of transfer or at least persistence in other bacteria. In spiroplasmas, a phylogenetic tree inferred from Cas9 proteins and comparisons of CRISPR locus organization and Cas9 nuclease domains showed that both Type II-A and Type II-C systems were present in this large group of mollicutes found in arthropods. Furthermore, clear inconsistencies between Cas9 and species phylogenies indicated a complex history of CRISPR/Cas systems in spiroplasmas, with several origins and probable HGT. Complementary analyses using additional genome sequences of spiroplasmas (extended search, August 2019, 28 genomes available) confirmed the presence of CRISPR/Cas systems from one of the two subtypes in 10 genomes, with Type II-A predicted in *S. litorale, S. helicoides, Spiroplasma turonicum, Spiroplasma gladiatoris, Spiroplasma taiwanense* and *Spiroplasma alleghenense* and Type II-C in *S. apis, S. syrphidicola, Spiroplasma clarkii* and *Spiroplasma eriocheiris* (this study and C.H. Kuo, personal communication).

### Adaptation of Cas9 and tracrRNA in Mollicutes

CRISPR/Cas systems of mollicutes appear as typical Type II systems with the same set of *cas* genes including *cas1, cas2, cas9* and *csn2*, the latest lacking in the Type II-C systems found in *S. apis* B31, *S. syrphidicola* EA1 and *Ca*. H. crinochetorum Av. Fine analysis of main players Cas9 and tracrRNA showed some particularities of mollicutes CRISPR/Cas systems. While mollicutes are known to be minute-sized bacteria with minimal and highly compact genomes, Cas9 proteins from mollicutes include all domains described in structurally and functionally characterized homologs with a global length even slightly larger than the closely related SaCas9 from *S. aureus*. Main variations within mollicutes Cas9 and compared to SaCas9 occurred in the WED and PI domains that are involved in PAM duplex recognition. This suggests that PAM specificity is probably different among mollicutes systems and different from the NNGRRT PAM sequence recognized by SaCas9 (Ran et al., 2015). The PAM specificity of mollicutes Cas9 could not be predicted from the few protospacers identified. Further studies including *in vivo* or *in vitro* assays will be necessary to characterize the functionality of mollicutes Cas9 and their PAM specificities. The global divergence observed among tracrRNA groups and phylogeny of the corresponding bacteria has been interpreted as a resulting from a combination of HGT and convergent evolution (Faure et al., 2019). In the case of mollicutes, the remarkable sequence diversity of tracrRNA is consistent with the fast evolution of these bacteria that has been concluded from many studies and the particularly long branches observed in phylogenetic trees (Ciccarelli et al., 2006; Wu et al., 2009). Analyses of predicted tracrRNA/DR hybrid structures showed many typical features of Type-IIA RNA components in other bacteria. Notable is the wide diversity of length of different stem loops and the absence of bulge in some species whereas it has been shown to be required in other systems. By contrast with Cas9 encoding gene, tracrRNA from mollicutes have significantly higher GC% than the corresponding genome. This suggests that evolutionary constraints to keep RNA hybrid stability (i.e., dG that is not lower than for non-mollicutes systems) are strong enough to prevent decrease of GC%. This trait was also observed for some other highly structured cellular RNAs of mollicutes, including rRNAs (Sirand-Pugnet et al., 2007).

### Impact of CRISPR/Cas Systems in the Control of Phages and Mobile Genetic Elements

One of the main questions about CRISPR/Cas systems in the bacterial world is their efficacy in controlling invading DNAs, and the evolutionary benefits and costs of such systems. This question is particularly interesting for mollicutes which evolution is mainly marked by significant genome down-sizing, a global streamlining of the basic functions for cell life and general adoption of a host-dependent lifestyle. To the best of our knowledge, mollicutes are the bacteria with the smallest genome where CRISPR/Cas systems have been found. Some endosymbiotic bacteria from the *Proteobacteria* (i.e., *Buchnera, Ca*. Tremblaya, *Ca*. Hodgkinia, and *Ca*. Carsonella…) and *Bacteroidetes* (i.e., *Ca*. Sulcia and *Ca*. Uzinura) phyla have genomes even smaller than mollicutes. Besides being even more dependent on their host metabolism, such bacteria seems to be more protected against potential invasive DNA, as suggested by the lack of prophages, mobile elements and also by the absence of defense systems such as CRISPR/Cas and RM in most of them (Touchon et al., 2016). In mollicutes, most genomes have traces of mobile elements including prophages, IS, ICE, and plasmids, suggesting they are still subject to MGE-carried fluxes of genetic information (Supplementary Material SM2 and Supplementary Table S6). Some genomes as *M. mycoides* subsp. *mycoides* PG1 (IS represent 13% of the genome) (Westberg et al., 2004), *Ca*. phytoplasma asteris Onion Yellows strain (8.6% are potential mobile units) (Bai et al., 2006), *M. agalactiae* 5632 (ICE and IS represents >10% of the genome) (Nouvel et al., 2010) are particularly illustrative of the significant impact of these invading DNAs in reshaping mollicutes genomes. Substantial viral invasion in some specific phyla of spiroplasmas was concluded from the genome analysis of *S. citri* GII-3 (>20% genome are prophage remnants) (Carle et al., 2010) and related species *S. kunkelii* (Bai and Hogenhout, 2002), *S. melliferum* (Lo et al., 2013), *S. poulsonii* (Paredes et al., 2015) while typical plectroviral sequences were not found in other spiroplasma phyla (Ku et al., 2013). Because of the specific genetic code used by most mollicutes, circulation of MGE is most probably favored within this group of bacteria. This has been suspected from phylogenomic studies indicating some MGE had been recently exchanged among species sharing the same host, including IS among human mollicutes from the urogenital tract (Pereyre et al., 2009), IS and ICE among ruminant mycoplasmas (Thomas et al., 2005; Citti et al., 2018) and ICE-related elements in some spiroplasmas (Lo et al., 2013). In parallel to this evolutionary trait, highly variable repertoires of genes encoding defense systems in mollicutes genomes suggest that most of these minimal bacteria undergo a selective pressure toward maintaining means to control invading DNAs. However, we did not find a clear correlation in the distribution of CRISPR/Cas systems and mobile elements.

A global survey indicated that nearly half of the bacterial genomes contained at least one prophage but that prophages are rare in small bacterial genomes (Touchon et al., 2016). Accordingly, our survey indicated traces of prophages in about 10% of mollicutes genomes. Surprisingly, lysogen bacteria are statistically slightly more likely to have CRISPR/Cas systems (52%) than non-lysogens (43%). More precisely, Type II CRISPR/Cas systems are as frequent in lysogen and non-lysogen bacteria, suggesting these systems might not be as efficient as previously thought in immune defense against phages. Despite the mollicutes genomes with prophages and CRISPR/Cas being currently too rare to get a real picture, this tendency might be also observed in mollicutes, with 4 out of 5 lysogen species having a complete or incomplete CRISPR/Cas system. Therefore, as more genomes become available, it will be interesting to re-evaluate the tendency of CRISPR/Cas systems to occur in mollicutes phyla where traces of phage attacks can be evidenced.

### Persistence of Degraded CRISPR/Cas System in Mollicutes

Inactivation and more or less advanced degradation of CRISPR/Cas systems have been predicted for at least 8 out of 21 CRISPR/Cas systems of mollicutes (38%). Overall, our data suggest that CRISPR/Cas systems were frequently acquired during the evolution of main mollicutes groups but also frequently inactivated and possibly completely lost in several phyla. This assumption is in accordance with other studies proposing that fast evolution of CRISPR loci including acquisition by HGT, expansion of CRISPR arrays, degradation and complete loss might be driven by opposing positive and negative selection for immunity keeping CRISPR/Cas systems in a continuous state of flux (Jiang et al., 2013). CRISPR/Cas systems would be acquired in environments where phages are a major source of mortality while being quickly inactivated and lost when the benefit of the protection system does not counterbalance the cost of expressing constantly the Cas proteins and reducing the possibility to acquire new genetic information by HGT. This would make sense in the context of minimal bacteria where most cellular energy is likely devoted to translation, and where HGT has been shown to play a significant evolutionary role at least for some groups.

In addition, it has been proposed that accidental incorporation of self-targeting spacers might be as frequent as 18% in CRISPR-containing prokaryotes (Stern et al., 2010). This could incur an autoimmune fitness cost, and therefore help to explain the abundance of degraded CRISPR/Cas systems across prokaryotes. Self-targeting spacers were found in some CRISPR arrays in several mollicutes species (i.e., *M. dispar* ATCC27140, *M. mobile* 163K, *M. gallisepticum* S6 and *A. palmae* J233) suggesting avoidance of self-targeting is not perfect in these species, as in many other bacteria. Non-functional CRISPR/cas systems were predicted for *A. palmae* J233 and *M. arthritidis* 158L3_1 where mutations in *Cas9* were detected, providing an explanation for the survival of the bacteria. However, for *M. dispar* ATCC27140, *M. gallisepticum* S6 and *M. mobile* 163K, the CRISPR/Cas systems were predicted to be functional. Therefore, our current hypothesis for these cases is that PAM sequences might be mutated avoiding target cleavage. In minimal bacteria as mollicutes, persistence of non-functional CRISPR/Cas systems remains somewhat puzzling as genome streamlining appears to be a strong evolutionary force. Interestingly, in the best documented example currently available for studying the evolution of CRISPR locus, *M. gallisepticum*, fast degradation of the locus was observed in some isolates after host shift from poultry to House Finches (Delaney et al., 2012; Tulman et al., 2012). As a growing number of genomes becomes available for different species, we will probably soon learn more on the acquisition and persistence of CRISPR/Cas systems of mollicutes and their biological importance in the fast evolution of these minimal bacteria.

## Data Availability Statement

Publicly available datasets were analyzed in this study. Genome data can be found at GenBank, under the following accession numbers: GCA_000731685.1, NC_007633.1, NC_014751.1, NC_005364.2, NC_0154 31.1, NZ_ANFU00000000.1, GCA_000380285.1, NC_0210 83.1, NC_006055.1, NC_022998, NZ_CP017015, NC_02184 6.1, NZ_CP012357.1, NC_021284.1, NZ_CP002082.1, NZ_AORH00000000.1, NC_014760.1, NC_009497.1, NC_0145 52.1, NZ_JMKY01000006.1, NZ_JHZE00000000.1,NC_014014.1, NC_007294, NC_019949, NZ_CP014281.1, NC_002771.1, NC_006360.1, NZ_CP007229, NZ_JAKV01000001.1, NC_0144 48.1, GCF_000485555.1_ASM48555v1, JFKL01000035.1, GCA_000367765.1, NZ_AP014657,JHVF01000003, NC_01351 1.1, NC_011025, NC_006908, CP006932.1, GCA_000227355.2, NC_004432.1, NC_002162.1, NC_000912.1, NC_000908.2, NC_023030, NC_011047.1, NC_010544.1, NC_005303.2, NC_022538, NC_010163.1, NC_022549.1, NC_008532.1, NC_008532.1, NC_006368.1, NC_002737.2, NC_013198.1, NC_003454.1, AECD01000042.1, NC_012781.1, NC_002163.1, NC_003116.1, NC_002663.1, GCA_000185485.1, and NC_008601.1.

## Author Contributions

TI and PS-P designed the study. TI, IT, VT, CG, AM, CW, PT, and PS-P performed the analyses and analyzed the data. TI, IT, CW, JB, AB, PT, and PS-P wrote and improved the manuscript.

## Conflict of Interest

The authors declare that the research was conducted in the absence of any commercial or financial relationships that could be construed as a potential conflict of interest.

## Footnotes

1 https://weblogo.berkeley.edu/
