# Supplementary material for: Multiple Origins and Specific Evolution of CRISPR/Cas9 Systems in Minimal Bacteria (Mollicutes)
Source: Front Microbiol. 2019 Nov 21;10:2701. doi: 10.3389/fmicb.2019.02701 (PMC6882279; doi:10.3389/fmicb.2019.02701)
Supplement: Supplementary file 1 [file Data_Sheet_1.docx]

**Supplementary Material**

**SM1.** **Key amino acids involved in gRNA and target recognition are conserved in Cas9 proteins of mollicutes and *S. aureus***

Comparison of various Cas9 proteins and crystal structures of Cas 9 of *S. pyogenes* and *S. aureus* have evidenced a global organisation with two endonuclease domains. The HNH domain cleaves the DNA strand complementary to the guide RNA whereas the RuvC domain cleaves the non-complementary strand (Nishimasu et al., 2015). Structural studies have shown that SpCas9 and saCas9 bound to single guide RNA adopt a flexible bilobed structure consisting in a recognition lobe (REC) and a nuclease lobe (NUC) with a channel wrapping the RNA-DNA duplex (Jinek et al., 2014; Nishimasu et al., 2014, 2015). The C-terminal part of the Cas9 proteins includes the PAM-interacting domain (PI) which interaction with the PAM sequence located on the non-target strand of DNA is required for the initiation of the cleavage process. While they both exhibit the same general domain organisation, SpCas9 and SaCas9 share only 17% similarity and have significantly different sizes, 1368 aa and 1053 aa, respectively. They also have different PAM specificity, SpCas9 regognizing NGG and SaCas9, NNGRRT (R represents A or G). Finally, fine structure comparison revealed some noticeable differences including interacting regions of REC lobe with sgRNA, of PI domain with the PAM sequence and a highly divergent WEDGE (WED domain).

Based on multiple alignment of the Cas9 proteins and SaCas9, conservation of key amino acids was manually inspected (Figure S4).

In the following description, numbering of the amino acids refers to SaCas9 (Nishimasu et al., 2015). Among the SaCas9 amino acids bound with gRNA and target DNA, most conserved ones (identical or similar) in Cas9 from mollicutes were involved at three levels:

*Recognition of the heteroduplex formed by pairing of the gRNA with the target DNA.* Most conserved residues include Tyr651 (RuvC domain), Trp229, Gly235, Thr392 and Arg245 (REC lobe) that were shown to interact with the target DNA strand. Many amino acids known to be involved in recognition of the paired gRNA were also well conserved. Those are part of the Bridge helix (Arg48, Arg51, Arg55, Arg59, Arg60) and the REC lobe (Arg209, Arg116, Gly117, Arg165). Some SaCas9 residues from the REC lobe interacting with the gRNA nucleotides paired with the non-PAM extremity of the target DNA strand were replaced by similar residues in most mollicutes, including Thr238 (Ser in most mollicutes) and Tyr256 (Phe in most mollicutes).

*Interaction with the nexus stem-loop formed by self folding of the tracRNA part of the gRNA.* Conserved amino acids includes Arg47, Arg55, Arg54, Arg58 (Bridge helix) and Ser219, Gly216 and Arg209 (REC lobe). The Phosphate lock loop amino acids Asn780 and Leu783 that are involved in the recognition of the first Adenine nucleotide (A55) downstream of the lower stem of the repeat-antirepeat duplex are highly conserved within Cas9 of mollicutes.

*Binding to the Repeat-antirepeat duplex.* Some of the SaCas9 amino acids involved were also conserved in mollicutes Cas9, including Lys114, Gly162, Arg165 (REC lobe) and Lys878, Lys881 (WED domain). However, some other residues involved in this interaction were only poorly conserved, especially those bound to the gRNA at the bottom of the lower stem-loop.

**SM2. Distribution of CRISPR systems in mollicutes is not correlated with presence of MGE and other defense systems**

In order to investigate the global impact of CRISPR systems against phages and other mobile genetic elements (MGE) during the evolution of mollicute genomes, repertoires of MGE including prophages, IS, ICE, plasmids were predicted in the 52 genomes set. In addition, non-CRISPR defense systems against invading DNAs were also listed, namely, RM and Abi systems (Table S6).

Among MGE, IS were the most frequent, with 86% of the selected 52 genomes harboring elements of one or several IS families. IS were predicted in all groups of mollicutes and in most genomes, with the exception of *Me. florum* L1, *S. taiwanense* CT-1, *M. crocodyli* MP145, *M. mobile* 163K, *M. genitalium* G37 and *M. pneumoniae* M129. Complete and potentially degraded ICEs were found in 35 % of the selected genomes, widespread in the Spiroplasma and Hominis phylogenetic groups while not predicted in the mollicutes from the Pneumoniae and Acholeplasma/Phytoplasma groups. Large Potential Mobile Units (PMU) have only been described in phytoplasmas. Plasmids and prophages were more rarely detected, in 11 % and 13% of the selected genomes, respectively. With the exception of a putative prophage remnant in *U. parvum* serovar 3 ATCC 700970, these MGE were not observed in the genomes belonging to the Pneumoniae group. Regarding defense systems against invading DNAs, the most massively prevalent ones are the RM systems. They were predicted in 96% of the selected genomes, with the notable exception of *S. taiwense* and *S. mirum*. Type I and II RM systems were more frequently identified than Type III and Type IV which were only found in 4 genomes. Besides CRISPR systems, abi systems were also detected in mollicutes, but only in 5 genomes distributed in the Hominis group and in the mycoides cluster.

Statistical analyses conducted to evaluate whether some correlation or anti-correlation could be found between the distribution of MGE and defense systems only indicated correlations for the occurrences of IS and RM systems (kendall’s ρ = 0.258) and IS and prophages (kendall’s ρ = 0.212). No statistically significant correlation could be obtained between CRISPR systems and other MGE or defense systems. However, out off five mollicute genomes where potentially complete prophages were detected, three had uncomplete CRISPR systems (*M. arginini* HAZ145_1, *M. spumans* ATCC19526, *M. arthritidis* 158L3-1) and one had a potentially complete CRISPR (*M. lipofaciens* ATCC 35015). For this last case, examination of the spacers present in the CRISPR array showed no similarity with the prophage sequence.
