# Supplementary material for: Multiple Origins and Specific Evolution of CRISPR/Cas9 Systems in Minimal Bacteria (Mollicutes)
Source: Front Microbiol. 2019 Nov 21;10:2701. doi: 10.3389/fmicb.2019.02701 (PMC6882279; doi:10.3389/fmicb.2019.02701)
Supplement: Supplementary file 2 [file Presentation_1.pdf]

>Acholeplasma_palmae_FUSION gi|1181299883|ref|WP_084600768.1|:1-878 type II CRISPR RNA-guided endonuclease Cas9 [Acholeplasma palmae]FUSION
-----M-----------------------------FM---------LQWCKVLLLITG--GY-MK--
KILGLDVGVTSVGWAIIDEEG------N------------------IVKTGVR--LFEEASAK-----
NNLDRRTFRGQRRLIRRRNQRIDDMRSLLIKNL----LI-D-----------------N--------------
----------------Q---F----------K---------------VL-------------------
DNPYQLREKGLK--E--KLNHEELATVLLHYAKRRGSSLE-----------------------------V--
VE----EE------G--------------------I-------------------------------------
---KKE-TS-T----
KSILNENAKELKE---K--
QYVVN---------------------------------VQ-LNR-----------------LKK--------
EGK--------LRGN-------------------------------------INN------------------
--FKTKDY---------------------

AKEIIT----LLDNQN--------L----D-----SELK---------KEIIEIIQRRRHFSEGPG------
NF--N----SPTPY--GRY-------------------
REVTGTLKEKIIKDIHANYRKQYLNSNFSIVFDDIEYQVF--------K--NGR--I-V--------
NKEKYDLIDLMRGKCSLYPDQPRSPKMAFSAE--IFNLLNDLNNLKILN--R------E------N----N-
KITKEEKITII-N-HV-REK-----------------------GSITV----QQ-----LLK-I---------
-----L-N-------A-----QQ-E--EIKGF--RID-K---N---EKPIITEFKG-----------------
----
------------------------------------YKKILK------VY-KEL-------------------
----------------DI-----T------------I---DSDEILDRIIEI----LTQTL-VEEER--
ILSLK------K---------E--KINE-RL----IEPL-S-L---------LTG------IK----EYHSL-
--SLKA-------------------------------------IYQL--------------NKEM--------
--LE-ES----------L--N--
-QQEIIT-N--------WG--IK--N-------------------DDT---------
IKELVLDESLILSPVAKRAHREALKLVNQLIKEEG---------------------
NFSKIVIETTRSKNSLD-EIKEI----KELQKNN-ENNRRQAEEII--GD----RA---------K-----
EL---VNSSN-I--LK-LRLYNEQNGK-------CAYTG------E-----ALSIDAL-
LNDPNIYEIDHIIPISIS-FDD------SYANKVLVTRSANQKKGNKTPFGYF-SKA--DL----VANSK-
INSWNMF--------K-------EI-VLL---N-------------------------------------K-
NYS-IRKK------------QNLLFEE---DITKHSVVSDFINRNLVD----TSYAIRSLMTTLKNYFKS---
----------N-----------------------HVDTTIMTI-KGKQTNYFRKTGMIAWSRKHPKRENP---
----------------------------F--R-
KDRN-K-YIHHAIDA-------LIIAGLSNRKYITK--L-Y-NL-----------------------------
---S-------QEI----------DIETGEL----
I-------------E--------------------YI---------------DASEDSK--------------
L-IQY--L----L-------KVGEV--Q-EED------------------------V----KFS-----
WKID-------SKPNRSMMNQT--IYSS-RIL--G------G----------ESL------------------
---LL-----------------------------SKI-D-------IYNSKKE--DF---IKKN-Y---Y---
---------------YNKENVLMY--QND-------------------P--TTF-E-----------------
---------------IVI-KAFE--QYKH---------------------E----------EYPFKVYKE---
--NY---
---G-YIR------K------------Y------------S-KN--------NNGA-----------------
---PVINMKYL---G--D-AI---K--SEVKDL-SDRYNVDVTKKMVFQSSIKPFRVDVYY--NG--------
IQYK---
---------VVT--VT--YKDVV---K--KDGKYR--------I---------INYT----------------
------EK-----------IKK--KE----------------I-DKKYIFRFSLFNNSIIT------------
--ID----N-SE--FY--RF-K-------------GI-V-DL-EN----------KF----------------
----EVT-T--AN-------------------------YRPVKQNKNNKE-ELDRIRRVI-NK----------
---K--IKNITK------------Y-N----VSNTGKI--------------AKV--E-K--E-------E--
-LKLVF
>Mycoplasma_dispar gi|765307734|ref|WP_044635168.1|:1-1263 type II CRISPR RNA-guided endonuclease Cas9 [Mycoplasma dispar]
-----M--GK-KH--
ITIGFDLGIASVGWAIIESDTS-----K------------------ILNWGSR--LFEERKT-------

AQKRRGHRSTRRNIRRKAYRNQKFMNLILKYK----ELFG-----------------LE--NTSQ--
ISRIDKKDVENYKKIEEKFTEI---Y----------R----------N-CANKY-------------------
PNILDLKIKALN--S--KIEKLELVWILHDYLENRGFFYD-----------------------------I--
EQ---NSE------E--------------------E-------------------------------------
---KKK--NK---KFAS----
--KYKGS--EFPSI-
-----------------------------------LL-DRF-----------------FKK--------NGF----
----FNSY-----GLV------------------------S--EYGYN-----------------------
---------------------------------------FSNLHW---------------------------
----
--RNEISK---
-LFEIQE--------I----D-----YEFG---------KKFLDIFSSVRDYAKGPG------SK--N----
SISKY--GVF-------------------EID-------------------------------------E----
-------TGK--V-V----------EYENIWDKTIGKCSFFVQESRVSSNYPSYE--IFNLLNQLINLSSEL-
-K------L--ENGKN----W-QLKTKDRDFLL-D-KL-LEAR----K----------N-K--KNKDASI---
-EDDIIKIILA-D--------------F--GLEKTDIT-----ND-D--SIQGR--DII-K---A---KP--
TTKLET---
---TNKLLK------
II-YSH-----------------------------------SSN---AKQVKS-----NNLV---
EFLPFLDKICAI----VDHDK-A-REK--NEVLK------KL--EEKGIFSKFQIIP-EK----QEKF-L-
DSL---F-KEKLN------FK----KIGNL---SLKA-----------------------------------
-IHFF--------------LPKM--
----------IS-QN----------K--N---SEFLKWND--------LE--TR--QTWEEEKNK--
IKKVNKK--SKY---------LNPRIF-EDEIISPGTKNTFEQAILVLNQIIKKYSK-D-------------
---YQIDAIIIESPRTKNDKK-TTYKI----NKAIKES-KEKD---KKLF--EA----LN----LKE--
EGYTF-EQLKSKSKSL-F--DK-LRLYYQQEKL-------DLYDL------DSSDEGEIKINDL-
IEKSQNYEIDHIIPYSMS-YDN------SQANKILTLRAKNGEKRKEIASKYA-RKK--G-----------
EEYFKKY--------L-------EK-VKELFID-----------SIKSKNNTDYVDL-------------
DKDSS-KKKY------------RLLTL-E---DFDQ--YHAEFIARNLND----TRYSTKLFYHALRDHFQN-
------------NE------HFEYLDEKSQ------IHKVKVATI-KGHVTRYFRGKT-------------
GY-----S-----KDEIFENNAKEDESTH-KKV--
-IK-KRRE-N-NEHHAVDA-------AIVAIIGNE-N--RQ-L-ANLLTISDN----SFEN-YD---------
E--D-------HKL----------
NVRTGEI----V-------------R-------------------KPK----FE------------IEK---
-----------FAKID--E----LKNKIL-EQCTIA-KN-EIP------------------------I----
KFS-----RKLR-------TILNCQISNEN--LCGF-KFD--E------N-------Q-NKYF----------
-----------KI-----------------------------NKI-N-------LLNSTNE---E---LEKY-
F---INPF--------G----TEESKYKVLMA--ESH-------------------K--VEF-E---------
-----------------------RLK-EIFF--KYKE---------------------K----------
GDAFAKYID--
-------DL------KKKEP------ELIDEIDAAKAIGKILYY-KLEPLNQQ-TFY---GEPKIVKK-----
---------------YYKNIRII---K---------YDSIPIQFKMLSK----
HDGGKSYKDDLRSLYSLVYKVYEK----GK--ETYK-------------------------------------
---SIP--VN--SALTKFG-S--NN--ND------
--LLDENNYNS-QNLL----------------------LY-----------KNDFTKP---------------
-I-PTGCKPIIAIKKGVILK-KKNYEIQDDFTETEE----N-GR--YF--FI-S-------------GI-S-
KECGK----------NI--------------------DTR-F--TLRFIEISK--------------------
-----------DTVASKRVT-DK-------------I--FKHYDL------------I-H----LDELGNE--
------------YPI--K-I--KEHTEEEKK---LCTIK
>Spiroplasma_apis gi|753856968|ref|WP_041612583.1|:1-1180 type II CRISPR RNA-guided endonuclease Cas9 [Spiroplasma apis]
-----MSIPKPALFFIFKTNLYKFDKIFLKVKKDEK-------------------------M-KK--
YILGLDLGISSVGWAVTGYDDENAIEPW------------------LDDFGVR--
LFEVPENPKDKTSLAAERRGFRSSRRLKRRKKARIKLLKKILIDSD----II-N-----------------E-
----NL--YE------------------KTFNFSISSLKPTNKIV----------Y-DEKKY-----------
--------FNPYVIRKKGLK--E--KLSSEELTIALVHIAKNRGFENL-------------------------
----F--DP---EKG------E--------------------D-----------------------------
---KSELKD--D--

```
-AE----------------------------------------------------------------------
-----------------------INEKEDYANSIIIARKLI-GYDIATKTCKKTIAEAFIDEESS--------
TRIEKNKNLLNVRNK-----D--------------------------S--QYRFL------------------
----------------------------------------------FPRAAY---------------------
-------------------------------------------------------------------------
-----------------------------------------------------------------------
KYELEL----ILNKQAE-YYP-Q-L----N-----NEII---------DKIMDTILRQRFFEDGFG------
PK--N----ISRAENIELIKQKLSNIKDKNNHYIALRDSLR--------------------------------
-----------------A-----Y----------KLYKPFTELVGNCTFFPNEKRYIKSSIIFD--
LYQMAVEVSKFSSFL--E------N------K----Q--TIK----DFH-N-KL-FNLALTDEK---------
-F-I--SNNKKQI----ES-----VLKTD--------------F-G-------V-----KS-E--DLKNL--
KALDN---A---KKTDLFGF-----------------------------------------------------
-------------------------------------------------------------------------
--TKRFIK------VF-GME----------------------------------------KLKELNI-----
D--N---LDSNIIDDLGDI----LNKNI-TPELR--KSNIK------KW--ASD-NNV--TIND-EN----
INQL-LLT---------PSK------VT----TTSNL---CKKA-----------------------------
--------MLMV--------------IKHF-------------------------------------------
-----------------LE-GE----------I--AGVYQDE-----------------LK--AKAN--E---
--YTDLNI--KKF---------LQPIVD-GDLVRNPVVFRAINEARKVLKALFRKYN----------------
-----DFEKINVETSRELGKSGEVRNEL----NKKNLDS-RAKNEVIKSEL--EK----IG------------
---IV---ANTTS-I--LK-YKLWLAQDKK-------CLYSL------R-----DIKIEQL-N--
SHELEVDHILPISKF-PDD------SFDNKVLVYTTENQLKKNRTPLEYF-NAE--N---------P--
DIINSY--------K-------KN-CLDLYRK-------------------------------------G-
NIT-WNKY------------ENLLI-K---SVLDI-DQNKFSSRNLVD----NSYIARYFANWLKNNLIH---
----------KY----------KKE-NK------EYKSNVLMI-KGVVTSRFRRKW-------------LR--
---F-----S---------------PWGL-----------------------------------------DI-
KVRDIT-PFHHAVDA-------IVLSQFKNQ-G--SV-DFASDLIAI--------------------------
-----------------------------------------E-----N--EFKSFKYKNISLEQYHNNV----
K-------------E--------------------I-------------------------
CSKWHDNSGYQWQFQIS--K----PIERID-D---------FINSDCQAIKMFPLVKNLDKLIDLRMPI----
ELS-----VKVD-------V--------DT--VKLTEKEL--K------R----------NFK----------
-----------NL-----------------------------NEL-S-------ILNEKD--------FKK--
-----------------EIK---VKR-PVFVK--VKH-------------------P--QEYIE---------
-----------------------TLK-NA-N--VAGD---------------------I----------
HYPFVSYKV----------------------------------------------------------------
-------N---------------------------------------------HKVSGSVTS-----S-----
---------------EKMAAKKL---L--K-D-----------------------------------------
-------------------------------------------------------------------------
------------------------------SNKDQFHID--RH--GN--------LWETNVY---------Y-
-------------GVIIDKEGINKPRWIKKIDIFKNKN----------------
ILKLRDNELILRQNDTVFY--------------KN----K-DD--NY--EYKV-------------FK-S-
KM-GAQICATKINTTYI--------------------STL-H--KN--KEIFG--------------------
------------TQNCYDSL-SN-------------I--LKEIKI------------I-T----IDILGK---
--------------------------------------A
>Fusobacterium_nucleatum tr|C7XMU0|C7XMU0_FUSNV CRISPR-associated
endonuclease Cas9 OS=Fusobacterium nucleatum subsp. vincentii 3_1_36A2
GN=cas9 PE=3 SV=1
-----M-----------------------------KK-----------------------QKFSD--
YYLGFDIGTNSVGWCVTDLDYN-----V-LRF------------NKKDMWGSR--LFDEAKT-------
AAERRVQRNSRRRLKRRKWRLNLLEEIFSDEIMKIDSNFFRRLKESSLWLEDK-NSKEK--FTLFNDDN----
--------------YK---D----------Y----------D-FYKQY-------------------
PTIFHLRDELIKNPE--KKDIRLIYLALHSIFKSRGHFLF-----------------------------E--
GQ---N-------------------------------------------------------------------
-------------------------------------------------------------------------
---------LKEIKNFETLYNNLISFLEDNGINKSIDKDNIKKLEKIICDSGKGLKDKEKEFKEIFNSDKQL-
--------------------------------V------------------------A--------IFK----
----LS----------------VGSSVSLNDLFDTDEYKKEEVEKEKI-------------------------
---------------------------------------SFREQIYEDDKPIYYSILGEKIELLDIAKSFY--
```

DFMVLNNILSDSNYISEAKVKLYEEHKKDLKNLKYIIRKYNKENYDKLFKDKNENNYPAYIGLNKEKGKKEVV
EKSGLKIDDLIKVIKGYLPKPERIEEKDKTIFNEILNKIELKTILPKQRISDNGTLPYQIHEVELEK----
ILENQSK-YYD-F-L----NYEENGVSTK---------DKLLKTFKFRIPYYVGPL------NSYHK----
DKGGN--SWI-------------------VR--
------KEEGKILPWNFEQKVDIEKSAEEFIKRMTNKCTYLNGEDVIPKDSFLYS--EYIILNELNKVQVND-
-E--------------------FLNEENKRKII-D-EL-FKE----------------N------KKVSE---
-KK-----FKE-Y--------------L-L-------VNQIANRT-V--ELKGI--K------------
DSFNSNYVS--
--YIKFKD-----
-IF-GEK---
LNLDIYKEISEKSILWKCLYGDDKKIF--EKKIK-----NEY--G-----D--ILNK-DE----IKKI-N---
--------SFK------FN----TWGRL---SEKL-------------------------------------
LTGIEFINLETGECYSSVMEAL---
---------RR-TN----------Y--N---LMELLS-S--------K---FTLQESI--DN-G--N--
KEMN--EVS---------Y-RDLI-EESYVSPSLKRAILQTLKIYEEIKKITG--------------------
RVPKKVFIEMARGGDETM-KNKKIPARQEQLKKLYDSCGNDIANFSIDIK-----K-----MKSSLS--S--
YD---NNSLR-Q--KK-LYLYYLQFGK-------CMYTG------R-----EIDLDRL-
LQNNDTYDIDHIYPRSKVIKDD------SFDNLVLVLKNENAEKSNEYPVKKEIQEK--M-----------
KSFWRFL--------K-------E--------K-------------------------------------N-
FIS-DEKY------------KRLTG-K-----DDF-ELRGFMARQLVN----VRQTTKEVGKILQQIE-----
-----------------------------------PEIKIVYS-KAEIASSFREMF-------------D---
----------------------------F--I-
KVRELN-DTHHAKDA-------YLNIVAGNV-Y--NT-K-FTE------------------------------
---------------------------KPYRYLQEIKENYDV-------KKI----------YNYDIKN----
A-------------W--------------------DKE----N------------------------------
--------------------SLE---IV-KKN------------------------M----KKNTVNITR---
-----FIKEEKGQLFDLNPIKKGE-TSN--E------I-------I---------------------------
----
-----------SIKPKLYNGKEEKL-------------------N--EKY-
GYYKSLNPAYFLYVEHKEKNKRIKSFERVNLVDVNN-------------------------------------
---IKDEKSLVKYL---
----------------------------------IENKKLVEPRLIKKVY-
KRQVILINDYPYSIVALDSNKLM--------------------DFENL-------------------------
-------------------KP-----LF----------
LENKYEKILKNVIKFLEDNQGKSEENYKFIYLKKKDRYEKNETLESVKDRYNLEFNEMYDKFLEKLDSKDYKN
YINNKKYKNLLDVK--EK--FI---

----KLNLFDKAFT--LK--SF-L-------------DL-F-NR-KT----------MA--------------
------DFS-K--VG--L-
TKYLGKIQKISSNVLS--KNELYLLEESVTGLFVKKI------------------------------------
----------------KL
>Mycoplasma_hyosynoviae gi|1172314288|ref|WP_080697567.1|:1-1167 type II CRISPR RNA-guided endonuclease Cas9 [Mycoplasma hyosynoviae]
MIVEIM-----------------------------E-------------------------K-EK--
ITIGLDLGVGSVGWSIIDSN-N-----K------------------VVDLGSR--LFDEPNL-------
ALDRRAFRSRRRMIRRKAYRNNKFYKLVLKYP----DIFN-----------------VK--TKEE--LQ----
--------------QI---I----------K----------N-VNHKY-------------------
PNILDLKVKALE--N--EVTSGESIAILHDYLENRGYFYE-----------------------------I--
IE---EKE------E--------------------K-------------------------------------
---KKK--DIE----------
----KTN--QLPSI-
--------------------------------QQ-KNF-----------------FDK--------YKV----
----INDV-----F--------------------------S--KEVTQ-------------------------
---------------------------------------FSNKEW----------------------------
----
--IKEIEV---
-FSKNQS------Y-L----S-----AEFN---------EAFMNIFSACRDFALGPG------SL--H----

SPSEY--GVY-------------------NKN-----------------------------------P----
----E--TGK--VEQ---------KYNFIWDKTIGKCSFFPEENRAPKNTPSAQ--LFNLLNQLNNLRIIG-
-D------D------E----W-RLTTEDKKAIL-N-SL-LSSF--K-E---------G-K--KSSKLTL---
-KNKV---LLN-C--------------V-K------DK-----EN-P--IFSGY--DTD-K---N---
GDHKFVEIAS--
--FGVLIN----
--ALLDEN----------------------------------NANQEILNNINF-----DQMQ---
EWLPIFDKLCEI----LVYYP-DVNKR--ISQIL------EL--D--LLKNNWNMNK-EQ----LEKF-A-
NKL---S--HSFI------GA----TTHSL---SLRA-----------------------------------
-INLF--------------LPKM---
----------LN-TN----------S--N---YESCKYE-------YLHN--LN--IK----N-Q--
EQPPQKQ--TKY---------ISAAFL-EKEILPPSVKSTMRESIAIVNAIKKQYAN-K-------------
---YEIENIVIEMAREKNSSE-KKKKI----LKLQEKN-KEKKKLCDEEL--KK----HG----YSQ--E--
AI-DK---VNVHT-S--LK-IKLWIEQQHI-------DPYIG------K-----EIDFDKM-
VKDNSFTEIDHIIPYSMS-ADD------SWANKVLVLRTSNQTKGRKIPYDYF-ETN--QL--------P-
NWGWNEY--------V-------AW-CNDKILN----------GPK-------ELLP------------
KKELR-IKKF------------NNLTL-T---NFDS--NNLGFLARNLND----TRYMSKLFRDKLIEYSKA-
------------N-----------------------NNAFKVYTL-NGNITSYVRKII------------
DN-----T-----K---------D------APY---------------------------------------
-MK-KDRN-D-FSHHAYDA-------TILALISKN-A--YW-L-FKKIDDQNS-------R-YMIQT----
NSD---G-----KVYKL----------
DILTGEL----T-------------S-------------------FKN---NKS------------DNK---
-----------IFDIY--A----IANMVY-EKTK---DINRED------------------------I----
KFS-----RKQV-------VKTNGELFNKT--KYGY-KED--K------E-------NQEIIY----------
-----------KI----------------------------EKI-N-------LITEELK---K---LKDY-
F---G------------NEA-NETKVQSLLIY--QER------------------P--EQY-K---------
-----------------------MLN-NIYL--EFIAK-----------------NSK----------
QNPFITYMN---
-------EL------LEKCE-----IFGIDK-------KYVEIT--KRNKIIL-FNQE-N----KIIQ-----
---------------VVGNLKYI---S--D-ER---K---KI-NV--FL----
DEKNKCFQDSFKPFGALVYK---D----KSKKKIFK------------------------------------
--EVA--IN--AKIIKFQ-D--KK--ID------
--WYDESNYFE-DNLR----------------------KI-----------KAF--KN---------------
-I-DVNANIEQVILIGDKWI-------------N-D----D-HQ--IV--YI-N-------------GV-G-
DT-NN----------SI--------------------EYN-F--ID--KNVLI-------------------
----EKNDKS-EKKRNKLSI-GE-------------F--LKIFRP------------I-N----INNLGRI--
------------LN---------------------IKKK
>Neisseria_meningitidis WP_002235162.1 type II CRISPR RNA-guided endonuclease Cas9 [Neisseria meningitidis]
-----M----------------------------AA---------F---------KP--NP-IN--
YILGLDIGIASVGWAMVEIDED-----E-N-------------PICLIDLGVR--
VFERAEVPKTGDSLAMARRLARSVRRLTRRRAHRLLRARRLLKREG----VL-Q-----------------
AA--DFD-----------------------EN--GL----------I------------KSLP---------
----------NTPWQLRAAALD--R--KLTPLEWSAVLLHLIKHRGYLSQ----------------------
------R--KN---EGE------T--------------------A---------------------------
---DKELGA-L-
---LKGVADNAHALQT--
GDFRTPAE--------------------------------LALNKF-----------------EKE------
--SGH--------IRNQ-----R-------------------------G--DYSHT----------------
---FSRKDL-------------------

QAELIL----LFEKQKE-FGNPH-V----S-----GGLK---------EGIETLLMTQRPALSG--------
----
-----------------------------------DAVQKMLGHCTFEPAEPKAAKNTYTAE--
RFIWLTKLNNLRILE--Q------GS-----E----R-PLTDTERATLM-D-EP-YRK--------------
--------SKLTY----AQ-----ARK-L--------------L-G-------L-----EDTA--FFKGL--
RYG-K---DN-AEASTLMEMKA--

```
-------------------------------------------------------------------------
--YHAISR------AL-EKE-----------------------------------GLK---DK---K-----
SPLN---LSPELQDEIGTA----FSLFK-TDEDI--TGRLK------D------------RIQP-EI----
LEALLK-----------HIS------FD----KFVQI---SLKA-----------------------------
--------LRRI--------------VPLM-------------------------------------------
-----------------EQ-GK----------R--Y---DEACA--E--------IYGDHY--GK--------
----KNTE--EKI---------YLPPIP-ADEIRNPVVLRALSQARKVINGVVRRYG----------------
-----SPARIHIETAREVGKSFKDRKEI----EKRQEEN-RKDREKAAAKF--RE----YF------P--N--
FV-GE---PKSKD-I--LK-LRLYEQQHGK-------CLYSG------K-----EINLGRL-N-
EKGYVEIDHALPFSRT-WDD------SFNNKVLVLGSENQNKGNQTPYEYF-NGK--D---------N-
SREWQEF--------K-------AR-VET-----------------------------------------S-
RFP-RSKK------------QRILL-Q---K---F-DEDGFKERNLND----TRYVNRFLCQFVADRMRL---
----------T----------------G------KGKKRVFAS-NGQITNLLRGFW-------------G---
----------------------------L-----------------------------------------RK-
VRAE-N-DRHHALDA-------VVVACSTVA-M--QQ-K-ITRFV-----------R-YKEMN----AFD---
-----------------------------------------G-------KTI----------DKETGEV----
L-------------H--------------------QKTHFPQPWEF----------
FAQEVMIRVFGKPDGKPEFEEADTPEKLRTLLAEKLSSRPEAVHEYVTP------------------------
L----FVS-----RAPN-------RKMSGQGHMET--VKSA-KRL--D------E-------G---VS-----
-----------------V-----------------------------LRV-P-------LTQLKL--------
----------------------------KDLEKMVNR--ERE-------------------P--KLY-E----
----------------------------ALK-ARLE--AHKD---------------------D-----
PAKAFAEPFYKYDK-----------------------------------------------------------
-------------------------------------------------------------------
AGNRTQ--------------------QVKAVR----------------------
VEQVQKTGVWVRNHNGIADNATMVRVDVFE--------KG--DKYY---------------------------
---------------------------------------------------LVP--IY--SWQ----------
-----------VAKGILPDRAVVQGK----------------------DE-----------EDW--QL-----
-----------I-DDSFNFKFSLHPNDLVE--------------VIT---K-KARMFG--YF-A---------
----SC-H-RG-TG----------NI--------------------NIRIH--DL------------------
---------------DH-KIGKNGILEGIGVK-------------T--ALSFQK------------Y-Q----
IDELGKE--------------IRP--CRL--K-------K---RPPVR
>Enterococcus_faecalis tr|A0A0M2CKF7|A0A0M2CKF7_ENTFL CRISPR-associated
endonuclease Cas9 OS=Enterococcus faecalis EnGen0426 GN=cas9 PE=3 SV=1
-----M-----------------------------K---------------------------KD--
YVIGLDIGTNSVGWAVMTEDYQ-----L-VKKKMPIYGNTEKKKIKKNFWGVR--LFEEGHT-------
AEDRRLKRTARRRISRRRNRLRYLQAFFEEAMTDLDENFFARLQESFLVPEDK--KWHR--HPIF-AKL----
--------------ED---E----------V----------A-YHETY-------------------
PTIYHLRKKLADSSE--QADLRLIYLALAHIVKYRGHFLI-----------------------------E--
GK---LST------E----------------------------------------------------------
-------------------------------------------------------NISV--------------
-----KE----------QFQQFMIIYNQTFVNGESRLVSAPLPESVLIEEELTEKASRTKKSEKVLQQFPQE-
--------------------------------K------------------------A--------NGL----
----FG----------------QFLKLMVGNKADFKKVFGLEEEAKIT-------------------------
---------------------------------------
YASESYEEDLEGILAKVGDEYSDVFLAAKNVYDAVELSTILADSDKKSHAKLSSSMIVRFTEHQEDLKKFK--
-----------------
RFIRENCPDEYDNLFKNEQKDGYAGYIAHAGKVSQLKFYQYVKKIIQDIAGAEYFLEKIAQENFLRKQRTFDN
GVIPHQIHLAELQA----IIHRQAA-YYP-F-L----------KENQ---------
EKIEQLVTFRIPYYVGPL------SK--G----DASTF--AWL-------------------KR---------
---------------------------------------
QSEEPIRPWNLQETVDLDQSATAFIERMTNFDTYLPSEKVLPKHSLLYE--KFMVFNELTKISYTD--D----
--R----GIK----A-NFSGKEKEKIF-D-YL-FKT----------------R------RKVKK----KD---
--IIQ-F--------------Y---------RNEYNTEI-V--TLSGL--EE-----------DQFNASFST-
-------------------------------------------------------------------------
----------------------------------------------------YQDLLK------CG-LTR---
--------------------------------AE------------------LDHPDNAEKLEDIIKI----
LTIFE-DRQRI--RTQLS------TF--K-----G--QFSA-EV----LKKL-E-----------RKH-----
```

```
-YT----GWGRL---SKKL------------------------------------INGI-----
YDKESGKTILGYL----------------------------------------------------
IKDDGVSKH-YN----------R--N---FMQLIN-D-------SQ--LSFKNAI--QK-A--Q--SSEH--
EET---------L-SETV-NELAGSPAIKKGIYQSLKIVDELVAIMG-------------------
YAPKRIVVEMARENQTTS-TGKRR--SIQRLKIVE-KAMAEIGSNLL--KE----Q-------------P--
TT---NEQLR-D--TR-LFLYYMQNGK------DMYTG------D-----ELSLHRL-----
SHYDIDHIIPQSFM-KDD------SLDNLVLVGSTENRGKSDDVPSKEV-VKD--M----------
KAYWEKL--------Y-------A--------A-------------------------------------G-
LIS-QRKF-----------QRLTK-GEQGGLTLE-DKAHFIQRQLVE----TRQITKNVAGILDQRYNA---
----------NS------------K---------EKKVQIITL-KASLTSQFRSIF------------G---
----------------------------L-----------------------------------------Y-
KVREVN-DYHHGQDA-------YLNCVVATT-L--LK-V-YPNLAPEF-------V-YGEYP----------
-KFQTF-------------KENKATAKAIIYTNLLRFFTED-------EPR----------FTKDGEI----
L------------W--------------------S-N----S------------------------------
--------------------YLK---TI-KKE-----------------------L----
NYHQMNIVKKVE----------VQKGGFS--KESI-K-----------P-------K---------------
------------------------------------------------------------------------
----------------------GPSNKLIPVKNGLD-------------------P--QKY-GGFDS-----
---PIVAY-------------------------------------------------------------
TVLFTHE-----------------------------------------------------------------
--------------------------KGKKPLIKQEILGITIMEKTRF-EQNPILFLEEKGFLRPR------
--------------VLMKL-------------------------------------------PK-----YT-
---------
LYEFPEGRRRLLASAKEAQKGNQMVLPEHLLTLLYHAKQCLLPNQSESLAYVEQHQPEFQEILERVVDFAEVH
TLAKSKVQQIVKLF--EA--NQ--------------------------------------------------
------------------------------------------------------------------------
----TADVKEIAAS--FI--QL-M------------QF-N-AM-GA----------PS--------------
------TFK-F--FQ-------------------------------------------------K-
DIERARYTSIKE--IFDATIIYQSTTGLYETRR-K--------V----------------------------
------------VD
>Staphylococcus_aureus pdb|5AXW|A Chain A, Crispr-associated Endonuclease
Cas9
-----M----------------------------K---------------------------RN--
YILGLDIGITSVGYGIIDYETR-----D-----------------VIDAGVR--LFKEANVE-----
NNEGRRSKRGARRLKRRRRHRIQRVKKLLFDYN----LL-T----------------D--------------
--------------------H----------S----------E---LSG------------------
INPYEARVKGLS--Q--KLSEEEFSAALLHLAKRRGVHNV----------------------------N--
EV----EE------D-------------------T-------------------------------------
------------------------------------------------------GNELST------
KEQISRNSKALEE-----------------------------------------------------------
KYVAE--------------------------------LQ-LER-----------------LKK--------
DGE--------VRGS------------------------------------INR------------------
---------------------------------------------FKTSDY---------------------
------------------------------------------------------------------------
----------------------------------------------------------------------
VKEAKQ----LLKVQKA-YH--Q-L----D-----QSFI--------DTYIDLLETRRTYYEGPG------
EG---------SPF--GWK-----------------------------------------------------
---------------------------------DIKEWYEMLMGHCTYFPEELRSVKYAYNAD--
LYNALNDLNNLVITR--D------E------N----E-KLEYYEKFQII-E-NV-FKQ--------------
-K------KKPTL----KQ-----IAK-E-------------I-L-------V-----NE-E--DIKGY--
RVT-S---T---GKPEFTNLKV--------------------------------------------------
------------------------------------------------------------------------
--YHDIKD------IT-ARK----------------------------------EI----------------
--I---ENAELLDQIAKI----LTIYQ-SSEDI--QEELT------NL--N-----S--ELTQ-EE----
IEQI-S-N--------LKG------YT----GTHNL---SLKA-----------------------------
--------INLI--------------LDEL------------------------------------------
-----------------WH-TN---------D--N---QIAIFN-R--------LK--LV--PK----K---
---VDLSQ--QKE---------IPTTLV-DDFILSPVVKRSFIQSIKVINAIIKKYG---------------
```

-----LPNDIIIELAREKNSKD-AQKMI----NEMQKRN-RQTNERIEEII--RT----TG------------
---KE---NAKYL-I--EK-IKLHDMQEGK-------CLYSL------E-----AIPLEDL-
LNNPFNYEVDHIIPRSVS-FDN------SFNNKVLVKQEEASKKGNRTPFQYL-SSS--D---------S-
KISYETF--------K-------KH-ILNLAKG-----------K-------------------------G-
RIS-KTKK------------EYLLEER---DINRFSVQKDFINRNLVD----TRYATRGLMNLLRSYFRV---
----------N-----------------------NLDVKVKSI-NGGFTSFLRRKW-------------K---
----------------------------F--K-
KERNKG-YKHHAEDA-------LIIANADFI-F--K--E-WKKLD----------------------------
---KAKKVMENQMF----------
EEKQAESMPEIE-------------T--------------------EQ-----EYKE----------------
-----------I-FIT--P----H-------QIKHI-KD-FKD------------------------Y----
KYS-----HRVD-------KKPNRELINDT--LYST-RKD--D------K-------G--NTL----------
-----------IV-----------------------------NNLNG-------LYDKDN---DK---LKKL-
I---N------------------KSPEKLLMY--HHD-------------------P--QTY-Q---------
-----------------------KLK-LIME--QYGD---------------------E----------
KNPLYKYYE--
-------ET------GNYLT------K------------Y------------S-KK--------DNGP-----
---------------VIKKIKYY---G--N-KL---N--AHL-DI-TDDY--
PNSRNKVVKLSLKPYRFDVYL--DN--------GVYK------------------------------------
--FVT--VK--NLDV--I-K--KENYYE-----
---V------NS-KAYE----------------------EA-----------KKL--KK--------------
--I-SNQAEFIASFYNNDLIK--------------IN------GE--LY--RV-I-------------GV-
NNDL-LN----------RI--------------------EVN-M--ID-----------------------
ITYREYLENMND-K-RPPRIIKTI-AS-------------K--TQSIKK------------Y-S----
TDILGNL--------------YEV--K-S--K-------KHPQIIKKG
>Spiroplasma_helicoides gi|1060720631|ref|WP_069117188.1|:1-1069 type II
CRISPR RNA-guided endonuclease Cas9 [Spiroplasma helicoides]
-----M---KK--
VNIGLDIGIASVGWSIYDIENK-----K------------------IVKAGSR--
LFSEANAGTSKTSTTSDRREQRGRRRNLRRALRRRMDLIKLFVDFK----YI-N-----------------S-
---QQ------------------------D---F----------Y----------Q-L-DFN-----------
--------FNYLEKRNQALN--E--QISRDELLVLLFNFIKKRGSFNY-------------------------
----K--DDILEMKE------E--------------------K------------------------------
--KEEID------
-----ISKITDKKD---
-KLPVE---------------------------------IQ-LDN-----------------YKL--------
YGK--------YRGI-----NT-----------------------------EDSL------------------
--IAHEWY---------------------

KREIEK----ILETQVK-FN--V-V----D-----KDFC---------EKYLELFDRKRQYFDGPG------
WT--TSSKTQKSKY--GWK--
----------------------------------DEKEFFERLSGYDTYDSKEKRAPKHSMTSY--
LFNILNDLNNLKIEG--L-------------S----S-GLTYEQKYEII-N-SV-IEH---------------
-KEV--KNKNINL----KQ-----IAK-I--------------A-K-------V-----DV-S--GITGY--
RIK-K---N---NTPDFTNFEF---

--INKLRA------AS-IKA-----------------------------------NLD---YSFIK-------
--L---DNIKVLDQIAKI----LTVYQ-TAESR--KEQIL------KI--K----DI--EFDQ-NQ----
AEVI-S-----------LLS------FT----GTHSL---SIKT-----------------------------
--------MNKA--------------IEDM---
-----------------WY-EN----------K--N---HMQVFS-E--------KG--IK--PD----Y---
--NIKI-E--GKFTRL----P-VLRNKI-SEMYISPVVKRALIESIKIIKEIEKMND----------------
----LEIKDIVIELARESNSED-RKKYI----ADIQKKN-AKENSEIEEKY--KK----TV------------
---SK---VDIKT-K--TK-LILFNEQDGK-------CVYSG------K-----SIDVDRL-
LSEPNYCEIDHIIPFSVS-FDD------SRSNKVLVLREENQNKKQNTPWQYF-KEI--N---------R---
NWDEY--------K-------AR-VYNLYVT-----------NKK-------F---------------G-
KYG-TRKY------------ENLVFEK---NINDEEIQFSFINRNLND----TRYATSEVKNYLTFFKKE---

----------L-----------------------NKSYSIKTI-NGGFTNYIRNKFL------------H---
----------------------------L--
GKKDRD-D-YKHHAVDA-------TICAIAPII--
--DIKDGKT--
--LSMLEKDLNSDQVIK--------------------K-----------------------------------
--D-QLV--S----I--------IQDI--S-THQ------------------------Y----NFS-----
RKVE-------KRTNKQMFNET--IYST-RVT--K------E----------GLH------------------
---KI-----------------------------VRI-D-------ILSTEPSDIKN---LKEL-F---T---
---------------KDQHKLLIY--NSD-------------------K--KTY-E-----------------
---------------YLQ-KIFD--TYVN---------------------D--VDKENKPIKNPFYHFTY---
--EL---
---NEKIV------K------------Q------------S-NE--------ENPP-----------------
---IVRYLKYD---K--G-II---N--QFT-KI-THKYKNVKQNKEVVVVGSNALGYDLFY--SK----TL--
NMYK---
---------ILP--VT--HKVAYYD-TSNSDNKIK--------Y------RE-NDYE----------------
------IE-----------KAK--FK----------------I-DSTYIKKYTIFKYNELN------------
--FD----YKGE--NMTLLV-T-------------GF-N-SN-SE----------SL----------------
----EFC-Y--LE--KA--------------------------------KEPNKRIYKAI-K-----------
---Q--MKNIKL------------I-T----SNSTR--------------------------T-------K--
-IKIID
>Streptococcus_pyogenes_M1_GAS AAK33936.1 conserved hypothetical protein
[Streptococcus pyogenes M1 GAS]
-----M-----------------------------D---------------------------KK--
YSIGLDIGTNSVGWAVITDEYK-----V-PSKKFKVLGNTDRHSIKKNLIGAL--LFDSGET-------
AEATRLKRTARRRYTRRKNRICYLQEIFSNEMAKVDDSFFHRLEESFLVEEDK--KHER--HPIF-GNI----
--------------VD---E----------V----------A-YHEKY-------------------
PTIYHLRKKLVDSTD--KADLRLIYLALAHMIKFRGHFLI-----------------------------E--
GD---LNP------D--
---NSDV--------------
-----DK----------LF------IQLVQTYNQLFEENPINASGVDAKAILSARLSKSRRLENLIAQLPGE-
--------------------------------K------------------------K--------NGL----
----FG----------------NLIALSLGLTPNFKSNFDLAEDAKLQ-------------------------
----
LSKDTYDDDLDNLLAQIGDQYADLFLAAKNLSDAILLSDILRVNTEITKAPLSASMIKRYDEHHQDLTLLKAL
VR---------------------
QQLPEKYKEIFFDQSKNGYAGYIDGGASQEEFYKFIKPILEKMDGTEELLVKLNREDLLRKQRTFDNGSIPHQ
IHLGELHA----ILRRQED-FYP-F-L----------KDNR---------EKIEKILTFRIPYYVGPL-----
-AR--G-----NSRF--AWM-------------------TR--------------------------------
----------------KSEETITPWNFEEVVDKGASAQSFIERMTNFDKNLPNEKVLPKHSLLYE--
YFTVYNELTKVKYVT--E------GM---RKP----A-FLSGEQKKAIV-D-LL-FKT---------------
-N------RKVTV----KQ-----LKE-D--------------Y-F-------KKIECFDS-V--EISGV--
E------------DRFNASLGT---

--YHDLLK------II-KDK-----------------------------------DF----------------
--LDNEENEDILEDIVLT----LTLFE-DREMI--EERLK------TY--A-----H--LFDD-KV----
MKQL-K-----------RRR------YT----GWGRL---SRKL-----------------------------
--------INGI-----RDKQSGKTILDFL---
--------------KSDGF-AN----------R--N---FMQLIH-D--------DS--LTFKEDI--QK-A-
-Q--VSGQ--GDS---------L-HEHI-ANLAGSPAIKKGILQTVKVVDELVKVMGR---------------
----HKPENIVIEMARENQTTQ-KGQKN--SRERMKRIE-EGIKELGSQIL--KE----H-------------
P--VE---NTQLQ-N--EK-LYLYYLQNGR-------DMYVD------Q-----ELDINRL-----
SDYDVDHIVPQSFL-KDD------SIDNKVLTRSDKNRGKSDNVPSEEV-VKK--M-----------
KNYWRQL--------L-------N--------A-------------------------------------K-
LIT-QRKF------------DNLTK-AERGGLSEL-DKAGFIKRQLVE----TRQITKHVAQILDSRMNT---
----------KY----------DENDKL------IREVKVITL-KSKLVSDFRKDF-------------Q---
----------------------------F--Y-
KVREIN-NYHHAHDA-------YLNAVVGTA-L--IK-K-YPKLESEF--------V-YGDYK----
VYDVRKMIAKSEQEIGKATAKYFFYSNIMNFFKTEITLANGEIRKRPL-------IET----------

NGETGEI----V-------------W--------------------DKG----R-------------------
-------------------------------DFA---TV-RKV------------------------L----
SMPQVNIVKKTE-----------VQTGGFS--KESI-L-----------P-------K---------------
----
-----------------------RNSDKLIARKKDWD-------------------P--KKY-GGFDS-----
---PTVAY-----------SVLVVAK--
VEKGKSKKLKS--
-------------------------------VKELLGITIMERSSFE-KNPIDFLEAKGYKEVKKDLIIKL--
------------------PKYSL--FE----
-LE----------
NGRKRMLASAGELQKGNELALPSKYVNFLYLASHYEKLKGSPEDNEQKQLFVEQHKHYLDEIIEQISEFSKRV
ILADANLDKVLSAY--NK--HR---

----DKPIREQAEN--II--HL-F-------------TL-T-NL-GA----------PA--------------
------AFK-Y--FD--T-
TIDRKRYTSTKE--VLDATLIHQSITGLYETRI-D----LSQLG-----------------------------
------------GD
>Pasteurella_multocida WP_010907033.1 type II CRISPR RNA-guided endonuclease Cas9 [Pasteurella multocida]
-----M-----------------------------QT-----------------------TN-LS--
YILGLDLGIASVGWAVVEINEN-----E-D-------------PIGLIDVGVR--
IFERAEVPKTGESLALSRRLARSTRRLIRRRAHRLLLAKRFLKREG----IL-S-----------------
TI--DLE---KGLP---------
----------NQAWELRVAGLE--R--RLSAIEWGAVLLHLIKHRGYLSK-----------------------
------R--KN---ESQ------T--------------------N----------------------------
--NKELGA-L-
---LSGVAQNHQLLQS---
DDYRTPAE---------------------------------LALKKF-----------------AKE------
--EGH--------IRNQ-----R--------------------------G--AYTHT----------------
--FNRLDL-------------------

LAELNL----LFAQQHQ-FGNPH-C----K-----EHIQ---------QYMTELLMWQKPALSG---------
----
------------------------------------EAILKMLGKCTHEKNEFKAAKHTYSAE--
RFVWLTKLNNLRILE--D------GA-----E----R-ALNEEERQLLI-N-HP-YEK---------------
--------SKLTY----AQ-----VRK-L--------------L-G-------L-----SEQA--IFKHL--
RYS-K---EN-AESATFMELKA---

--WHAIRK------AL-ENQ-----------------------------------GLK---DT---W-----
QDLA---KKPDLLDEIGTA----FSLYK-TDEDI--QQYLT------N------------KVPN-SV----
INALLV-----------SLN------FD----KFIEL---SLKS-----------------------------
--------LRKI--------------LPLM---
-----------------EQ-GK----------R--Y---DQACR--E--------IYGHHY--GE--------
----ANQK--TSQ---------LLPAIP-AQEIRNPVVLRTLSQARKVINAIIRQYG----------------
-----SPARVHIETGRELGKSFKERREI----QKQQEDN-RTKRESAVQKF--KE----LF------S--D--
FS-SE---PKSKD-I--LK-FRLYEQQHGK-------CLYSG------K-----EINIHRL-N-
EKGYVEIDHALPFSRT-WDD------SFNNKVLVLASENQNKGNQTPYEWL-QGK--I---------N-
SERWKNF--------V-------AL-VLG---S-
QCS-AAKK------------QRLLT-Q---V---I-DDNKFIDRNLND----TRYIARFLSNYIQENLLL---
----------V----------------G------KNKKNVFTP-NGQITALLRSRW-------------G---
----------------------------L---IK-
AREN-N-NRHHALDA-------IVVACATPS-M--QQ-K-ITRFI-----------R-FKEVHPYKIENR---
---Y-------EMV----------DQESGEI----
I-------------S--------------------P--HFPEPWAY----------FRQ--------------
EVNIRVFDNHPDTVLKEMLPDRPQANHQFVQP------------------------L----FVS-----
RAPT-------RKMSGQGHMET--IKSA-KRL--A------E-------G---IS------------------
----V-----------------------------LRI-P-------LTQLKP---------------------

---------------NLLENMVNK--ERE-------------------P--ALY-A-----------------
---------------GLK-ARLA--EFNQ---------------------D-----PAKAFATPFYK------
----
--QGGQ-----------------
---QVKAIR----------------------VEQVQKSGVLVRENNGVADNASIVRTDVFI--------KN--
NKFF---
---------LVP--IY--TWQ---------------------VAKGILPNKAIVAHK----------------
------NE-----------DEW--EE----------------M-DEGAKFKFSLFPNDLVE------------
--LKT---K-KEYFFG--YY-I-------------GL-D-RA-TG----------NI----------------
----SLK-E--HD---------------------------------GEIS-KGKDGVYRVGVK----------
---L--ALSFEK------------Y-Q----VDELGKN--------------RQI--C--RPQ-------Q--
-RQPVR
>Mycoplasma_arthritidis_FUSION gi|754503441|ref|WP_041914105.1|:1-905
type II CRISPR RNA-guided endonuclease Cas9 [Mycoplasma arthritidis]
-----M-----------------------------E------------------------KK-TK--
VILGFDLGIGSVGWSIVNKETD-----D------------------IIDLGSR--LFPEPEL-------
AVKRREARSSRRINRRKKYRNLHFYREVVKHK----NIFG-----------------FS--SKDE--IT----
--------------SY---F----------L----------E-ANKKW-------------------
NNILELKCLALK--E--KVSPQELVYILHDYLKNRGFFYN-----------------------------L--
IS---EEQVEETLDEVNDPKKTKSKKSNKKSSLKAK-------------------------------------
---GDN--KEKFEPILD----
--SVDKR--LRPSE-
--------------------------------KL-FEA-----------------FKL--------YGF----
----SKEL-----SSI------------------------N--NDKYK-------------------------
---------------------------------------FHNNEW----------------------------
----
--VKEIED---
-LFATQG------F-T----D-----SEFA---------KHYLSRFKYVRSFAEGPG------SE--H----
SPSKY--GIY-------------------EKN------------------------------------E----
-------YGE--VVK----------KYNSIWEKTIGKCSVFEDEYRAPKQSPSAA--
LYNLLVDLANGWLDTFSVHNNVIIE------R----T-KIPSELKFKII-N-GI-LESI--K-K---------
-D-P--NKKSPEV----DR-----IFK-K--------------I-A-------T-----KE-L--KIKSP--
KFD-Y-----ESEKIRLVIMAF---

--Y----Q------SGIKDL----------------------------------SFLTLDLAIEGIE-----
KNSP---SWLNVYDNIAQI----LSKSI-DVDTR--ASELK------EY--FKKTEASEFLNSI-
KDDEFEFEKL-A-EILSLNLAKINNS------YS----LTSSL---SFKA-----------------------
--------------LRLF--------------IPKL-------------------------------------
-----------------------AK-TT----------S--N---EEELKFKD--------PD--IK--
SQIA--AKN----QEPKK--TKY---------IDAKQF-DDAVLPPSVKRTIKEAIGVLNQIIKLYSN-N---
--------------YEIAEIVVEMARDKNSKE-ETNRI----NKENKSN-KKSNQEIIKEI--KK----H---
--VSE--D--KY-NS---LPIST-K--EK-IKLLLQQKCK-------DVYDC------Q-----TITIEDV-
IRKPHLYEIDHIIPITVL-PDN------SFANKVITKQKNNQAKSNRTPYEWL-GSD--A------------
EKWSEL--------E-------SY-WSC-T-----TSGKIKENWNI-------NYFP------------
NKESL-AKKI------------KYLSL-N-----NKY-ELDDFLNRSLND----TRHSTKLFVDVLKAFFNN-
------------SSSYKEDAVDNVNS---K------NKKVKISTT-KGALTSLFRKCI-------------
TN-----K------------LLKNYNLPIENCW--
-FK-KDRD-K-FFHHAIDA-------SLIVFINKY-Y--LSQV-YG-I-------------------------
---NVFYK----------
DKRTGLI----L-------------ENAPINGKEHYEKFLFSNGFKKTN---DDWAR----------MKF---
-----------DETMN--K----IIEKIT----E---NV-VEK------------------------V----
KYS-----RKIL------AKRTNGELFNKL--RYGY-TEI--S------SDKKAAMKDHDSIN----------
-----------KI-----------------------------SKL-S-------LLSQDES---DLKKLETF-
F---S------------DNA-NENDKSKLLIY--KSK-------------------VGYSQY-K---------
-----------------------LLK-EIYN--KAE-----------FKQNELDGSKKN----------
INPFENYMK--
-------FL------ATKIDEIPEFQKIIPK-------ERFEAL-SRQKKFIL-MNSKFT----KVIA-----
---------------IIQNLKYE---F--K-TKTLDDVIFSKTIL--KK-

DGNGWKEAYFQESLTSFGCLVYK---H----KQKPKELK----------------------------------
--AVA--IN--AKNLKIV-N--NH--
FKTSLFEGLANNADTPDKQNEVLN----------------------KI-----------KSD--AG-------
---------I-GDEYEFYTALFKSQIFE-RIKTNESKDESVKNN----Y-DN--LA--YI-V-----------
--GI-S-IV-KR----------ST--------------------EIT-LYHLK--ENKFK-------------
----------KIGLQ-KRKRDSIRHNV-NE-------------F--FKKFRK------------V-D----
IDILGNI--------------K-----------------------RSK
>Mycoplasma_lipofaciens gi|652840048|ref|WP_027120764.1|:1-1146 type II
CRISPR RNA-guided endonuclease Cas9 [Mycoplasma lipofaciens]
-----M-----------------------------N-------------------------K-KN--
VTLGFDLGVGSVGWAILDNETN-----K------------------ILKLGSR--LFKEPKL-------
AIDRRKARSIRRSIRRKAYRNKKFYKLIIKYS----NIFS-----------------FE--NKEQ--IE----
--------------KS---F----------I----------L-LSKKY-------------------
NNILDLKLRGLN--S--KITKAELVWILHDYLENRGFFYT-----------------------------L--
AE---IQK------D--------------------K-------------------------------------
---KEQ--KIV----------
----IEN--MLPTE-
--------------------------------RM-YNF-----------------YKK--------FGF----
----AKNQHLYSEF--------------------------S--EYSKD-------------------------
---------------------------------------ISNKEW----------------------------
----
--VKELNI---
-LFKQQD--------I----D-----PNFT---------KAFLDLFTKIRSFEIGPG------SE--N----
SASPY--GVF-------------------VKT------------------------------------D----
-------DNK--IIK----------KYNTVWEKNIGHCSIYPNEYRALKNSIHAE--LFNVLNDLNNLRNYK-
-I------Q------T----F-RLTEENKKDIL-N-SL-IEKF--K-S----------N-QTKSTPKITI---
-KNFVLKPLLK-Y--------------I-NDNNIKIEL-----DK-S--DDYGF--KNP-E---G---
NDVKITELKN---
---LYTMIQ----
--IF-KEN-----------------------------------NSN---FNGVNV-----DNWE---
VFAHDFDEILNI----LSKSN-VVEQR--KSDLE------KL--S--NIKNYFD-KE-EQ----KDKT-I-
DLI---SQNNTLK------IS----NTSDL---SIKC------------------------------------
-YKEF--------------IPLL--
----------LQ-SN----------R--N---FEQIKFD---------RN--FG--NK----K-------
TKQN--GKY---------INDKFL-DKAILPPSVKTTMRESIKIFNKIIKLYSK-E-----------------
WNITKVVIEMAREKNGDE-VKKAI----SNLNKIN-KKRNEIIEAKI--KS----LK----GNS--------
NN---ISDAI-K--TK-AFLYFQQEHR-------DIYDG------K-----MLDFDKV-
INDPNYTQIDHVIPYSLS-LNN------SSANKVLTKTYHNQNKGQKTAAQYV-KEQ--N-----------
TWNWNEY--------L-------EF-CNKTYLN-----------GSK-------DMFP------------
SEKSQ-KQKY------------HNLLL-E---KFDKN-KQIEFMSRNLND----TRYATKMFKDELIDYAQS-
------------H-----------------------NKQFKVVCI-NGAITGYIRKIT-------------K-
----
NY-KNRD-D-YSHHAIDA-------SILSIIANN-T--KT-L-FNLLSLEDH-------R-YQPWI----
NND--N-----SISKI----------
DKVTGEI----I-------------Q--------------------INS---KFE------------KKK---
-----------MYDIE--N----IKNLVK-NSLN---NI-DSK------------------------V----
QFS-----RKQE-------PKNNAQLFNIN--LYGS-KVI--D------N-------N---IW----------
-----------KI-----------------------------QKI-K-------LLEATNK---D---LEKW-
F---N------------EKT---NKKEQLLIY--KHS-------------------K--KEY-E---------
-----------------------KIQ-NIFL--SFQN---------------------E----------
NKPFLSYMN--
-------SL------YITFP------NEFTK-------EAIQIL-VNEGKLLI-YDCI-S----RKKS-----
---------------IIGNLKYL---E--S-KI---P--NEN-SVVWHK----
NSNNKSFNDTLNSLGCLIYK---N----KK--NKYS-------------------------------------
---MLA--VN--SNIYFFG-Q--NN--TN------
--FLDETSYKQ-DSLL----------------------LF-----------KKE--KN---------------
-I-DINEKPLCFLNIGKSVL-------------NKT----T-QE--IL--YI-C-------------GI-V-
MN-NN----------TI--------------------ELK-Y--IN--KSMPI--------------------

----ICEKTL-KKQRIIKTC-NS-------------F--MNEYLP------------I-D----TDVLGNI--
-------------------------------------YK
>Staphylococcus_lugdunensis WP_002460848.1 type II CRISPR RNA-guided
endonuclease Cas9 [Staphylococcus lugdunensis]
-----M----------------------------N---------------------------QK--
FILGLDIGITSVGYGLIDYETK-----N-------------------IIDAGVR--LFPEANVE-----
NNEGRRSKRGSRRLKRRRIHRLERVKKLLEDYN----LL-D-----------------Q--------------
--------------------S----------Q----------I---PQS-------------------
TNPYAIRVKGLS--E--ALSKDELVIALLHIAKRRGIHKI-----------------------------D--
VI--DSND------D--------------------V------------------------------------
---GNELST------
KEQLNKNSKLLKD---
KFVCQ--------------------------------IQ-LER-----------------MN---------
EGQ--------VRGE-------------------------------------KNR-------------------
---FKTADI---------------------

IKEIIQ----LLNVQKN-FH--Q-L----D-----ENFI---------NKYIELVEMRREYFEGPG------
KG---------SPY--GWE-------------------G---------------------------------
----------------------------------DPKAWYETLMGHCTYFPDELRSVKYAYSAD--
LFNALNDLNNLVIQR--D------G------L----S-KLEYHEKYHII-E-NV-FKQ--------------
-K------KKPTL----KQ-----IAN-E--------------I-N-------V-----NP-E--DIKGY--
RIT-K---S---GKPQFTEFKL--

--YHDLKS------VL-FDQ----------------------------------SI----------------
--L---ENEDVLDQIAEI----LTIYQ-DKDSI--KSKLT------EL--D-----I--LLNE-ED----
KENI-A-Q---------LTG------YT----GTHRL---SLKC------------------------------
--------IRLV--------------LEEQ--
-----------------WY-SS----------R--N---QMEIFT-H--------LN--IK--PK----K---
---INLTA--ANK---------IPKAMI-DEFILSPVVKRTFGQAINLINKIIEKYG----------------
-----VPEDIIIELARENNSKD-KQKFI----NEMQKKN-ENTRKRINEII--GK----YG------------
---NQ---NAKRL-V--EK-IRLHDEQEGK-------CLYSL------E-----SIPLEDL-
LNNPNHYEVDHIIPRSVS-FDN------SYHNKVLVKQSENSKKSNLTPYQYF-NSG--K---------S-
KLSYNQF--------K-------QH-ILNLSKS-----------Q-------------------------D-
RIS-KKKK------------EYLLEER---DINKFEVQKEFINRNLVD----TRYATRELTNYLKAYFSA---
----------N----------------------NMNVKVKTI-NGSFTDYLRKVW-------------K---
----------------------------F--K-
KERNHG-YKHHAEDA-------LIIANADFL-F--K--E-NKKLK-----------------------------
---AVNSVLEK--P----------
EIESKQLDIQVD-------------S--------------------ED-----NYSE----------------
-----------M-FII--P----K-------QVQDI-KD-FRN------------------------F----
KYS-----HRVD-------KKPNRQLINDT--LYST-RKK--D------N----------STY----------
-----------IV-----------------------------QTIKD-------IYAKDN---TT---LKKQ-
F---D------------------KSPEKFLMY--QHD-------------------P--RTF-E---------
----------------------KLE-VIMK--QYAN---------------------E----------
KNPLAKYHE---
-------ET------GEYLT------K------------Y------------S-KK--------NNGP-----
---------------IVKSLKYI---G--N-KL---G--SHL-DV-THQF--
KSSTKKLVKLSIKPYRFDVYL--TD--------KGYK-----------------------------------
---FIT--IS--YLDV--L-K--KDNYYY-----
---I------PE-QKYD----------------------KL-----------KLG--KA--------------
--I-DKNAKFIASFYKNDLIK--------------LD------GE--IY--KI-I-------------GV-
NSDT-RN----------MI--------------------ELD-L--PD-----------------------
IRYKEYCELNNI-K-GEPRIKKTI-GK-------------K--VNSIEK------------L-T----
TDVLGNV--------------FTNTQY-T--K-------PQLLFKRGN
>Mycoplasma_canis gi|490932877|ref|WP_004794730.1|:1-1233 type II CRISPR
RNA-guided endonuclease Cas9 [Mycoplasma canis]

-----M-----------------------------E------------------------KK-RK--
VTLGFDLGIASVGWAIVDSETN-----Q------------------VYKLGSR--LFDAPDT-------
NLERRTQRGTRRLLRRRKYRNQKFYNLVKR-T----EVFG-----------------LS--SREA--IE----
--------------NR---F----------R----------E-LSIKY-------------------
PNIIELKTKALS--Q--EVCPDEIAWILHDYLKNRGYFYD-----------------------------E--
KE---TKE------D--------------------F-------------------------------------
---DQQ--T------------
-----VE--SMPSY-
--------------------------------KL-NEF-----------------YKK--------YGY----
----FKGA-----LSQ-----------PTESEMKDNKDLKE--AFFFD-------------------------
---------------------------------------FSNKEW----------------------------
----
--LKEINY---
-FFNVQK------NIL----S-----ETFI---------EEFKKIFSFTRDISKGPG------SD--N----
MPSPY--GIF-------------------GEF------------------------------------G----
----D--NG---QGG----------RYEHIWDKNIGKCSIFTNEQRAPKYLPSAL--IFNFLNELANIRLYS-
-T------DKKNIQPL----W-KLSSVDKLNIL-L-NL-FNLPISE-K----------K-K--KLTSTNI---
-NDIVKKESIK-S---IMISVEDIDMIKDEWAGKEPNV-----YG-V--GLSGL--NIE-E---
SAKENKFKFQDLKI---

LNVLIN------LL-DNV-----------------------------------GIK---FEFKDR-----
NDII---KNLELLDNLYLF----LIYQK-ESNNK--DSSID------LF--IAK--NESL--NI-
ENLKLKLKEF-L-LGA---GNEFENH------NS----KTHSL---SKKA-----------------------
--------------IDEI--------------LPKL-------------------------------------
-----------------------LD-NN----------EGWN---LEAIKNYD--------EE--IK--
SQIE--DNS--SLMAKQD--KKY---------LNDNFL-KDAILPPNVKVTFQQAILIFNKIIQKFSK-D---
--------------FEIDKVVIELAREMTQDQ-ENDAL----KGIAKAQ-KSKKSLVEERL--EA----NN--
--IDK--S--VFNDK----YEKL-I--YK-IFLWISQDFK-------DPYTG------A-----QISVNEI-
VN--NKVEIDHIIPYSLC-FDD------SSANKVLVHKQSNQEKSNSLPYEYI-KQG--H---------S-
GWNWDEF--------T-------KY-VKRVFVN-----------NVD-------SILS------------K-
KER-LKKS------------ENLLT-A---SYDGY-DKLGFLARNLND----TRYATILFRDQLNNYAEH---
----------HL----------ID---N------KKMFKVIAM-NGAVTSFIRKNM-------------SY--
---D----------------------NKL---RL-
KDRS-D-FSHHAYDA-------AIIALFSNK-T--KT-L-YNLIDPSL--------N-GIISK----RSE---
---G-----YWVIE----------DRYTGEI----
K-------------E--------------------LKK---EDWTS----------IKN--------------
NVQAR--K----IAKEIE-EYLI---DL-DDE------------------------V----FFS-----
RKTK-------RKTNRQLYNET--IYGI-ATKTDE------D-------GITNYY------------------
---KK-----------------------------EKF-S-------ILDDKD--------IYL----------
---------RLL---REREKFVIN--QSN-------------------P--EVI-D-----------------
---------------QII-EIIE--SYGKENNIPSRDEAINIKYTKNKINY----------NLYLKQYMR---
--SL---
---TKSL-------DQFSE-------EFINQM-IANKTFVL-YNPT-K----NTTR-----------------
---KIKFLRLV---N--DVKI---NDIRKNQVINKFN-GK-NNEPKAFYENINSLGAIVFK---N----SA--
NNFK---
---------TLS--IN--TQIAIFG-D--KN--WD--------IEDFKTYNM-EKIE----------------
------KY-----------KEI--YG----------------I-DKTYNFHSFIFPGTILL------------
-DKQ----N-KE--FY--YI-S-------------SI-Q-TV-RD----------II----------------
----EIK-F--LN--KIEFK------------------DENKNQ--DT-SKTPKRLMFGI-KS----------
---I--MNNYEQ------------V-D----ISPFGIN--------------K--------------------
--KIFE
>Hepatoplasma_crinochetorum AHK22391.1 CRISPR-associated Cas9 (formerly Csn1) protein [Candidatus Hepatoplasma crinochetorum Av]
-----M-----------------------------Q------------------------LE-NK--
KYLALDIGVTSLGWAISEYNNQKN-NWN------------------ILDFGVR--
LWDAPEDSKSLDTKTSERRQFRSGRRLNSRKKIRINDLKKTFYNHK----LL-K-----------------K-
-----ED--YQ-----------------NH---I--LKINKEGKK----------Y-FKDDK-----------
--------FNSIILRKKGLE--E--KLTNLELLIALINIAKRRGYSNR-------------------------

----FLIPNL--NEN-----KD--------------------A------------------------------
--RKSIEK--S--
-KEL---------I---
KKYKYPIKAITEDSFFNFNPKDLSKFNYRSRVFNESKFNEQIF-LDKNKKEINIINLQNLLKKYNID------
--HKNESEKELINILKNTLIKKY--------------------------N--DNQIL----------------
--FNREDY-------------------

ENEFDA----LLNKQIE-YND-K-L----------SDIK---------EDLKKIIFRQRDFEDGPG------
PK--D----PEVK---KIW-------------------KEK--------------------------------
----L--------NK-DS----KQ----------FYYKQFFENLGNCEFFQDQKRLSCFSIEND--
ISFILNETGKIFSKL--K------N------E----K--QINSEKIKII-TREI-FD---NYFK---------
-N-L--KFDRKII----TE-----IFS-K--------------H-N-------L-----NL-P--KLDGI--
S----------------FAN---

--SNLFLHSFAQNNEN-KEF--
ILKNLKIDLNFLN--T---IRDSKINKIANV----LFKNK-TPEKL--KNELI------KI-------DS--
FFNDQND----GYNW-IRK---------NNK------WIGKGNKTLST---SSKF------------------
-------------------ILSA--------------LKNQ--------------------------------
----------------------------LDTGE----------I--IFEYQNK-----------------IR-
-EKNT--E---------EK--LEKL-KEKEIKLFSPIKD-QDMQKNSVVFRAINQVRLVVRDLLKIH------
----------------NFDGLIIEVAKDLYAEKTLRNKI----RSNQDKN-QKIREESENKL--KE----HN-
--------------LI---PTSKN-I--NK-YLIWKDQQLDQISKKENFAYDLYDVDFKE-----
KIYLKNFIEDKNNEYQVDHIAPYSLV-NDD------TKNNKIVTSRKNNALKSNKTPLDFF-KIQ--N-----
----FTNKQLNNW--------K-------NK-I----EK----------------------------------
---S-INS-NVKL------------AYLFM-E---NLDRS-KETGFESRNIND----
TRYITKYITDYLKLEFAK-------------KE----------RKE--N------IKSPKILQI-
QGGITSYFRRLW-------------LN-----P-----S---------NYKYGSLWGD---------------
--------------------------IN-KPRDIS-PFHHAVDA-------IILSNMISE-Q--HI-
EFYQLIVRII--------NFYNYYV----NKK---
---D-----S--FNIKEKLFEQKRIIDNRFK----D-------------R--------------------G--
-----------------------FVYFYGTKELNNKLNQV--F----NYLINL-I---------
DQKIKTNQRLERIDFTTDQNILNLISPL----IEN-----LPSK-------I--------NN--LIPVRLKQ-
-I------N----------QKY---------------------PA----------------------------
-TTF-D-------SNTNEY--------IKE-------------------IKY---KKI-PAFL----------
---------------D--TIEAT--------------------------------EWA-NL-N--NKNI----
-----------------K----------DYPYVSYKI------------------------------------
-----------------------------------D-------------------------------------
--------KRIRGTLLAK-QNPS--------------------SKKEAIDQ---K--T-G-------------
----
--KLKNSFIKD--KK--
GN--------FWDISKY---------I-------------------GYTYDQNKKLIPIYRNQIAE-------
---------IAKQKEKINLILFKNAQFK--------------LS----N-DE--II--YTYK-----------
--SL-D-SL-EKRFSAPISNLTQH--------------------TNP-K--KY--KEMFN-------------
-------------------I-KLRISI-S--------------K--AQDLKL------------V-N----
ITRLGK--IE
>*Streptococcus_thermophilus_mycotype WP_011681470.1 type II CRISPR RNA-guided endonuclease Cas9 [Streptococcus thermophilus]
-----M-----------------------------T---------------------------KP--
YSIGLDIGTNSVGWAVTTDNYK-----V-PSKKMKVLGNTSKKYIKKNLLGVL--LFDSGIT-------
AEGRRLKRTARRRYTRRRNRILYLQEIFSTEMATLDDAFFQRLDDSFLVPDDK--RDSK--YPIF-GNL----
--------------VE---E----------K----------A-YHDEF-------------------
PTIYHLRKYLADSTK--KADLRLVYLALAHMIKYRGHFLI-----------------------------E--
GE---FNS------K--
---NNDI--------------
-----QK----------NF------QDFLDTYNAIFESDLSLENSKQLEEIVKDKISKLEKKDRILKLFPGE-
--------------------------------K------------------------N--------SGI----
----FS----------------EFLKLIVGNQADFRKCFNLDEKASLH-------------------------

----
FSKESYDEDLETLLGYIGDDYSDVFLKAKKLYDAILLSGFLTVTDNETEAPLSSAMIKRYNEHKEDLALLKEY
IR---------------------
NISLKTYNEVFKDDTKNGYAGYIDGKTNQEDFYVYLKKLLAEFEGADYFLEKIDREDFLRKQRTFDNGSIPYQ
IHLQEMRA----ILDKQAK-FYP-F-L----------AKNK---------ERIEKILTFRIPYYVGPL-----
-AR--G-----NSDF--AWS-------------------IR--------------------------------
----------------KRNEKITPWNFEDVIDKESSAEAFINRMTSFDLYLPEEKVLPKHSLLYE--
TFNVYNELTKVRFIA--E------SM---RDY----Q-FLDSKQKKDIV-R-LY-FKD---------------
-K------RKVTD----KD-----IIE-Y--------------L---------HAIYGYDG-I--ELKGI--
E------------KQFNSSLST---

--YHDLLN------II-NDK-----------------------------------EF----------------
--LDDSSNEAIIEEIIHT----LTIFE-DREMI--KQRLS------KF--E-----N--IFDK-SV----
LKKL-S-----------RRH------YT----GWGKL---SAKL-----------------------------
--------INGI-----RDEKSGNTILDYL---
--------------IDDGI-SN----------R--N---FMQLIH-D--------DA--LSFKKKI--QK-A-
-Q--IIGDEDKGN---------I-KEVV-KSLPGSPAIKKGILQSIKIVDELVKVMGG---------------
----RKPESIVVEMARENQYTN-QGKSN--SQQRLKRLE-KSLKELGSKIL--KE----N-----IPAKLS--
K--ID---NNALQ-N--DR-LYLYYLQNGK-------DMYTG------D-----DLDIDRL-----
SNYDIDHIIPQAFL-KDN------SIDNKVLVSSASNRGKSDDVPSLEV-VKK--R-----------
KTFWYQL--------L-------K--------S-------------------------------------K-
LIS-QRKF------------DNLTK-AERGGLSPE-DKAGFIQRQLVE----TRQITKHVARLLDEKFNN---
----------KK----------DENNRA------VRTVKIITL-KSTLVSQFRKDF-------------E---
----------------------------L--Y-
KVREIN-DFHHAHDA-------YLNAVVASA-L--LK-K-YPKLEPEF--------V-YGDYP----------
-KYNSFRERKSATEKVYFYSNIMNIFKKSISLADGRVIERPL-------IEV----------NEETGES----
V-------------W--------------------NKE----S------------------------------
--------------------DLA---TV-RRV------------------------L----
SYPQVNVVKKVEEQNHGLDRGKPKGLFNAN--LSSK-P-----------K-------P---------------
----
-----------------------NSNENLVGAKEYLD-------------------P--KKY-GGYAG-----
---ISNSF---TVLVKGTIEKGAKKK--
ITNVLEFQGIS--
-------------------------------ILDRINYRKDKLNFLL-EKGYKDIELIIELPKYSLFELSD--
------------------GSRRM--LA----
-SI----------
LSTNNKRGEIHKGNQIFLSQKFVKLLYHAKRISNTINENHRKYVENHKKEFEELFYYILEFNENYVGAKKNGK
LLNSAFQSWQNHSI--DE--LC---

----SSFIGPTGSE--RK--GL-F-------------EL-T-SR-GS----------AA--------------
------DFE-F--LG--
VKIPRYRDYTPSSL--LKDATLIHQSVTGLYETRI-D----LAKLG---------------------------
--------------EG
>Mycoplasma_spumans_PARTIAL WP_027123033.1 type II CRISPR RNA-guided endonuclease Cas9 [Mycoplasma spumans]
-----M-----------------------------EN--------------------N--KK-IE--
VSLGLDLGIGSVGWSLVNNETN-----E------------------VIALGSR--LFDEPNL-------
AEDRRAHRSLRRSIRRKALKKNTYAKLVIEYS----KMFN-----------------LDLKSVED--VL----
--------------NI---Y----------L----------K-SSQKH-------------------
PHIINLKYKALL--E--EISSEELIWILHDYLKNRGVFYE-----------------------------I--
ED---ETK------D--------------------K-------------------------------------
---KDG--DKNKSSFAE----
--FKDDS--KYPSE-
--------------------------------IE-KKY-----------------FDR--------FGF----
----VKNI-----E--------------------------A--NTGNL-------------------------
---------------------------------------FTNKRW----------------------------
----
--VAELEK---

-LFEVQSKKYDQ-K-L----F-----KEFS---------DKYLNLFKYIRSFEQGPG------NI--K----
SPSEY--GIF-------------------QRD-----------------------------------E----
-------NGE--VIQ---------KYTVIWEKTTGKCSVFKKDNRAPINTPSAE--MFNLLHNLNNTTFYI-
-D------E------NSQNKI-NLDNKVKKDLI-L-NW-FNSF--K-N---------D-K--KVKNIDK---
-K-----SIIK-E-------------M-K-------------KI-N--NDLTT--NSF-S---N---
EDIDYKNLNI--
--TNEILN----
--IF-VKN----------------------------------NCI---ASMFDG-----ENLF---
NVIKDLDKLYQP----IFYNR-SIDDR--INKLN------DKECI--KVFNKYLLND-SD----IQNT-I-
KDI---AQSSKLK------GN----KTHSL---SYRV-----------------------------------
-FEHT--------------LQAL---
----------FD-EI----------T--N---LESLKWNKESDLYKAVSE--YN--KE----N-
EIKLISKEQT--GKY---------LQANFL-DDLVVSPAVKSSIRESVKVFNQIIKEFGK-E----------
------YLITKVGLEMPRDKNGEE-EAKKI----SAQNKHN-KKVNDLIEAAV--KE----RA----MDN--
S-FSI-SN---CSDHT-K--LK-LLLWLQQDGI-------DLYSL------Q-----DIDLRKV-
YKDPGYTEIDHILPQSKS-FDD------SIRNKVLVLKESNRQKKNKVPKDFL-STE--K------------
-FEEL--------K-------KW-LYVHWKS-----------KAD-------
KYSSKKDKIKDLPWFKTYDEL-IAKY------------NNLLH-D---TLSQN-DEIEFASRNLND----
TRYACKEFLSILANYAKN-------------H-----------------------DNQFTIKPI-
RGKFTSIIRKLA-------------Q-------------------------------L--------------
--------------------------DK-KNRD-E-FDHHAIDA-------SILAIAANN-M--KM-N-
NNKWAILEN---------YYV------DKN--
-N-------NII----------DKETGEV----I-------------C-------------------KRS--
-DLT------------V------------------NTE--DISDKVKKSIGENRQQ---EI-KSK-------
----------------V----RFT-----RKLT-------KDHNIELFNAT--LYSS-IKD--P-----N--
-----DENIIY---------------------KV----------------------------ERK-S-----
--VYD-----------MKDY-F---E------------KE----DKHDNVLMF--RSH--------------
----P--QEF-N--------------------------------KLK-NIYL--EYKG--------------
------E----------KNPFEAYMN--
-----------------------DL------QKTYS-----ELISE-------EYKDFC-KNNQI-LL-L-
VE-N----NKVK--------------------MVKKLKII---D--K-KK---H---KN-TVCFD-----
KNSNKSFRESLSWIALLIYK---D----NK--NNYA------------------------------------
--YIP--VN--AKIYTFSND--KK--VD------
--LSNEDNY---

------------------------------------NL
>Spiroplasma_litorale gi|1125730088|ref|WP_075058359.1|:1-1069 type II CRISPR RNA-guided endonuclease Cas9 [Spiroplasma litorale]
-----M--KK--
VNIGLDIGIASVGWSIYDIDNK-----K------------------IINAGSR--
LFSESNSGSSNSSTTSDRRMQRGRRRNLRRLILRKQDLLKLFVKYN----YL-N----------------K-
---TN------------------------D---F----------Y---------N-L-NLN-----------
--------INYLEMRKKALK--E--KIAQEELIVLLFNYIKKRGSFNY------------------------
----K--DDLLESKK------D--------------------K-----------------------------
---FDDIS------
-----IDDIKKETN--
-LLPVE---------------------------------IQ-TNI----------------FNE--------
YGK--------YRGV-----KE----------------------------TDSL------------------
---IAHEWY---------------------

KKELEQ----ILNKQVE-EN--V-V----S-----QSFV---------QEYINLFDRKRLYFEGPG------
WT--TSSKTSKSEY--GWK---
----------------------------------DENEFYSRLTGFDTYNSNEKRAPKHSMTSY--
LFNILNDLNNINIDG--I-------------E----S-GLTYDQKYELI-E-SV-INH--------------
-NGP--KNKNITL----KL-----ISS-L-------------L-K-------V-----KE-S--DIKGY--

```
RID-K---E---NKPNFTKFEF---------------------------------------------------
-------------------------------------------------------------------------
--INKLRT------LL-INS-----------------------------------KLD---ISFIS-------
--L---KNIDLLDKISEI----LTIYQ-TAESR--KEKLL------ID--C----DY--SFSN-EQ----
AEVI-S-----------FIS------LT----GTHSL---SFKT-----------------------------
--------MKVA--------------IDEM-------------------------------------------
-----------------WY-DN----------K--N---HMQIFA-E--------KN--IK--PD----Y---
--NIKI-N--RKFNSM----P-LLRQKI-SEMYISPVVKRSLIESIKIIKEIEKIKD----------------
----LQIKDIVIELARESNSSD-FKKYI----NEIQKKN-EIENKEILEKH--KV----SL------------
---LK---KDFKT-R--LK-LILFNEQDGR-------CAYSG------T-----PIDIDRL-
YSDPNYCEIDHIIPFSVS-LDD------SRTNKVLVLWKENQDKGKKSPYQYF-RDK--N---------R---
NWNEF--------K-------EK-MYSLYVK-----------NKK-------L---------------G-
KYG-NKKY------------SNLVLEE---DINDVEVKQKFINRNLND----TRYATVEVKNYLTFFKKE---
----------L-----------------------NKSYSIKTI-NGGLTSYIRNVYL------------K---
----------------------------L------------------------------------------
PKKDRD-D-YKHHAIDA-------TICAIAPII----------------------------------------
----------------------------------------------------------------DLAEGKT--
--LNKILDSDDENIVLK--------------------L-----------------------------------
--E-ELG--D----L--------KNDI--N-NFA------------------------Y----KFT-----
KKVE-------KKSNTQLFNES--IYRC-IND--N------G----------NLI------------------
---KT-----------------------------EKI-D-------LLSLEPPKIKI---IKEL-F---T---
---------------TNKNKLLIY--ESD-------------------K--KTF-D-----------------
---------------YLE-NIYK--AYVN---------------------D--VDKNDKPVKNPFYHFVN---
--------------------------------------------------------------------EL---
---GERIT------K------------Q------------S-NE--------NTPP-----------------
---SIRYLRYK---G--S-VI---N--QYT-KI-THKFNKIKANKEIVMIGSNTIGWDLFY--SK----VY--
NLYK---------------------------------------------------------------------
---------VLP--IT--HNVAYFE-SNKSSSNIK--------Y------KL-KKYE----------------
------SE-----------KIL--YK----------------I-DETYEKKFTLHKNNELV------------
--FD----YDGQ--KFNLIV-V-------------GF-D-KT-HE----------RL----------------
----EFK-Y--LY--KK--------------------------------VDDNKRLHKTV-K-----------
---K--MKNIKL------------I-T----SNSTR--------------------------T-------K--
-VKIID
>**Streptococcus_thermophilus gi|500000239|ref|WP_011680957.1|:1-1121
type II CRISPR RNA-guided endonuclease Cas9 [Streptococcus thermophilus]
-----M---------------------------------------------------------SD--
LVLGLDIGIGSVGVGILNKVTG-----E------------------IIHKNSR--IFPAAQAE-----
NNLVRRTNRQGRRLARRKKHRRVRLNRLFEESG----LI-T-----------------D--------------
--------------------F----------T----------K-I-SIN-------------------
LNPYQLRVKGLT--D--ELSNEELFIALKNMVKHRGISYL-----------------------------D--
DA----SD------D--------------------G-------------------------------------
-------------------------------------------------------NSSVGD-Y----
AQIVKENSKQLET------------------------------------------------------------
KTPGQ---------------------------------IQ-LER-----------------YQT--------
YGQ--------LRGD-----FTV-----------E-------KDGKKH--RLINV------------------
----------------------------------------------FPTSAY---------------------
-------------------------------------------------------------------------
-----------------------------------------------------------------------
RSEALR----ILQTQQE-FNP-Q-I----T-----DEFI---------NRYLEILTGKRKYYHGPG------
NE--K----SRTDY--GRY-------------------RTS--------------------------------
-----------------GE---------------TLDNIFGILIGKCTFYPDEFRAAKASYTAQ--
EFNLLNDLNNLTVPT--E-------------T----K-KLSKEQKNQII-N-YV-KNE---------------
--------KAMGPAKLFKY-----IAK-L--------------L-S-------C-----DV-A--DIKGY--
RID-K---S---GKAEIHTFEA---------------------------------------------------
-------------------------------------------------------------------------
--YRKMKT------L--ET-------------------------------------LD---IE----------
--Q---MDRETLDKLAYV----LTLNT-EREGI--QEALE------HE--F---ADG--SFSQ-KQ----
VDEL-V-Q---------FRKANSSIFGK----GWHNF---SVKL-----------------------------
```

--------MMEL--------------IPEL---
-----------------YE-TS----------E--E---QMTILT-R--------LG--KQ--KT----T---
---SSSNK--TKY---------IDEKLL-TEEIYNPVVAKSVRQAIKIVNAAIKEYG----------------
-----DFDNIVIEMARETNEDD-EKKAI----QKIQKAN-KDEKDAAMLKA--AN---QYNGKAELPH--S--
V--FH---GHKQL-A--TK-IRLWHQQGER-------CLYTG------K-----TISIHDL-
INNSNQFEVDHILPLSIT-FDD------SLANKVLVYATANQEKGQRTPYQAL-DSM--D---------D-
AWSFREL--------K-------AF-VRE---S-------------------------------------K-
TLS-NKKK------------EYLLTEE---DISKFDVRKKFIERNLVD----TRYASRVVLNALQEHFRA---
----------H-----------------------KIDTKVSVV-RGQFTSQLRRHW-------------G---
----------------------------I--E-
KTRD-T-YHHHAVDA-------LIIAASSQL----N--L-WKKQK---N----TLVS-YS---------E---
---D-------QLL----------DIETGEL----
I-------------S--------------------DD-----EYKES-------VFKAP--------------
Y-QHF--V----D-------TLKSK-EF-EDS------------------------I----LFS-----
YQVD-------SKFNRKISDAT--IYAT-RQA--KVGKDKAD----------ETY------------------
---VL-----------------------------GKIKD-------IYTQDGY--DA---FMKI-Y---K---
---------------KDKSKFLMY--RHD-------------------P--QTF-E-----------------
---------------KVIEPILE--NYPN---------------------KQINEKGKEVPCNPFLKYKE---
--EH---
---G-YIR------K------------Y------------S-KK--------GNGP-----------------
---EIKSLKYY---D--S-KL---G--NHI-DI-TPK----DSNNKVVLQSVSPWRADVYF--NK----TT--
GKYE---
---------ILG--LK--YADLQFE-K--GTGTYK--------I------SQ-EKYN----------------
------DI-----------KKK--EG----------------V-DSDSEFKFTLYKNDLLL------------
--VKDTETKEQQ--LF--RF-L-------------SRTMPKQ-KH----------YV----------------
----ELK-P--YD--KQKFE----------------GGEALIKVLGNVA--NSGQCKKGL-GK----------
---S--NISIYK------------V-R----TDVLGNQ--------------HII--K-NEGD-------K--
-PKLDF
>Mycoplasma_gallisepticum gi|500213302|ref|WP_011883478.1|:1-1269 type II CRISPR RNA-guided endonuclease Cas9 [Mycoplasma gallisepticum]
-----M-----------------------------NN---------S---------IK--SK-PE--
VTIGLDLGVGSVGWAIVDNETN-----I------------------IHHLGSR--LFSQAKT-------
AEDRRSFRGVRRLIRRRKYKLKRFVNLIWKYN----SYFG-----------------FK--NKED--IL----
--------------NN---Y----------Q----------E-QQKLH-------------------
NTVLNLKSEALN--A--KIDPKALSWILHDYLKNRGHFYE-----------------------------D--
NR---DF--

------N--VYPTK-
--------------------------------EL-AKY-----------------FDK--------YGY----
----YKGI-----IDS-----------K---EDNDNKLEEE--LTKYK-------------------------
---------------------------------------FSNKHW----------------------------
----
--LEEVKK---
-VLSNQT------G-L----P-----EKFK---------EEYESLFSYVRNYSEGPG------SI--N----
SVSPY--GIY-------------------HLD------------------------------------E----
----K--EGK--VVQ----------KYNNIWDKTIGKCNIFPDEYRAPKNSPIAM--IFNEINELSTIRSYS-
-I------YL----TG----W-FINQEFKKAYL-N-KL-LDLLI-K-TNGEKPIDARQF-K--KLREETI---
-AESIGKETLK-D--------------VENEEKLEKED-----HK-W--KLKGL--KLN-T---N---
GKIQYNDLSS---
---LAKFVH----
--KL-KQH----------------------------------LKLD---FLLEDQ-----YATL---
DKINFLQSLFVY----LGKHL-RYNNRVDSANLK------EF--SDSNRLFER--VL-QEQKDGLFKL-F-
EQT---DKDDEKI------LA----QTHSL---STKA------------------------------------
-MLLA--------------ITRM--
----------TN-LDNDEDNQKNNDKGWN---FEAIKNFD--------QK--FI--DITK--TNN--
NLSLKQD--KRY---------LDDRFI-NDAILSPGVKRILREATKVFNAILKQFSQ-E--------------
---YDVTKVVIELARELSEEK-ELENN----KNYKKLI-KKNSDKISEGL--KA----LD----IAE--D--
KIEDIL--KSPTK-S--YK-VLLWLQQDHI-------DPYSQ------K-----EIAFEDI-

```
LTKTEKTEIDHIIPYSIS-FDD------SSSNKLLVLAESNQAKSNQTPYEFI-TSG--N---------A-
GIKWEDY--------E-------AY-CRK-FKD-----------GDT-------SLLD------------S-
TQR-SKKF------------AKMMK-T---DTSSK-YDIGFLARNLND----TRYATIVFRDALKDYANN---
----------HL----------VE---D------KPMFKVVCI-NGGVTSFLRKNF-------------DK--
---S----------------------WY-----------------------------------------AK-
KDRD-K-NIHHAVDA-------SIISIFSNK-T--KT-L-FDQLTQFA--------D-YKLFK----NTD---
-----------------------------------------G-----SWKKI----------DPKTGVV----
T------------E--------------------VTD---ENWKQ----------IRV--------------
RNQVS--K----IAEEID-KCIQDS-NI-ERK------------------------A----RYS-----
RKIE-------NKTNISLFNDT--VYSA-KKVGYD------D-------Q----I------------------
---KRKNLKTLDIDESVEENKNSKVKKQFVYRKLVNV-S-------LLNND---------KLA----------
---------DLF---AEKEDILMY--RAN-------------------P--WVI-N-----------------
---------------LAE-QIFN--EYTENR----------------KIKS----------QNVFGKYML---
---------------------------------------------------------------------DL---
---TKEFP------EKFSE-------AFVKSM-LRNKTAII-YNVE-K----KVVH-----------------
---RIKRLKIL---S--S-EL---KENKLSNVIIRSK-NE-SGTKLSYQDTINSVALMIMR---SIDPTAK--
KQYI------------------------------------------------------------------
---------RVP--LN--TLNLHLG-D--HD--FD--------LHNIDAYLKKPKFV----------------
------KY-----------LKA--NE-----------------I-GDEYKPWRVLISGSLLI------------
-HKR----D-KK--LM--YI-S-------------SF-Q-NL-ND----------LI----------------
----EIK-N--LI--ETEYK------------------E-NVDSDPKK-KKKASQILRSL-ST----------
---I--LNDYIL------------L-DAKDNFDILGLSKNRIDEILNSKLDLD--------------------
--KIAK
>Mycoplasma_cynos gi|505100601|ref|WP_015287703.1|:1-1239 type II CRISPR
RNA-guided endonuclease Cas9 [Mycoplasma cynos]
-----M-----------------------------E------------------------EK-RK--
VTLGFDLGVASVGWAIVDSETN-----E-----------------VYKLGSR--LFDIPDT-------
NIDRRAKRGSRRLVRRRSYRNQKFYNLIKR-T----KTFG-----------------FL--DKEA--IE----
--------------KE---F----------V----------L-LSIKY------------------
PNIIELKVKGLQ--E--EVSKSEAVWILHDYLKNRGYFYD-----------------------------D--
KE---EKD------D-------------------L---------------------------------------
-------------------------------------------------------ENP--S------------
-----IE-------------------------------------------------------------SMPSY-
--------------------------------KL-YEF-----------------YKE--------YGY----
----FKGA-----LSS-----------PTESEKNKDSDLEK--AIFFN-------------------------
----------------------------------------FSNKEW----------------------------
--------------------------------------------------------------------------
-----------------------------------------------------------------TKEINH---
-FFKIQK------NIF----S-----EDFK---------AKFMEFFSFIRDISKGPG------SE--N----
MPSQY--SIF-------------------GKY-------------------------------------G----
----D--DG---MGG----------KYQHIWDKNIGKCSIFKDKVRAPKYLPSAL--FFNLLNELSNIRLYS-
-V------DKREKSVL----W-RLSSKDKINII-L-NI-LQIILFK-N----------D-G--KISNLDI---
-NKIVKNESIK-S---IILNEIDLDLIKDMWFEKEPNV-----YG-I--GLSGL--NIE-E---
NKKENRFKFQDLKI------------------------------------------------------------
------------------------------------------------------------------
FSVFID------LL-KKS-----------------------------------NIN---IEFNNE-----
KDII---HNLKLLDELYFF----LITQK-YSRDK--YGSIK------KF--ILN--NKSF--NI-
DNLDEKLNIF-L-SIS---DDEFDNH------NS----KTHSL---SKEA-----------------------
--------------IYFI--------------FPKL--------------------------------------
------------------------LH-NN----------EGWN---LEAIKNYD--------ID--VR--
EEIS--KHS--FGIKKQD--KKY---------LDDAFL-DDAILPPSVKVTIKQSILIFNKIIKKFSN-K---
--------------FQIDNVVIELAREMTQEK-EMDAQ----KSLNRLK-KSRKKIIEERL--NA----NN--
--IDS--K--MFRDKTGEISPNY-I--YK-IYLWISQNFK-------DPYTG------E-----NISANDI-
LK--NNVEIDHIIPYSLC-FDD------SSSNKVLVFKHSNQSKGNFLPFDMI-STFSEN---------S-
VWNWKEY--------T-------QY-VEKTFKT-----------NLE-------SILD------------K-
KER-VKKA------------DNLLT-S---SYDGY-DKLGFLARNLND----TRYATILFRDQLINYSEN---
----------HL----------ID---S------KKMFKVIAM-NGGVTSFIRKNM-------------SF--
---D----------------------SGL-----------------------------------------KV-
```

KDRS-I-FSHHAYDA-------AIIALFSNK-T--KI-L-YNLINPSL--------D-GIISK----RSE---
---G-----YWVLE----------DRLTGEI----
R-------------K--------------------LNY---NDWQS----------IKN--------------
NVEVK--K----IAREIE-SHLS---NL-DNV------------------------V----MFS-----
RKSK-------RKTNKELYNET--LYGI-ATRIDE------N-------GIKNYY------------------
---KK-----------------------------EKF-N-------ILEDEN--------IYL----------
---------RLL---NERENFIIN--RSN-------------------P--EVI-D-----------------
---------------AII-EIFE--TIDKN-KIPARDVAKNIKYTDNKIKY----------NLYLKDYMR---
--KL---
---VDEFP------DKFRK-------DFIEQM-IAKKIFIL-FNPI-K----NTTR-----------------
---KIKSLRSI---N--NNKI---EDIRKKQVLEKFN-TA-KNEPRSFYESLNSLGAIIFK---N----NL--
NIYK---
---------KMS--IS--SQIATFG-D--KN--WK--------IDDINTYNK-EKLN----------------
------KY-----------KEI--YG----------------I-DETYDFYSFIFPGTILL------------
-DEK----N-KE--FL--YI-S-------------SI-Q-TL-KD----------KI----------------
----ELK-F--LD--RIEFE------------------KKNVNS--NL-KPKIKREIIST-KK----------
---L--MSDYKI------------I-K----ISPLGIN--------------K--------------------
--KIFE
>Francisella_tularensis WP_003038941.1 type II-B CRISPR-associated RNA-
guided endonuclease Cas9/Csx12 [Francisella tularensis]
-----M-----------------------------N-------------------------F-KI--
LPIAIDLGVKNTGVFSAFYQKGT----SLER---------------LDNKNGKVYELSKDSYT----
LLMNNRTARRHQRRGIDRKQLV-KRLFKLIWT---

----EQLN--L--EWDKDTQ-QAISFLFNRRGFSFITDGYSPEYLNIVPEQVKAILMDIFDDYNGE--DD---
LDS------Y--------------------
LKLATEQESKISEIYNKLMQKILEFKLMKLCTDIKDDKVSTKTLKEITSYEFELLADYLANYSESLKTQKFSY
TDKQGNLKELSYYHHDKYNIQEF--------------LKRHATIN----------------------------
--------------------------------DRILD---------------------------------TL-
LTD-----------------DLDIWNFNFEKFDF----------------------------
DKNEEKLQNQEDKDHIQAHLHHFVFAVNKIKSEMASGGRHRSQYFQEITNVLDENNHQEGYLKNFCENLHNKK
YSNLSVKNLVNLIGNLSNLEL--

--KPLRKYFNDKIHAKAD------H-W---DE---
--QKFTETYCHWILGEWRVGVKDQDK---KDGA------KY--S----YKDLC--NEL---------------
----KQK------------------------------------V----------------TKA----------
GLVDFLLELDPCRTIPPYLDNNNRKPPKCQSLILN-PKFLDNQYP----------------------------
----

NWQQYLQELKKLQSIQNYLDSFETDLKVLKSSKDQPYFVEYKSSNQQIASGQRDYKDLDARILQFIFDRVKAS
DELLLNEIYFQAKKLKQKASSELEKLESSKKLDEVIANSQLSQILKSQHTNGIFEQGTFLHLVCKYYKQRQRA
RDSRLYIMPEYRYDKKLHKYNNTGRFDDD--
--------------NQLLTYCNHKPRQKRYQLLNDLA------------------------------------
----
GVLQVSPNFLKDKIGSDDDLFISKWLVEHIRGFKKACEDSLKIQKDNRGLLNHKINIARNTKGKCEKEIFNLI
CKIEGSEDKKGNYKHGLAYELG--------------
VLLFGEPNEASKPEFDRKIKKFNSIYSFAQIQQIAFAERKGNANTCAVCSADNAHRMQQIKITEP--------
----
VEDNKDKIILSAKAQRLPAIPTRIVDGAVKKMAT-ILAKNIVDDNWQNIKQVLSAKHQLHIPIITESNAFE-
FEPAL----ADVKGKS-LKD--RRKKAL--ER--------------------------ISPEN-
IFKDKNNRIKEFAKGI-------SAYSG------A-----NLTDGDF-D--GAKEELDHIIPRSHK-KYG---
TLNDEANLICVTRGDNKNKGNRIFCLR---DL--A---------D-NYKLKQFETTDDLEIEKKIADTIWD-
A----N-----------------------------------KKDFKFGN-YRSFINLTPQEQKAFRHALFL-
AD-ENPIKQAVIRAINNRNRTFVNGTQRYFAEVLANNIYLRAKK-------------E---------------
NLNTDKISFDYFGIPTIGNGRGIAEIRQLY-------------EKVDSDIQ----------------------
AYA--K-GDKPQA-SYSHLIDA-------
MLAFCIAAD-E--HR-N-DGSIGL---
-------------------EIDKNYSLYPL----------DKNTGEV----F-------------T-------

-------------KD-----IFS--
----------------------QIKITDNEFSDKKLVRKKAI-----E---------GFNTHRQMTRDG--
IYAE-NYL-------------------PILIHKELNEVRKGYTWKNSEEIKIFK-------------------
----------GKK-YDIQQLNNLVY---------
CLKFVDKPISIDIQISTLEELRNILTTNNIAATAEYYYI--NLKTQKLHEYYIENYNTALGYKKYSKEM-E--
----
---------FLRSLAY----------RSERVKIKSIDDVKQVLDKDSNFIIGKITLPFKKEWQRLY-------
-REWQNTTIKDD------YEFLK------S---
---FFNVKSITKLHKKVRKDFS---

-------------------------LP--IS--TNEGKFLVK--------------RKTWDNNFI--------
-YQILNDSDSRADGTKPFIPAFDISKNEIVEAIIDSFTSKN--------------------------------
------------------------------------I-------------FW-L-PK-NI----------
ELQKVDNKNIFAIDTSKWFEVETPSDLRDIGIATIQYKIDNNSRPKVRVKLDYVIDDDSKINYFMNHS-
LLKSRYPDKVLE-------------I--LKQSTI------------I-E----FESSG---------------
------------------------FNKT
>Mycoplasma_ovipneumoniae gi|763430007|ref|WP_044285638.1|:1-1272 type II CRISPR RNA-guided endonuclease Cas9 [Mycoplasma ovipneumoniae]
-----M-----------------------------QKLIFWAERFKN---------MH--NK-KN--
ITIGFDLGIASVGWAIIDSQTS-----K------------------IIDWGTR--TFEERNT-------
ADKRRAFRSIRRNIRRKVYRNQKFINLILKYK----DLFE-----------------LE--NISD--
IQRVNKKDTENYEKIISFFTEI---Y----------R----------K-CAAKH-------------------
SNILEVKVKALD--S--KIEKLDLIWILHDYLENRGFFYD-----------------------------L--
EE---ENV------A--------------------D-------------------------------------
---K-----------------
---YEGI--EHPSI-
--------------------------------LL-YDF-----------------FKK--------NGF----
----FKSN-----SSI------------------------PKDLGGYS-------------------------
---------------------------------------FSNLQW----------------------------
----
--VNEIKK---
-LFEVQE--------I----N-----PEFS---------EKFLNLFSSVRDYAKGPG------SE--H----
SASEY--GIF-------------------QKD------------------------------------E----
-------DGK--VAK----------KYDNIWDKTIGKCSFFVEENRSPVNYPSYE--IFNLLNQLINLSTEL-
-K------T--TNKKI----W-QLSSNDRNELL-D-EL-LKVK----E----------N-A--KIISISL---
KKNEIKKIILK-D--------------F--EFEKSDID-----DQ-D--TIQGR--KII-K---E---EP--
TTKLEV---
---TKHLLA------
TI-YSH-----------------------------------SSN---SNWVNI-----NNIL---
EFLPYLDAICII----LDREK-S-RGQ--DEVLK------KL--TEKNIFEELKIDS-EK----QLDF-V-
KSI---FSNTKFN------FK----KIGNF---SLDA------------------------------------
-INLF--------------LPKM--
----------FE-QN----------K--N---SEYLKWKD--------EE--IR--KEWEIQKSK--
LGKTDKK--TKY---------LNPRIF-QDEIISPGTKNTFEQAVLVLNQIIKKYSK-E--------------
---NIIDAIVIESPREKNDKK-TIEEI----KERYKNS-KGKN--LEKLF--KI----LN----LEN--
SRYKL-SDLETKPGKL-L--DK-LRLYHQQDGI-------NLYTL------E-----KIVIDDL-
INNSQKYEIDHIIPYSMS-YDN------SQANKILTTKAENLKKGKLIASEYI-KTK--G-----------
DEFYNKY--------Y-------EK-AKELFNK----KNNTNKTNKKAKKIDYYVDL-------------
DEDSA-KNRF------------RFLTL-E---DYDE--FQVEFLARNLND----TRYSTKLFYHALIEHFEN-
------------NE------FFTYIDENSS------SHKVKISTI-KGHVTTYFRKKA-------------
NN-----I-----N----------------KQQ--
-IE-KNRE-N-NEHHAVDA-------AIVAIIGNE-N--RQ-I-ANLLTLADNKNDKKFIL-HD---------
E--N-------HKE----------
NIETGEL----V-------------K--------------------ISK----FE------------VDK---
-----------LAKVE--D----LEKIIQ-EKYEEA-KN-HIP------------------------I----
KFS-----RKIR-------NITNGGLSDET--LYGF-KYD--E------K-------E-DKYF----------
-----------KF-----------------------------IKK-N-------LVTTENK---D---LRKY-
F---KNPF--------GKKA-DGESEYTVLMA--QSH-------------------L--SEF-N---------

-----------------------KLK-EIFE--KYNG-----------------FSKDS----------
GKAFVEYMN--
-------DL------ALKEP----TLKAEIESAKAVDKLLYYNYKSSDEF-TY-YDN---INNKSFKR-----
---------------FYKNIKII---E---------YKSIPIKFKIVSK----
HDGGKSFKDKLFSLYSLVYKVYEK----GK--AIYK-------------------------------------
---SIP--VT--SQMRKFG-I--SE--FD------
--FLDENLYNK-EKLD----------------------IY-----------KSDFEKP---------------
-I-PVNCKPIFVLKKGSILKKKSLDIDDFKKTKDTE----N-GI--YY--FI-S-------------SM-T-
K--SS----------NV--------------------DT--I--YGLRPLNFK--------------------
-----------IERAVPHTT-NP-------------I--FKQYIP------------I-H----LDELGNE--
------------YPI--K-I--KEHNDDEKL---MCTIK
>Lactobacillus_rhamnosus WP_014569977.1 type II CRISPR RNA-guided endonuclease Cas9 [Lactobacillus rhamnosus]
-----M-----------------------------TK-----------------------LN-QP--
YGIGLDIGSNSIGFAVVDANSH-----L-LRL------------KGETAIGAR--LFREGQS-------
AADRRGSRTTRRRLSRTRWRLSFLRDFFAPHITKIDPDFFLRQKYSEISPKDKDRFKYE--KRLF--ND----
--------------RT---D----------A----------E-FYEDY-------------------
PSMYHLRLHLMTHTH--KADPREIFLAIHHILKSRGHFLT-----------------------------P--
GAAKDFNT------D--
---KVDL--------------
-----EDIFPALTEAYAQVYPDLELTFDLAKADDFKAKLLDEQATPSDTQKALVNLLLSSDGEKEIVKKRKQ-
--------------------------------V------------------------L--------TEF----
----AK----------------AITGLKTKFNLALGTEVDEADASNWQ-------------------------
----
FSMGQLDDKWSNIETSMTDQGTEIFEQIQELYRARLL-----
NGIVPAGMSLSQAKVADYGQHKEDLELFKTYLK-------------------
KLNDHELAKTIRGLYDRYINGDDAKPFLREDFVKALTKEVTAHPNEVSEQLLNRMGQANFMLKQRTKANGAIP
IQLQQRELDQ----IIANQSK-YYD-W-LAAPNPVEAHRWKMP---------
YQLDELLNFHIPYYVGPLITPKQQAE--S----GENVF--AWM-------------------VR---------
----
KDPSGNITPYNFDEKVDREASANTFIQRMKTTDTYLIGEDVLPKQSLLYQ--KYEVLNELNNVRINN--E---
-----------------CLGTDQKQRLI-R-EV-FER----------------H------SSVTI----KQ--
---VAD-N-------------L-V--------AHGDFARR-P--EIRGLADE------------
KRFLSSLST--
--YHQLKE-----
-IL-HE-------------------------------------A------------------
IDDPTKLLDIENIITW----STVFE-DHTIF--ETKLA------EI--E--------WLDP-KK----INEL-
S-----------GIR------YR----GWGQF---SRKL----------------------------------
---LDGL------KLGNGHTVIQEL--
------------ML-SN----------H--N---LMQILA-D--------ET--LK--ETMT-EL-N--Q--
DKLK--TDD---------I-EDVI-NDAYTSPSNKKALRQVLRVVEDIKHAANG-------------------
QDPSWLFIETADGTGTAG-KRTQS--RQKQIQTVYANAAQELIDSAV--RG----E-----LE---D--K--
IA---DKASF-T--DR-LVLYFMQGGR-------DIYTG------A-----PLNIDQL-----
SHYDIDHILPQSLI-KDD------SLDNRVLVNATINREKNNVFASTLF-AGK--M-----------
KATWRKW--------H-------E--------A-------------------------------------G-
LIS-GRKL------------RNLML-RP-DEIDK--FAKGFVARQLVE----TRQIIKLTEQIAAAQY-----
-----------------------------------PNTKIIAV-KAGLSHQLREEL-------------D---
----------------------------F--P-
KNRDVN-HYHHAFDA-------FLAARIGTY-L--LK-R-YPKLAPFF--------T-YGEFA----KVD---
------------------------VKKFREFNFIGALTHAKK-------NII----------AKDTGEI----
V-------------W--------------------DKE----R------------------------------
--------------------DIR---EL-DRI------------------------Y----
NFKRMLITHEVY--------FETADLFKQT--IYAA-KDS--K------E-------R---------------
----
-----------------------GGSKQLIPKKQGYP-------------------T--QVY-GGYTQ-----
---ESGSYNALVRVAEADTTAYQVIK--
ISAQNASKIAS--
-------------------------------ANLKSREKGKQLLNEI-VVKQLAKRRKNWKPSANSFKIVI--

------------------PRFGM--GT----
-LF----------
QNAKYGLFMVNSDTYYRNYQELWLSRENQKLLKKLFSIKYEKTQMNHDALQVYKAIIDQVEKFFKLYDINQFR
AKLSDAIERFEKLP--IN--TD---

----GNKIGKTETL--RQ--IL-I-------------GL-Q-AN-GT----------RS--------------
------NVK-N--LG--
IKTDLGLLQVGSGIKL--DKDTQIVYQSPSGLFKRRI-P----L---A-------------------------
----------------DL
>Spiroplasma_syrphidicola_FUSION gi|511280900|ref|WP_016341167.1|:1-773
type II CRISPR RNA-guided endonuclease Cas9 [Spiroplasma
syrphidicola]FUSION
-----M-----------------------------N-------------------------Y-KK--
LILGLDLGIASCGWAVTGQMEDG--NWV------------------LDDFGVR--
LFQTPENSKDGTTNAAARRLKRGARRLIKRRKNRIKDLKNLFEKIN----FI-N-----------------K-
----AS--LD------------------KY---INEHSATNLVED----------F-NRHEL-----------
--------YNPYFLRSIGIT--E--KLTREELVWSLIHIANRRGYKNK-------------------------
----FAFDI---EGD------G--------------------K------------------------------
--KRETK------

---------------------------------------LD-EAISNALISSNLTISQEIVRNKK--------
FRDAKNKKALLVRNKGGKEGE--------------------------N--NFQFL------------------
--FARDDY---------------------

KKEVDL----LLAKQAK-FYP-E-L----T-----EEIR---------AKAADIIFRQRDFEDGPG------
PK--K----QELR---EIY-------------------KKE--------------------------------
-----------------N-----K----------QFSKNFTQLEGRCTFLRELSVGYKSSILFD--
LFHIISEVSKISKYI--E------E------N----D-QLAQ----DII-S-SFLYN------E---------
-A-G--KKGKTLL----KE-----ILK-K--------------H-H-------I-----ND-DIFDTNAY---
-------K---NIDFKTNY--

-LNLLKE------VF-GND--VLKNLSL-----N-
-R---LEDNIYHQLGFI----IHTNI-TPERK--EKAIN------QW--LLE-NNI--ILAK-EK----
LNIL-LKP---------NSS------IS----TTVKT---SFKW-----------------------------
--------MSIA--------------ISNF---
-----------------LK-GI----------P--YGKFQAQFIK-E------------DN--FKLP--E---
--SYAKQY--QKYLTGEKTFEMFAPIID-PDLWRNPIVFRAINQARKVIKKLFEKYT----------------
-----FIDQINIELTREMGLSFSDRKKV----KERQDDS-LKENAKAKEFL--MA----NG------------
---II---VNDTN-V--LK-YKLWIQQNKK-------SLYSG------K-----EITIADL-G-
ASNVLQIDHIIPYSKL-ADD------SFNNKVLVFSKENQEKGNQFADQYV-KSL--G---------T--
ENYNNY--------K-------KR-VNYLLFQ-------------------------------------N-
QIN-QKKA------------EYLLC-S---NQNEE-ILNDFVSRNLND----TRYITRYVTNWLKAEFEL---
----------QS----------RF---G------LAKPKIMTL-NGAITSRFRRTW-------------LR--
---N-----S---------------PWGL---E--
KKSMLTTYRNKLSDSEWNELYSQVL--

KKWKNNNNLYI--P----NVENRL-F---------LIKNNKNSLSHYSLIDNLSNIITQRMPI----EL----
--TKE----------------
-----IV-----------------------------KQE-I-------VDRKTK--------EKY--------
-----------DVK---IAI-PKFVC--VKD-------------------P--DEY-----------------
-----------------LIK-NK-E--MIGN---------------------I----------RYPYASYKI-
--D--
---KKNSGDLLGSELPVP---------------
-----KTAKNLYK---D--G-E---

--------------------VNHLKYFVD--SK--NT--------IWNNESY---------------------

-AFLNIWKDDGKKHGYDYEFVKFNNYFK----------------DKEKFVNRGYKLYKSTLVR----------
----FI----R-DG--RY--HYKY-------------YQ-A-KM-GSKIYSNLLNTVFI--------------
------SVS-E--KS--KEIFT--------------------------------VQNSYDSL-SN--------
-----W--FNNLEI------------V-Q----IDILGNP--------------T------------------
----LVKI
>Mycoplasma_mobile gi|752715601|ref|WP_041362727.1|:1-1226 type II CRISPR RNA-guided endonuclease Cas9 [Mycoplasma mobile]
-----M-----------------------------N---------------------------KK--
VVLGLDLGIASVGWCLTDISQKEDNKFP------------------IILHGVR--LFETVDDSDDK-
LLNETRRKKRGQRRRNRRLFTRKRDFIKYLIDNN----II-E-----------------L-------------
-----------------E---F----------DKNPKILVRNFI---
EKYINPFSKNLELKYKSVTNLPIGFHNLRKAAIN--EKYKLDKSELIVLLYFYLSLRGAFFD-----------
------------------N--PE-----D------T--------------------K----------------
----
---SKEMNK-NE---IEIFDKNESIKNA---
---------------EFPID---------------------------------KI-IEF--------------
---YKI--------SGK--------IRST------------------------------------INLK----
--FGHQDY-------

------------LKEIKQ----VFEKQNI-DF--M-N----Y-----EKFA---------
MEEKSFFSRIRNYSEGPG------NE--K----SFSKY--GLY-------------------ANE--------
----------------------------NGNPELIINE--KGQKIYTK----------
IFKTLWESKIGKCSYDKKLYRAPKNSFSAK--VFDITNKLTDWKHKN---------------------E-
YISERLKRKILLS-RF-LNK----------------D------SKSAV----EK-----ILK-E---------
-----E-N-------I-----KF-E--NLSEI--AYN-K---D--DNKINLPIINA-----------------
----
------------------------------------YHSLTT------IF-
KKHLINFENYLISNENDLSKLMSFYKQQSEKLFVPNEKGSY---EINQN---------N---
NVLHIFDAISNI----LNKFS-TIQDR--IRILEGYFEFSNL--K-----K--DVKSSEI----YSEI-A-K-
--------LRE------FS----GTSSL---SFGA-------------------------------------
YYKF--------------IPNL---
---------IS-EGS---------K--N---YSTISYEE--------KA--LQ--NQ----K------
NNFSH--SNL---------FEKTWV-EDLIASPTVKRSLRQTMNLLKEIFKYSEKNN----------------
-LEIEKIVVEVTRSSNNKH-ERKKI----EGINKYR-KEKYEELKKVY--D-----L----------------
PN---ENTTL-L--KK-LWLLRQQQGY-------DAYSL------R-----KIEANDV-
INKPWNYDIDHIVPRSIS-FDD------SFSNLVIVNKLDNAKKSNDLSAKQF-IEK--IYGIEKLKE-A-
KENWGNW--------Y-------LRNA----NG-------------------------------------K-
AFNDKGKF------------IKLYTID---NLDEFD-NSDFINRNLSD----TSYITNALVNHLTF--SN---
----------S-----------------------KYKYSVVSV-NGKQTSNLRNQI-------------A---
----------------------------FVGIKNNKETEREWKRPEGFKSINSNDFLIREEGKNDVKDDVLI-
KDRS-F-NGHHAEDA-------YFITIISQY-F--R--S-FKRIE----------------------------
---RL-------NVNYRKETRELDDLEKNNI----
--KFKEKASFD--------------
N-FLL--I----N-------ALDELNEK-LNQ------------------------M----RFS-----
RMVI-------TKKNTQLFNET--LYSG-KYD--K------G-------K-NTIK------------------
---KV-----------------------------EKL-N-------LLDNRTDKIKK---IEEF-F---D---
---------EDKLKENELTKLHIF--NHD-------------------K--NLY-E-----------------
---------------TLK-IIWN--EVKI-------------EIKNKNLNE----------KNYFKYFVN---
----
KKLQEGKISFNEW------V------------P------------I-LD--------NDFK------------
--------IIRKIRYIKFSSEEK-ET---D--EIIFSQ-SNFLKIDQRQNFSFHNTLYWVQIWVYK---N---
-QK--DQYC--
--------------FIS--ID--ARNSKFE-K--DE--IK--------I------NY-EKLK-----------
-----------TQ-----------KEK--LQ----------------IIN--EEPILKINKGDLFE-------
-------NE----E-KE--LF--YI-V-------------GR-D-EK-PQ----------KL-----------
---------EIK-Y--IL-GK------------------------------KI-K-DQKQIQKPV-KK-----

--------Y--FPNWKK------------V-N----LTYMGEI------------------------------
--------FKK
>Legionella_pneumophila WP_011212792.1 type II-B CRISPR-associated RNA-guided endonuclease Cas9/Csx12 [Legionella pneumophila]
-----M-----------------------------E-------------------------S-
SQILSPIGIDLGGKFTGVCLSHLEAFA----ELPN---------------HANTKYSVILIDHNNFQ----
LSQAQRRATRHRVRNKKRNQFV-KRVALQLFQ---

----HILS--R--DLNAKEE-TALCHYLNNRGYTYV-----------------------------D--TD---
LDE------Y--------------------I--
--KDE--------------
TTINLLKE--
LLPSE---------------------------------SE-HNF-----------------
IDWFLQKMQSSEFR----------------------------
KILVSKVEEKKDDKELKNAVKNIKNFITGFEKNSVEGHRHRKVYFENIK------------
SDITKDNQLDSIKKKIPSVCLSNLLGHLSNLQW--

--KNLHRY----LAKNPK-----
-Q-F---DE-----QTFG---------NEFLRMLKNFR---HLKG------SQ--E----SLAVR--NLI---
----------------QQL------------------------------------E----------------
QSQ----------DYISILEKTPPEITIPPYEARTNTGMEKDQSLLLN-PEKLNNLYP---------------
----
-----------------------NWR---

NLIPGIIDAHPFLEKDLEHTKLRDRKRIISPSKQDEKRDSYILQRYLDLNKKIDKFKIKKQLSFLGQGKQLPA
NLIETQKEMETHFNSSLVSVLIQ--
--------IASAYNKEREDAAQGIWFDNAFS--
--
LCELSNINPPRKQKILPLLVGAILSEDFINNKDKWAKFKIFWNTHKIGRTSLKSKCKEIEEARKNSGNAFKID
YEEALNHPEHSNNKALIKIIQT--------------
IPDIIQAIQSHLGHNDSQALIYHNPFSLSQLYTILETKRDGFHKNCVAVTCENY----------W--------
----
RSQKTEIDPEISYASRLPADSVRPFDGVLARMMQ-RLAYEIAMAKWEQIKH-IPDNSSLLIPIYLEQNRFE-
FEESF----KKIKGSS-SDK--TLEQAI--EK-----------------------------
QNIQWEEKFQRIINASMNI-------CPYKG------A-----SIG---------GQGEIDHIYPRSLS-
KKHFGVIFNSEVNLIYCSSQGNREKKEEHYLLE---HL--S---------P-LYLKHQFGTDNVSDIK-----
--NF-I----S-----------------------------------QNVANIKK-
YISFHLLTPEQQKAARHALFL-DY-DDEAFKTITKFLMSQQKARVNGTQKFLGKQIMEFL-------------
------S---------------TLADSKQLQLEFSIKQI-TAEEVHDHRELL-------------S----
KQE----------------------PKL--V-
KSRQQS-FPSHAIDA-------TLTMSIGLK-E--F-----PQFSQ---------------------------
---ELD-----------------------------
-----------------------------------NS-----WFI----------------------------
--NHLMPDEVHLNPVRSKEKYN-----K---
------PNISSTPLFKDS--LYAE-RFI-------------------PVWV---------------------
KG-----------------------------ETF-AIGFSEKDLFE---------
IKPSNKEKLFTLLKTYSTKNPGESLQELQAKSKAKWLYF--PINKTLALEFLHHYFHKEIVTPDDTTVC-H--
----

FINSLRYYTKKESITVKILKEPMPVLSVKFESSKKNVLGSFKHTIALPATKDWERLFNHPNFLALKANPAPNP
KEF------NEFIR------K------------Y------------F-LS--------
DNNPNSDIPNNGHNIKPQKHKAVRKVFS---

-------------------------------LPVIPG--NAGTMMRIR--------------RKDNKGQPL--
-------Y-----
QLQTIDDTPSMGIQINEDRLVKQEVLMDAYKTRNLSTIDGINNSEGQAYATFDNWLTLPVSTFKPEIIK----
----------LE---MKPHSKTRR--YI-R-------------IT-Q-SL-AD----------
FIKTIDEALMIKPSDSIDDPLNMPNEIVCKNKLFGNELKPRDGKMKIVSTGKIVTYEFESDSTPQWIQ-

TLYVTQLKKQPI-------------K--EMLGMK------------L-A----GIYNE---------------
------------------------TSNN
>Campylobacter_jejuni YP_002344900.1 CRISPR-associated protein
[Campylobacter jejuni subsp. jejuni NCTC 11168 = ATCC 700819]
-----M--A--
RILAFDIGISSIGWAFSEN--D-----E------------------LKDCGVR--
IFTKVENPKTGESLALPRRLARSARKRLARRKARLNHLKHLIANEF----KL-N-----------------
YE--DYQS--FD------------------ES---L----------A----------KAYKGSL---------
----------ISPYELRFRALN--E--LLSKQDFARVILHIAKRRGYDDI-----------------------
------K---N---S-----------------------------D----------------------------
--DKEKGA-I-
---LKAIKQNEEKLA--
-NYQSVGE---------------------------------YLYKEY-----------------FQK-
FKENSKEFTN--------VRNK-----K--------------------------E--SYERC-----------
---IAQSFL--------------

-----KDELKL----IFKKQRE-FGF-S-F----S-----KKFE---------EEVLSVAFYKRAL-------
----
---KDFSHLVGNCSFFTDEKRAPKNSPLAF--
MFVALTRIINLLNNL--K------NT-----E----GILYTKDDLNALL-N-EV-LKN---------------
--------GTLTY----KQ-----TKK-L--------------L-G-------L-----SDDY--EFKG----
-------E---KGTYFIEFKK--

-YKEFIK------AL-GEH-----------------------------------NL-----------------
------SQDDLNEIAKD----ITLIK-DEIKL--KKALA------KY-----------DLNQ-NQ----
IDSL-S-----------KLE------FK----DHLNI---SFKA-----------------------------
--------LKLV--------------TPLM---
-----------------LE-GK----------K--Y---DEACN--E--------LN--LK--VA--------
----INED--KKD-FL----PAFNETYY-KDEVTNPVVLRAIKEYRKVLNALLKKYG----------------
-----KVHKINIELAREVGKNHSQRAKI----EKEQNEN-YKAKKDAELEC--EK----L-------------
-G-LK---INSKN-I--LK-LRLFKEQKEF-------CAYSG------E-----KIKISDL-Q-
DEKMLEIDHIYPYSRS-FDD------SYMNKVLVFTKQNQEKLNQTPFEAF-GND--S------------
AKWQKI--------E-------VL-A---K-
NLP-TKKQ------------KRILD-K---NYKDK-EQKNFKDRNLND----
TRYIARLVLNYTKDYLDFLPLSDDENTKLNDT------------Q---K------GSKVHVEAK-
SGMLTSALRHTW-------------G-------------------------------F---------------
--------------------------SA-KDRN-N-HLHHAIDA-------VIIAYANNS-I--VK-A-
FSDFK--
---------KEQ----------ESNSAEL--
--------------------------------YAKKISELDYKNKRKFFEPFSGFRQKVLDKIDE--------
----------------I----FVS-----KPER-------KKPSGALHEET--FR------------------
----

------------------------KE---

------EEFYQS--------------------YGGKEG----------------------
VLKALELGKIRKVNGKIVKNGDMFRVDIFK--HK----KT--NKFY---------------------------
---AVP--IYTMDFA----------
-----------LKVLPNKAVARSKKG----------------------EI-----------KDW--IL-----
-----------M-DENYEFCFSLYKDSLIL--------------IQT-KDM-QEPEFV--YY-
NAFTSSTVSLIVSKHD-N-KF-ET----------LS--------------------KNQKI--LF--------
-------------------------KN-ANEKEVIAKSIGIQ-------------N--LKVFEK---------
---Y-I----VSALGEV--------------TKA--EFR--Q-------R---EDFKK
>Eubacterium_rectale WP_012742555.1 type II CRISPR RNA-guided
endonuclease Cas9 [[Eubacterium] rectale]

```
-----M-----------------------------NY---------T---------EKEKLF-MK--
YILALDIGIASVGWAILDKESE-----T------------------VIEAGSN--IFPEASAA-----
DNQLRRDMRGAKRNNRRLKTRINDFIKLWENNN----LSIP-----------------Q--------------
--------------------F----------K----------------S-------------------
TEIVGLKVRAIT--E--EITLDELYLILYSYLKHRGISYL-----------------------------E--
DA----LD------D--------------------T-------------------------------------
-------------------------------------------------------VSGSSA-Y----
ANGLKLNAKELET------------------------------------------------------------
HYPCE---------------------------------IQ-QER-----------------LNT--------
IGK--------YRGQ-----SQI----------IN-------ENGEVL--DLSNV------------------
----------------------------------------------FTIGAY---------------------
-------------------------------------------------------------------------
-----------------------------------------------------------------------
RKEIQR----VFEIQKK-YHP-E-L----T-----DEFC---------DGYMLIFNRKRKYYEGPG------
NE--K----SRTDY--GRF-------------------TTK--------------------------------
---LD--------A--NGN--Y-I----------TEDNIFEKLIGKCSVYPDELRAAAASYTAQ--
EYNVLNDLNNLTING---------------------R-KLEENEKHEIV-E-RI-KSS---------------
--------NTINMR---KI-----ISD-C--------------M-G-------E-----NI-D--DFAGA--
RID-K---S---GKEIFHKFEV---------------------------------------------------
-------------------------------------------------------------------------
--YNKMRK------AL-LEI-----------------------------------GID---IS----------
--N---YSREELDEIGYI----MTINT-DKEAM--MEAFQ------KS--W---ID----LSD-DV----
KQCL-I-N---------MRKTNGAL-FN----KWQSF---SLKI-----------------------------
--------MNEL--------------IPEM-------------------------------------------
-----------------YA-QP----------K--E---QMTLLT-E--------MG--VT--KG----T---
--QEEFAG--LKY---------IPVDVV-SEDIFNPVVRRSVRISFKILNAVLKKYK----------------
-----ALDTIVIEMPRDRNSEE-QKKRI----NDSQKLN-EKEMEYIEKKL--AV---TYG--IKLSP--S--
D--FS---SQKQL-S--LK-LKLWNEQDGI-------CLYSG------K-----TIDPNDI-
INNPQLFEIDHIIPRSIS-FDD------ARSNKVLVYRSENQKKGNQTPYYYL-THS--H---------S-
EWSFEQY--------K-------AT-VMNLSKK-----------K---------E---------------Y-
AIS-RKKI------------QNLLYSE---DITKMDVLKGFINRNIND----TSYASRLVLNTIQNFFMA---
----------N-----------------------EADTKVKVI-KGSYTHQMRCNL-------------K---
----------------------------L------------------------------------------D-
KNRDES-YSHHAVDA-------MLIGYSELG-Y--E--A-YHKLQ----------------------------
-----------------------------------------G-------EFI----------DFETGEI----
L-------------R--------------------KD-----MWDENMSDEVYADYLYG--------------
K-KWA--N----I-------RNEVV-KA-EKN------------------------V----KYW-----
HYVM-------RKSNRGLCNQT--IRGT-REY--D------G----------KQY------------------
---KI-----------------------------NKL-D-------IRTKEGI--KV---FAKLAFSKKD---
---------------SDRERLLVY--LND-------------------R--RTF-D-----------------
---------------DLC-KIYE--DYSD---------------------A----------ANPFVQYEK---
--------------------------------------------------------------------ET---
---GDIIR------K------------Y------------S-KK--------HNGP-----------------
---RIDKLKYK---D--G-EV---G--ACI-DI-SHKYGFEKGSKKVILESLVPYRMDVYY--KE----EN--
HSYY---------------------------------------------------------------------
---------LVG--VK--QSDIKFE-K--GR--NV--------I------DE-EAYA----------------
------RILVNEKMIQPGQSRA--DL----------------E-NLGFKFKLSFYKNDIIE------------
--YE----KDGK--IYTERL-V-------------SRTMPKQ-RN----------YI----------------
----ETK-P--ID--KAKFE---------------------------------KQNLVGL-GK----------
------TKFIKK------------Y-R----YDILGNK--------------YSC--S-E--E-------K--
-FTSFC
>Mycoplasma_synoviae gi|752704781|ref|WP_041352097.1|:1-1269 type II
CRISPR RNA-guided endonuclease Cas9 [Mycoplasma synoviae]
-----M-----------------------------EN--------------------N--NK-EK--
IVIGFDLGVASVGWSIVNAETK-----E------------------VIDLGVR--LFSEPEK-------
ADYRRAKRTTRRLLRRKKFKREKFHKLILKNA----EIFG-----------------LQ--SRNE--IL----
--------------NV---Y----------K----------D-QSSKY-------------------
RNILKLKINALK--E--EIKPSELVWILRDYLQNRGYFYK-----------------------------N--
```

```
EK---LTD------E--------------------F-------------------------------------
-------------------------------------------------------V-----------------
-----SN------------------------------------------------------------SFPSK-
--------------------------------KL-HEH-----------------YEK--------YGF----
----FRGS-----VKLDNKLDNKKDKAKEKDEEEESDAKKE--SEELI-------------------------
---------------------------------------FSNKQW----------------------------
-------------------------------------------------------------------------
----------------------------------------------------------------INEIVK---
-VFENQS------Y-L----T-----ESFK---------EEYLKLFNYVRPFNKGPG------SK--N----
SRTAY--GVF-------------------STD------------------------------------I----
----DPETN---KFK----------DYSNIWDKTIGKCSLFEEEIRAPKNLPSAL--IFNLQNEICTIKNEF-
-T------EF----KN----W-WLNAEQKSEIL-K-FV-FTELF-NWK----------D-K--KYSDKKF---
-NK-NLQDKIK-KYLLNFALENFNLNEEILKNRDLEND-----TV-L--GLKGV--KYY-
EKSNATADAALEFSSLKP-------------------------------------------------------
-----------------------------------------------------------------------
LYVFIK------FL-KEK-----------------------------------KLD---LNYLLG-----
LENT---EILYFLDSIYLA----ISYSS-DLKER--NEWFK------KL-LKELYPKIKN--NN-
LEIIENVEDI-F-EIT---DQEKFES------FS----KTHSL---SREA-----------------------
--------------FNHI--------------IPLL-------------------------------------
-----------------------LS-NN----------EGKN---YESLKHSN--------EE--LK--
KRTE--K-A--ELKAQQN--QKY---------LKDNFL-KEALVPLSVKTSVLQAIKIFNQIIKNFGK-K---
--------------YEISQVVIEMARELTKPN-LEKLL----NN---AT-NSNIKILKEKL--DQTEKFDD--
--FTK--K--KFIDKI-ENSVVF-R--NK-LFLWFEQDRK-------DPYTQ------L-----DIKINEI-
ED---ETEIDHVIPYSKS-ADD------SWFNKLLVKKSTNQLKKNKTVWEYY-QNES-D---------P-
EAKWNKF--------V-------AW-AKRIYLVQKSDKESKDNSEKN-------SIFK------------N-
KKP-NLKF------------KNITK-K---LFDPY-KDLGFLARNLND----TRYATKVFRDQLNNYSKH---
----------HS----------KDD--E------NKLFKVVCM-NGSITSFLRKSM-------------WR--
---KNEEQVY--------------R-FNF-----------------------------------------WK-
KDRD-Q-FFHHAVDA-------SIIAIFSLL-T--KT-L-YNKLRVYE--------S-YDVQR----RED---
-----------------------------------------G-----VY-LI----------NKETGEV----
K-------------K--------------------ADK---DYWKD----------QHN--------------
FLKIR--E----NAIEIK-NVLNNV-DF-QNQ------------------------V----RYS-----
RKAN-------TKLNTQLFNET--LYGV-KEF--E------N----------NFY------------------
---KL-----------------------------EKV-N-------LFSRKD--------LRKF-I---
LEDLNEESEKNKKNEN---GSRKRILTE------------------------K--YIV-D-------------
-------------------EIL-QILENEEFKD------------------SKSD----------
INALNKYMD----------------------------------------------------------------
-------SL------PSKFS------EFFSQ-------DFINKC-KKENSLILTFDAI-KHNDPKKVI-----
---------------KIKNLKFF---R--E-D----ATLKNKQAV--HK-DS-
KNQIKSFYESYKCVGFIWLK---NKN--DL--EESI-------------------------------------
-----------------------------------------FVP--IN--SRVIHFG-D--KD--KD------
--IFDFDSYNK-EKLLNEI--------------NLKRPEN-----------KKF--NS---------------
-I-N-EIEFVKFVKPGALLL-------------NFE----N-QQ--IY--YI-S-------------TL-E-
SS-SL----------RA--------------------KIK-L--LN--KMD----------------------
-------------KGKAVSM-KK-------------ITNPDEYKI------------IEH----VNPLGIN--
------LNWTKKL----------------------ENNN
```

Figure S1. Multiple alignment of Cas9 proteins.
