# Supplementary material for: Multiple Origins and Specific Evolution of CRISPR/Cas9 Systems in Minimal Bacteria (Mollicutes)
Source: Front Microbiol. 2019 Nov 21;10:2701. doi: 10.3389/fmicb.2019.02701 (PMC6882279; doi:10.3389/fmicb.2019.02701)
Supplement: Supplementary file 3 [file Presentation_2.pptx]

## Slide 1
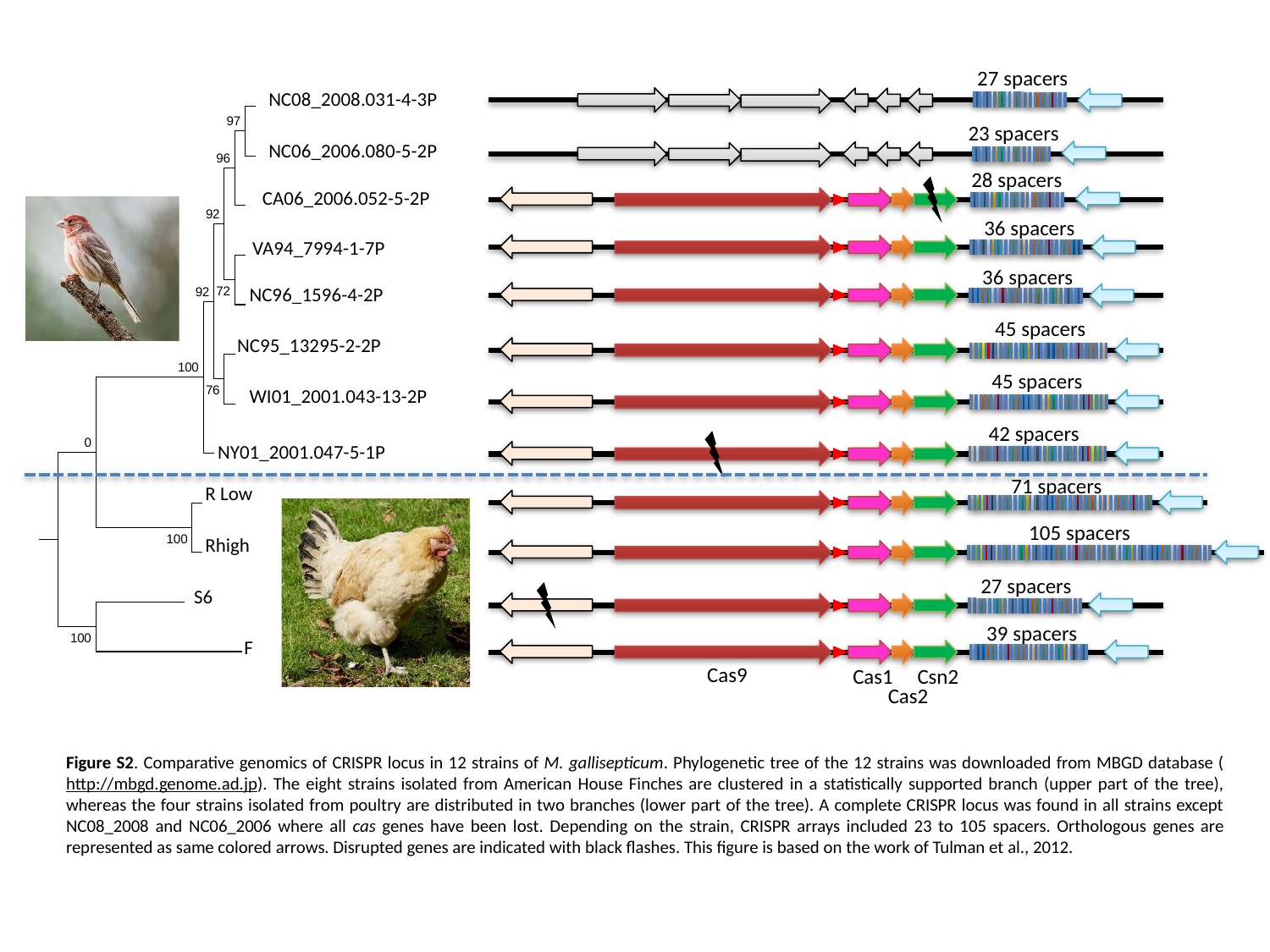

27 spacers
NC08_2008.031-4-3P
23 spacers
NC06_2006.080-5-2P
28 spacers
CA06_2006.052-5-2P
36 spacers
VA94_7994-1-7P
36 spacers
NC96_1596-4-2P
45 spacers
NC95_13295-2-2P
45 spacers
WI01_2001.043-13-2P
42 spacers
NY01_2001.047-5-1P
71 spacers
R Low
105 spacers
Rhigh
27 spacers
S6
39 spacers
 F
Cas9
Cas1 Csn2
Cas2
Figure S2. Comparative genomics of CRISPR locus in 12 strains of M. gallisepticum. Phylogenetic tree of the 12 strains was downloaded from MBGD database (http://mbgd.genome.ad.jp). The eight strains isolated from American House Finches are clustered in a statistically supported branch (upper part of the tree), whereas the four strains isolated from poultry are distributed in two branches (lower part of the tree). A complete CRISPR locus was found in all strains except NC08_2008 and NC06_2006 where all cas genes have been lost. Depending on the strain, CRISPR arrays included 23 to 105 spacers. Orthologous genes are represented as same colored arrows. Disrupted genes are indicated with black flashes. This figure is based on the work of Tulman et al., 2012.
