# Supplementary material for: Multiple Origins and Specific Evolution of CRISPR/Cas9 Systems in Minimal Bacteria (Mollicutes)
Source: Front Microbiol. 2019 Nov 21;10:2701. doi: 10.3389/fmicb.2019.02701 (PMC6882279; doi:10.3389/fmicb.2019.02701)
Supplement: Supplementary file 4 [file Presentation_3.pptx]

## Slide 1
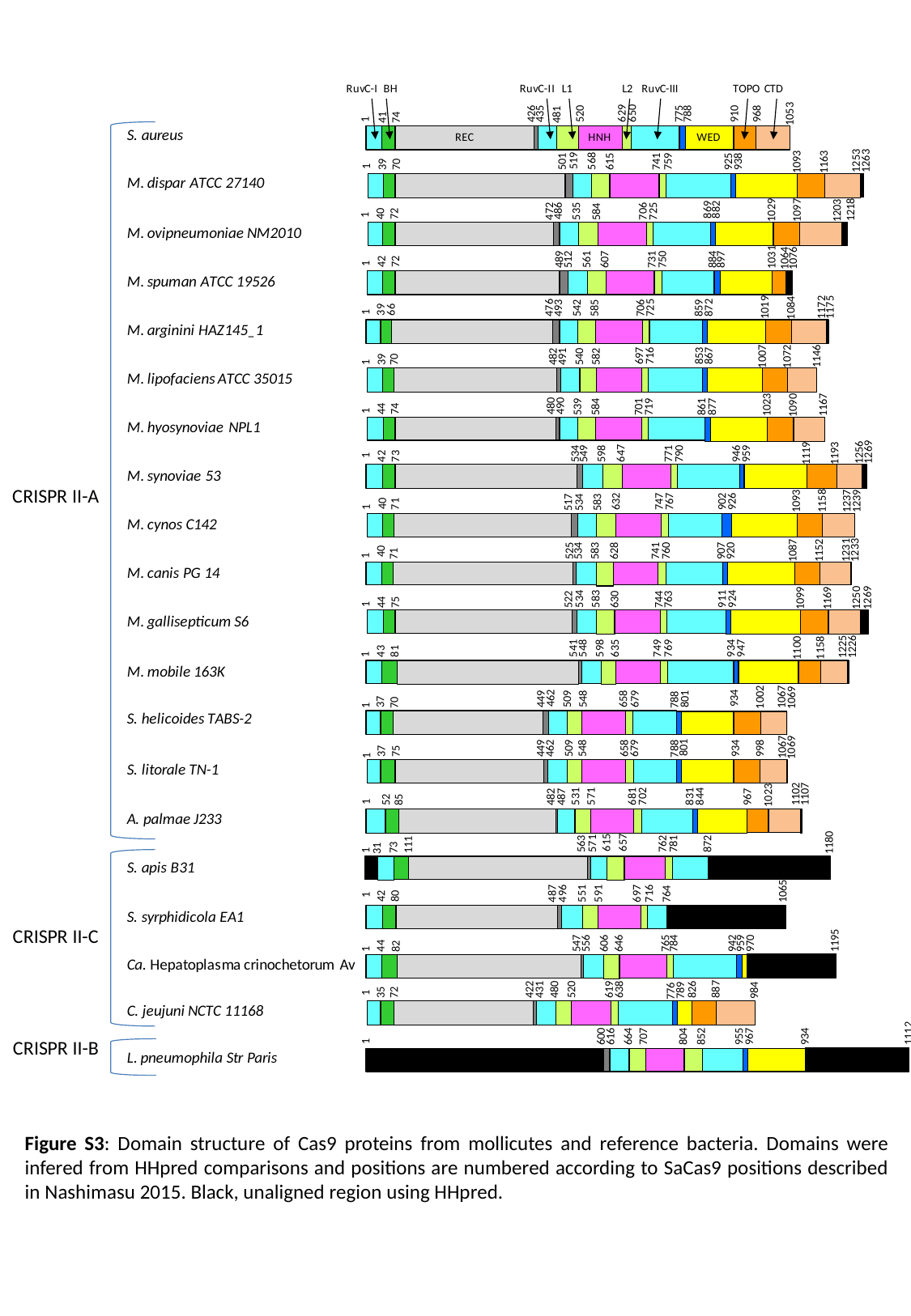

Figure S3: Domain structure of Cas9 proteins from mollicutes and reference bacteria. Domains were infered from HHpred comparisons and positions are numbered according to SaCas9 positions described in Nashimasu 2015. Black, unaligned region using HHpred.
