# Supplementary material for: Multiple Origins and Specific Evolution of CRISPR/Cas9 Systems in Minimal Bacteria (Mollicutes)
Source: Front Microbiol. 2019 Nov 21;10:2701. doi: 10.3389/fmicb.2019.02701 (PMC6882279; doi:10.3389/fmicb.2019.02701)
Supplement: Supplementary file 5 [file Presentation_4.pptx]

## Slide 1
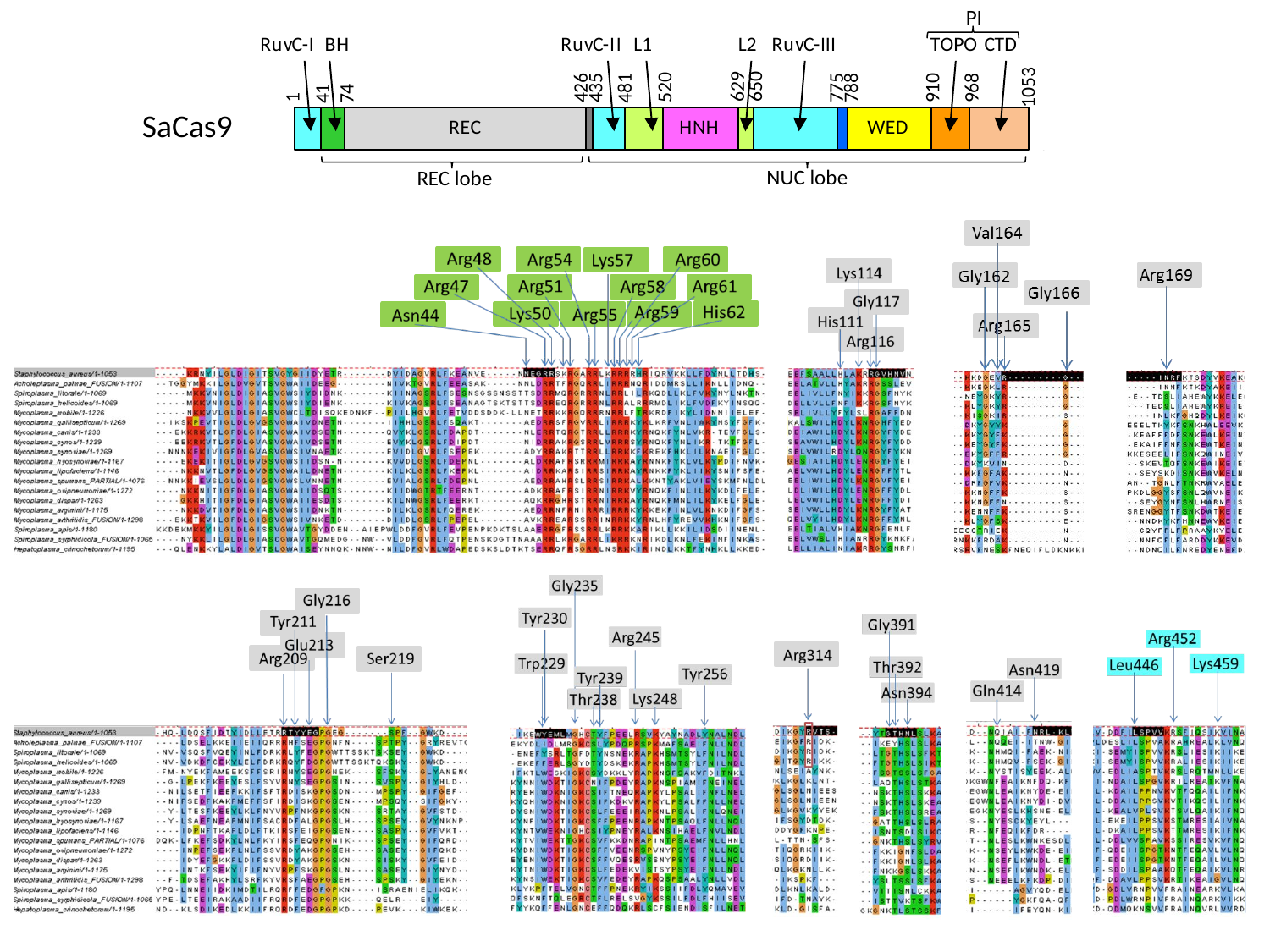

PI
SaCas9
NUC lobe
REC lobe

## Slide 2
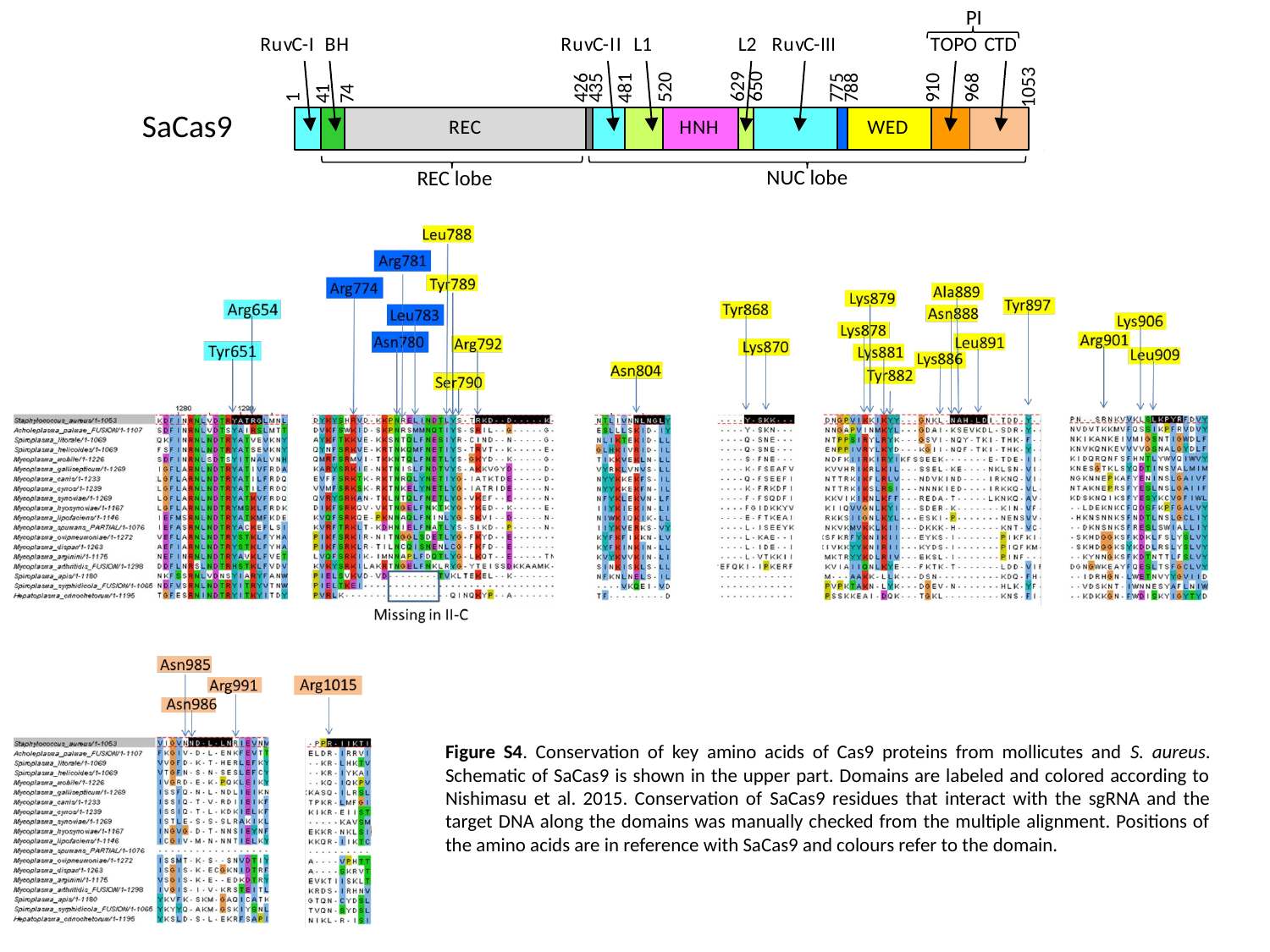

PI
SaCas9
NUC lobe
REC lobe
Figure S4. Conservation of key amino acids of Cas9 proteins from mollicutes and S. aureus. Schematic of SaCas9 is shown in the upper part. Domains are labeled and colored according to Nishimasu et al. 2015. Conservation of SaCas9 residues that interact with the sgRNA and the target DNA along the domains was manually checked from the multiple alignment. Positions of the amino acids are in reference with SaCas9 and colours refer to the domain.
