# Supplementary material for: Multiple Origins and Specific Evolution of CRISPR/Cas9 Systems in Minimal Bacteria (Mollicutes)
Source: Front Microbiol. 2019 Nov 21;10:2701. doi: 10.3389/fmicb.2019.02701 (PMC6882279; doi:10.3389/fmicb.2019.02701)
Supplement: Supplementary file 6 [file Presentation_5.pptx]

## Slide 1
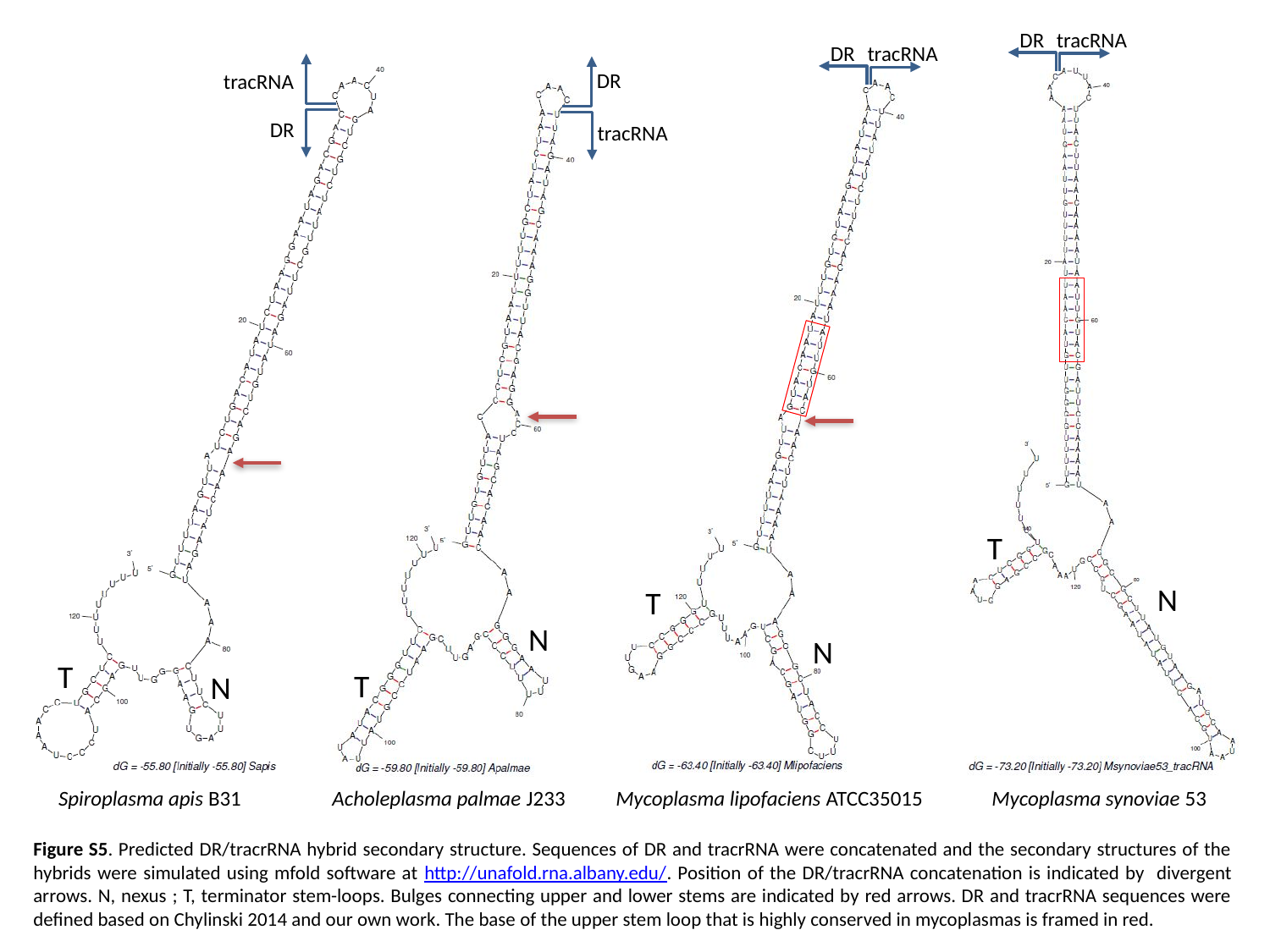

DR
tracRNA
DR
tracRNA
DR
tracRNA
DR
tracRNA
T
N
T
N
N
T
T
N
Spiroplasma apis B31
Acholeplasma palmae J233
Mycoplasma lipofaciens ATCC35015
Mycoplasma synoviae 53
Figure S5. Predicted DR/tracrRNA hybrid secondary structure. Sequences of DR and tracrRNA were concatenated and the secondary structures of the hybrids were simulated using mfold software at http://unafold.rna.albany.edu/. Position of the DR/tracrRNA concatenation is indicated by divergent arrows. N, nexus ; T, terminator stem-loops. Bulges connecting upper and lower stems are indicated by red arrows. DR and tracrRNA sequences were defined based on Chylinski 2014 and our own work. The base of the upper stem loop that is highly conserved in mycoplasmas is framed in red.
