# Supplementary material for: Multiple Origins and Specific Evolution of CRISPR/Cas9 Systems in Minimal Bacteria (Mollicutes)
Source: Front Microbiol. 2019 Nov 21;10:2701. doi: 10.3389/fmicb.2019.02701 (PMC6882279; doi:10.3389/fmicb.2019.02701)
Supplement: Supplementary file 7 [file Presentation_6.pptx]

## Slide 1
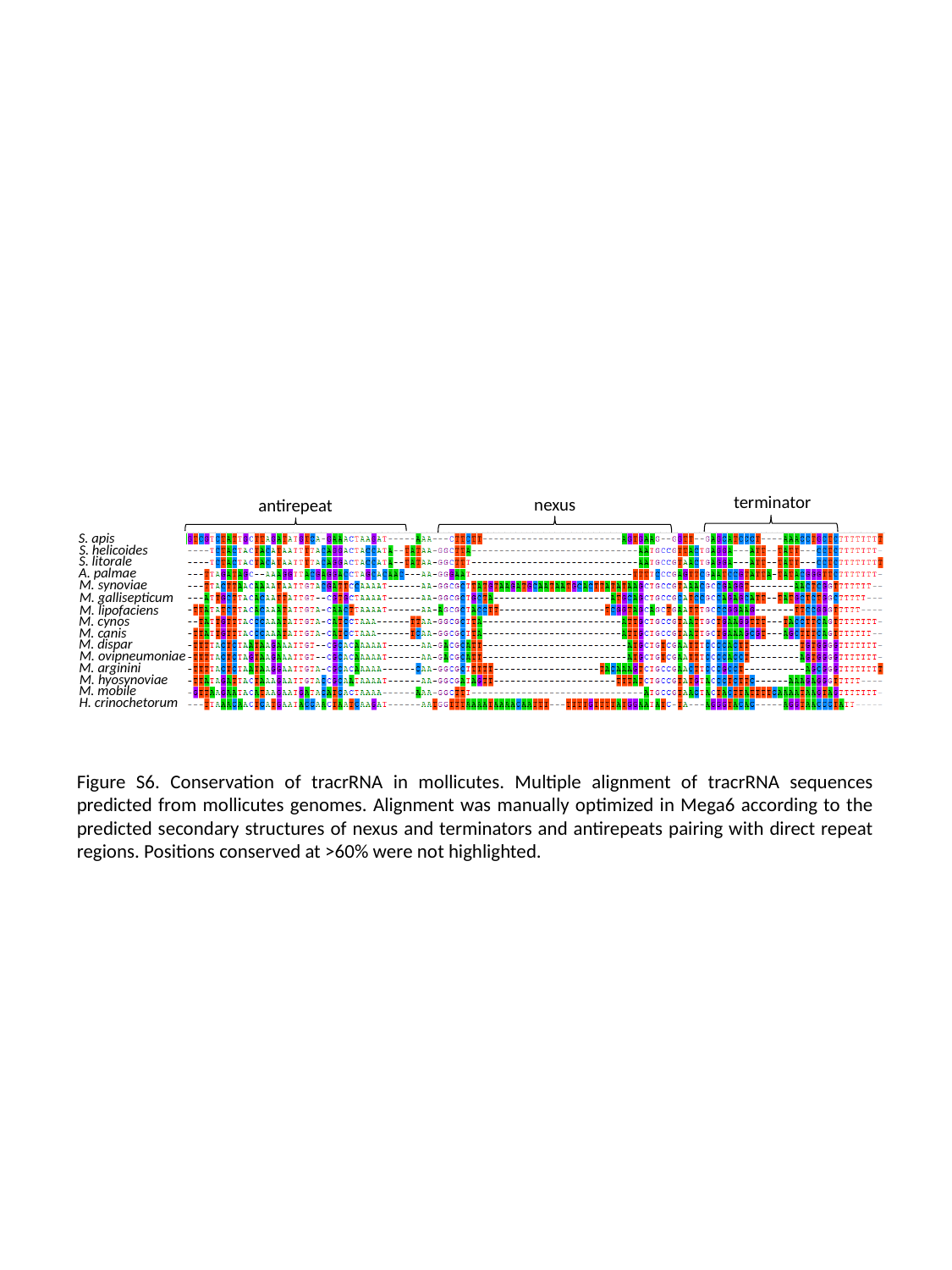

terminator
nexus
antirepeat
S. apis
S. helicoides
S. litorale
A. palmae
M. synoviae
M. gallisepticum
M. lipofaciens
M. cynos
M. canis
M. dispar
M. ovipneumoniae
M. arginini
M. hyosynoviae
M. mobile
H. crinochetorum
Figure S6. Conservation of tracrRNA in mollicutes. Multiple alignment of tracrRNA sequences predicted from mollicutes genomes. Alignment was manually optimized in Mega6 according to the predicted secondary structures of nexus and terminators and antirepeats pairing with direct repeat regions. Positions conserved at >60% were not highlighted.
