# Supplementary material for: Multiple Origins and Specific Evolution of CRISPR/Cas9 Systems in Minimal Bacteria (Mollicutes)
Source: Front Microbiol. 2019 Nov 21;10:2701. doi: 10.3389/fmicb.2019.02701 (PMC6882279; doi:10.3389/fmicb.2019.02701)
Supplement: Supplementary file 8 [file Presentation_7.pptx]

## Slide 1
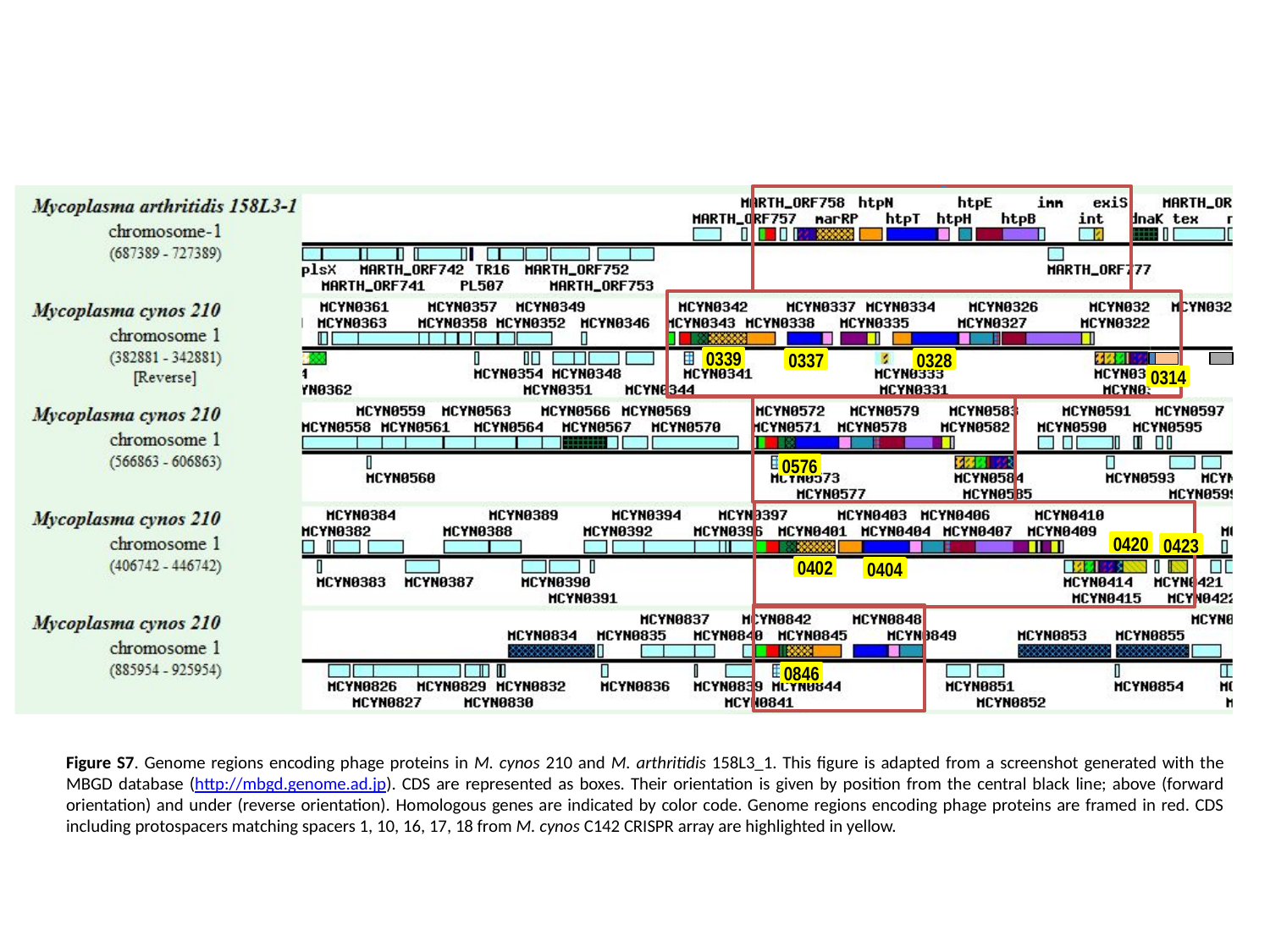

0339
0337
0328
0339
0337
0328
0314
0576
0420
0423
0402
0404
0846
Figure S7. Genome regions encoding phage proteins in M. cynos 210 and M. arthritidis 158L3_1. This figure is adapted from a screenshot generated with the MBGD database (http://mbgd.genome.ad.jp). CDS are represented as boxes. Their orientation is given by position from the central black line; above (forward orientation) and under (reverse orientation). Homologous genes are indicated by color code. Genome regions encoding phage proteins are framed in red. CDS including protospacers matching spacers 1, 10, 16, 17, 18 from M. cynos C142 CRISPR array are highlighted in yellow.
